# Evaluation of the Association of Perioperative *UGT1A1* Genotype–Dosed gFOLFIRINOX With Margin-Negative Resection Rates and Pathologic Response Grades Among Patients With Locally Advanced Gastroesophageal Adenocarcinoma: A Phase 2 Clinical Trial

**DOI:** 10.1001/jamanetworkopen.2019.21290

**Published:** 2020-02-14

**Authors:** Daniel V. T. Catenacci, Leah Chase, Samantha Lomnicki, Theodore Karrison, Robert de Wilton Marsh, Murtuza M. Rampurwala, Sunil Narula, Lindsay Alpert, Namrata Setia, Shu-Yuan Xiao, John Hart, Uzma D. Siddiqui, Bryan Peterson, Kelly Moore, Kristin Kipping-Johnson, Ugne Markevicius, Barbara Gordon, Kenisha Allen, Christine Racette, Steven B. Maron, Chih-Yi Liao, Blase N. Polite, Hedy L. Kindler, Kiran Turaga, Vivek N. Prachand, Kevin K. Roggin, Mark K. Ferguson, Mitchell C. Posner

**Affiliations:** 1 Section of Hematology/Oncology, Department of Medicine, The University of Chicago, Chicago, Illinois; 2 Department of Health Studies, The University of Chicago, Chicago, Illinois; 3 Northshore University Health System, Evanston, Illinois; 4 University of Chicago Medicine, Orland Park, Illinois; 5 University of Chicago Medicine, New Lennox, Illinois; 6 Department of Pathology, The University of Chicago, Chicago, Illinois; 7 Center for Endoscopic Research and Therapeutics, Department of Medicine, The University of Chicago, Chicago, Illinois; 8 Memorial Sloan Kettering Cancer Center, New York, New York; 9 Department of Surgery, The University of Chicago, Chicago, Illinois

## Abstract

**Importance:**

Patients with locally advanced gastroesophageal adenocarcinoma (ie, stage ≥T3 and/or node positive) have high rates of recurrence despite surgery and adjunctive perioperative therapies, which also have high toxicity profiles. Evaluation of pharmacogenomically dosed perioperative gFOLFIRINOX (fluorouracil, leucovorin, oxaliplatin, and *UGT1A1* genotype–directed irinotecan) to optimize efficacy while limiting toxic effects may have value.

**Objective:**

To evaluate the coprimary end points of margin-negative (R0) resection rates and pathologic response grades (PRGs) of gFOLFIRINOX therapy among patients with locally advanced gastroesophageal adenocarcinoma.

**Design, Setting, and Participants:**

This single-group phase 2 trial, conducted at 2 academic medical centers from February 2014 to March 2019, enrolled 36 evaluable patients with locally advanced adenocarcinoma of the esophagus, gastroesophageal junction, and gastric body. Data analysis was conducted in May 2019.

**Interventions:**

Patients received biweekly gFOLFIRINOX (fluorouracil, 2400 mg/m^2^ over 46 hours; oxaliplatin, 85 mg/m^2^; irinotecan, 180 mg/m^2^ for *UGT1A1* genotype 6/6, 135 mg/m^2^ for *UGT1A1* genotype 6/7, or 90 mg/m^2^ for *UGT1A1* genotype 7/7; and prophylactic peg-filgrastim, 6 mg) for 4 cycles before and after surgery. Patients with tumors positive for *ERBB2* also received trastuzumab (6-mg/kg loading dose, then 4 mg/kg).

**Main Outcomes and Measures:**

Margin-negative resection rate and PRG.

**Results:**

A total of 36 evaluable patients (27 [78%] men; median [range] age, 66 [27-85] years; 10 [28%] with gastric body cancer; 24 [67%] with intestinal-type tumors; 6 [17%] with *ERBB2*-positive tumors; 19 [53%] with *UGT1A1 *genotype 6/6; 16 [44%] with genotype 6/7; and 1 [3%] with genotype 7/7) were enrolled. Of these, 35 (97%) underwent surgery; 1 patient (3%) died after completing neoadjuvant chemotherapy while awaiting surgery. Overall, R0 resection was achieved in 33 of 36 patients (92%); 2 patients (6%) with linitis plastica achieved R1 resection. Pathologic response grades 1, 2, and 3 occurred in 13 patients (36%), 9 patients (25%), and 14 patients (39%), respectively, and PRG 1 was observed in 11 of 24 intestinal-type tumors (46%). Median disease-free survival was 30.1 months (95% CI, 15.0 months to not reached), and median overall survival was not reached (95% CI, 8.3 months to not reached). There were no differences in outcomes by *UGT1A1* genotype group. A total of 38 patients, including 2 (5%) with antral tumors, were evaluable for toxic effects. Grade 3 or higher adverse events occurring in 5% or more of patients during the perioperative cycles included diarrhea (7 patients [18%]; 3 of 19 patients [16%] with genotype 6/6; 2 of 16 patients [13%] with genotype 6/7; 2 of 3 patients [67%] with genotype 7/7), anemia (2 patients [5%]), vomiting (2 patients [5%]), and nausea (2 patients [5%]).

**Conclusions and Relevance:**

In this study, perioperative pharmacogenomically dosed gFOLFIRINOX was feasible, providing downstaging with PRG 1 in more than one-third of patients and an R0 resection rate in 92% of patients.

**Trial Registration:**

ClinicalTrials.gov Identifier: NCT02366819

## Key Points

**Question:**

What is the association of perioperative gFOLFIRINOX (fluorouracil, leucovorin, oxaliplatin, and *UGT1A1* genotype–directed irinotecan) therapy with margin-negative resection rates and pathologic response grades among patients with locally advanced adenocarcinoma of the esophagus, gastroesophageal junction, and gastric body?

**Findings:**

In this single-group phase 2 clinical trial of 36 patients, the margin-negative resection rate was 92%, and pathologic response grades 1, 2, and 3 were observed in 36%, 25%, and 39%, respectively, of evaluable participants.

**Meaning:**

In this study, perioperative pharmacogenomically dosed gFOLFIRINOX was tolerable and associated with high rates of margin-negative resection and pathologic response grade 1, which are associated with improved disease-free and overall survival.

## Teaser

This phase 2 trial evaluates the coprimary end points of margin-negative (R0) resection rates and pathologic response grades of gFOLFIRINOX (fluorouracil, leucovorin, oxaliplatin, and *UGT1A1* genotype-directed irinotecan) therapy among patients with locally advanced gastroesophageal adenocarcinoma.

## Introduction

Gastroesophageal adenocarcinoma (GEA), which includes proximal esophagogastric junction (EGJ) and distal gastric adenocarcinomas, remains a global health problem with heterogeneous molecular features.^1,2,3^ Esophagogastric junction adenocarcinomas are predominantly intestinal-subtype and chromosomally instable,^3^ involving the distal esophagus, the gastroesophageal junction (GEJ), and the cardia (ie, so-called Siewert type I, II, and III tumors, respectively).^4^ Esophagogastric junction adenocarcinoma is increasing in incidence in the Western world, while distal gastric adenocarcinoma, including the gastric body, incisura, antrum, and pylorus, is decreasing in incidence.^5^ In Western countries, locally advanced GEA has a high rate of recurrence, and 5-year overall survival (OS) rates remain less than 50%, despite curative-intent surgery with perioperative chemotherapy and/or neoadjuvant chemoradiotherapy.^6^ Adjunctive therapies increase 5-year survival 10% to 15% compared with surgery alone, at the cost of relatively high rates of toxic effects.^6,7,8,9^ Standard therapy for distal gastric adenocarcinoma had been perioperative epirubicin, cisplatin, and fluorouracil (ie, the MAGIC regimen)^7^ until recently, when fluorouracil, leucovorin, oxaliplatin, and docetaxel (FLOT) was shown to be superior.^9^ Standard therapy for EGJ adenocarcinoma now includes perioperative FLOT or neoadjuvant carboplatin and paclitaxel with radiotherapy (40.4 Gy; ie, the CROSS regimen).^8^

Surrogate end points for OS include disease-free survival (DFS)^10^ as well as the more immediate pathologic end points of margin-free (R0) resection^11,12,13^ and pathologic response grade (PRG) 1a (ie, complete response) or 1b (ie, minimal residual disease, <10% residual tumor or tumor bed) by Becker criteria.^14,15,16^ There has been debate whether radiotherapy is required to obtain optimal R0 resection rates in proximal tumors where margins, principally the circumferential margin, might be compromised.^6,11,12,17,18^ In addition to these pathologic end points, an early positron emission tomography (PET) response to neoadjuvant chemotherapy has been associated with better survival,^19,20^ and adjusting neoadjuvant therapy in early PET nonresponders possibly improves clinical outcomes.^20,21,22^ Notably, intestinal-type tumors reportedly have better PET and pathologic response rates after neoadjuvant therapy compared with diffuse-type or mixed-type tumors.^23^ Tumors positive for *ERBB2 *(OMIM 164870) may also derive benefits from the addition of trastuzumab therapy in the perioperative setting.^24^

The FOLFIRINOX regimen, consisting of fluorouracil (400 mg/m^2^ bolus and 2400 mg/m^2^ continuous infusion during 46 hours), leucovorin (400 mg/m^2^), oxaliplatin (85 mg/m^2^), and irinotecan (180 mg/m^2^), was reported to improve outcomes among patients with metastatic pancreatic cancer.^25^ It has also been reported that FOLFIRINOX is efficacious and safe in metastatic GEA, including with trastuzumab for *ERBB2*-positive tumors.^26,27^

The active metabolite of irinotecan, SN-38, is glucuronidated by the enzyme uridine diphosphate glucuronosyltransferase family, polypeptide A1, encoded by the *UGT1A1* gene (OMIM 191740).^28^ The *UGT1A1* gene has germline polymorphisms in exon 1 (variant allele UGT1A1*6, common in Asian individuals) and in the promoter region leading to varying number of TA repeats.^28,29^ The wild-type promoter allele (UGT1A1*1) has 6 TA repeats (genotype 6 or *1 allele). The most common promoter variant allele in white individuals has 7 TA repeats (genotype 7 or *28 allele). Having a genotype with more TA repeats, particularly if homozygous with genotype 7/7, results in less transcription and lower protein expression than the wild-type allele, leading to higher SN-38 levels and a higher risk of toxic effects when receiving irinotecan, including severe neutropenia and dose-dependent severe diarrhea.^30,31^ In 2019, we reported improved tolerability and comparable efficacy to parent FOLFIRINOX using genotype-directed irinotecan dosing (gFOLFIRINOX) along with prophylactic peg-filgrastim and without 5-fluorouracil bolus among patients with first-line metastatic upper gastrointestinal malignant neoplasms, including GEA.^32^ Patients with the heterozygous genotype (ie, 6/7) received a preemptive reduction of irinotecan to 135 mg/m^2^, and patients with the homozygous genotype (ie, 7/7) received 90 mg/m^2^.^32^

The purpose of this study was to prospectively evaluate perioperative gFOLFIRINOX in locally advanced GEA.^33^ The coprimary end points were R0 resection rate and PRG. Secondary end points included safety; PET response rate after neoadjuvant chemotherapy; and DFS, OS, and survival by primary tumor site, histology, PRG, PET response, *ERBB2* status, and *UGT1A1* genotype.

## Methods

### Participants

This single-group phase 2 clinical trial was conducted at 2 academic centers, 1 of which had 2 satellite sites, from February 2014 to March 2019 (ie, date of last adjuvant therapy). The study enrolled patients with locally advanced adenocarcinoma of the esophagus, GEJ, cardia, fundus, and gastric body. Given that patients with antral or pylorus primary tumors generally achieve high rates of R0 resection, they were eligible for treatment but only included in the assessment of toxic effects. Similarly, patients with cytology-positive washings from laparoscopy were eligible for therapy and, if converted to negative cytology after neoadjuvant therapy, could be considered for surgery; these patients were not included in the primary efficacy or toxic effects analyses. The study was approved by the University of Chicago institutional review board, and patients provided written informed consent. This study followed the Transparent Reporting of Evaluations With Nonrandomized Designs (TREND) reporting guideline.

Inclusion criteria included biopsy-proven adenocarcinoma eligible for surgery with curative intent if considered locally advanced with a T stage of 3 or higher or any T stage with node-positive disease based on standard diagnostic testing, including endoscopic ultrasound, computerized tomography (CT), PET scan, and diagnostic laparoscopy. Eligible patients had Eastern Cooperative Oncology Group performance status of 0 or 1 and were older than 18 years, with adequate hematologic function (ie, absolute neutrophil count, ≥1250/μL [to convert to ×10^9 ^per liter multiply by 0.001]; hemoglobin, ≥9 g/dL [to convert to grams per liter multiply by 10.0]; and platelets, ≥100 × 10^3^/μL [to convert to ×10^9 ^per liter multiply by 1.0]), renal function (creatinine ≤1.5 times the upper limit of normal), and hepatic function (bilirubin <1.5 times the upper limit of normal). Patients with *ERBB2*-positive tumors were required to have a normal cardiac ejection fraction. Key exclusion criteria for efficacy analyses included distal gastric cancers (eg, antrum, pylorus) and metastatic disease, prior therapy for GEA, previous or concurrent malignant neoplasm except for adequately treated basal cell or squamous cell skin cancer, in situ cervical cancer, or any other cancer for which the patient had been previously treated and the lifetime recurrence risk was considered less than 30%, uncontrolled or active treatment for inflammatory bowel disease, baseline diarrhea grade 1 or higher, and baseline neuropathy grade 2 or higher.

### *UGT1A1* Genotyping

Analyses of *UGT1A1* polymorphism for the promoter TA repeat and exon 1 loci were performed at the University of Chicago as previously described.^30,32^ Patients were grouped into high-risk, intermediate-risk, and low-risk *UGT1A1* groups, as previously described,^28,34^ and these were generally represented by the 7/7, 6/7, and 6/6 genotypes, respectively.

### Neoadjuvant gFOLFIRINOX

Neoadjuvant gFOLFIRINOX was administered for 4 biweekly cycles. Bolus leucovorin (400 mg/m^2^), oxaliplatin (85 mg/m^2^), and genotype-dosed irinotecan (180 mg/m^2^ for genotype 6/6, 135 mg/m^2^ for genotype 6/7, and 90 mg/m^2^ for genotype 7/7) were administered on day 1 of each cycle. Fluorouracil was administered only as a 2400 mg/m^2^ continuous infusion for 46 hours (no bolus). Patients with *ERBB2*-positive tumors were dosed first with trastuzumab at 6 mg/kg on cycle 1, then at 4 mg/kg on cycles 2 to 4. Prophylactic peg-filgrastim (6 mg) was administered on day 3 of every cycle. Dose adjustments for toxic effects were defined in the protocol (Supplement 1).

### PET Imaging

The change in maximum standardized uptake value (SUVmax) of the primary tumor between the baseline and postneoadjuvant therapy PET studies was calculated and expressed as percentage change. The change in SUVmax between the baseline and posttherapy PET studies was assessed only if the tumor-to-background SUV was greater than 1.5 on baseline imaging. Generally, this corresponded with an SUVmax of 5 or greater.

### Surgery

Surgery was performed 4 to 6 weeks after the last dose of neoadjuvant gFOLFIRINOX. The surgical approach was determined per routine clinical standards and included transthoracic esophagectomy with 2-field lymphadenectomy, transhiatal esophagectomy with lower mediastinal and upper abdominal lymphadenectomy, and proximal, subtotal, or total gastrectomy with D2 lymphadenectomy.

### Pathologic Evaluation

Pathologic specimens were scored per standard institutional practices and College of American Pathologists guidelines.^11,12^ Resection margins were considered negative if microscopic tumor was not present at the inked margins. Primary tumor regression was graded by the amount of viable tumor vs fibrosis, ranging from no evidence of any treatment effect to a complete response with no viable tumor.^14^ Grades were classified as follows: grade 1a, complete remission, no residual tumor or tumor bed; grade 1b, subtotal remission, less than 10% residual tumor or tumor bed; grade 2, partial remission, 10% to 50% residual tumor or tumor bed; and grade 3, minor or no remission, greater than 50% residual tumor or tumor bed.

### Adjuvant gFOLFIRINOX

Patients were assessed postoperatively. They resumed therapy between 5 and 10 weeks after surgery, as they were able and treatment was tolerated, for another 4 planned cycles.

### Follow-up

To document patterns of recurrence after completion of all planned therapy, patients had surveillance follow-up visits with laboratory evaluation every 3 months, CT scans every 6 months, and an annual upper endoscopy for the first 3 years. For 2 more years after surgery, patients had laboratory evaluation every 6 months and received an annual CT scan and endoscopy.

### Statistical Analysis

The study was designed to detect a 20% improvement in the R0 resection rate from 70% to 90% with perioperative chemotherapy. These values were based on published surgical experiences with GEA at the time of designing this study (ie, 69%-74% with surgery alone and 79%-100% with neoadjuvant chemotherapy or chemoradiotherapy).^7,8,35,36,37,38,39^

Patient enrollment followed an optimal 2-stage design,^40^ with an α level of .05 and power of 0.90. Accrual would have been halted if 11 or fewer of the initial 15 assessable patients (ie, <73%) achieved R0 resections. In the second stage, 21 additional patients were enrolled, for a total of 36 patients. The treatment would be considered active and worthy of additional investigation at the end of the study if an R0 was achieved in at least 30 of 36 assessable patients (ie, >83.3%) in the intention-to-treat (ITT) population. Patients with tumor progression during or after neoadjuvant chemotherapy or death that precluded surgery would be considered non-R0 resections. A preplanned modified ITT subset analysis would be performed to evaluate the R0 rate among patients who underwent surgery and were treated neoadjuvantly, per protocol.

A coprimary end point was pathologic complete response rate (ie, grade 1a). A sample size of 36 patients achieved 85% power at α level .05 to detect an absolute 13% improvement using a 1-sided binomial test. These results assumed that the population proportion under the null hypothesis was P0 = 0.03. This rate was consistent with described rates of complete remission for epirubicin, cisplatin, and fluorouracil or cisplatin and fluorouracil (ie, 3%-4%) at the time of trial design.^7,35^ We would reject the null hypothesis and accept the alternate hypothesis (P1 = 0.16) if there were an observed grade 1a in 4 or more of 36 patients (ie, ≥11.1%).

Toxic effects were summarized by type, grade, and attribution. The secondary end points of OS and DFS were estimated using the Kaplan-Meier procedure,^41^ and subgroup comparisons by tumor histology, location, pathologic lymph node status, *UGT1A1* genotype, *ERBB2* status, PET response, and PRG were performed using the log-rank test. The software used was Stata version 16.0 (StataCorp). Statistical signficance was set at *P* < .05, and all tests were 2-tailed.

Only 1 patient died before surgery, and no patients progressed before surgery. The PRG response for the patient who died after completing all neoadjuvant cycles was determined at autopsy. All patients’ PET responses were assessed after completion of their neoadjuvant chemotherapy. Therefore, analyses of DFS and OS by PET and PRG responses were not subject to lead-in bias.

## Results

### Patient Characteristics

**Figure 1.**
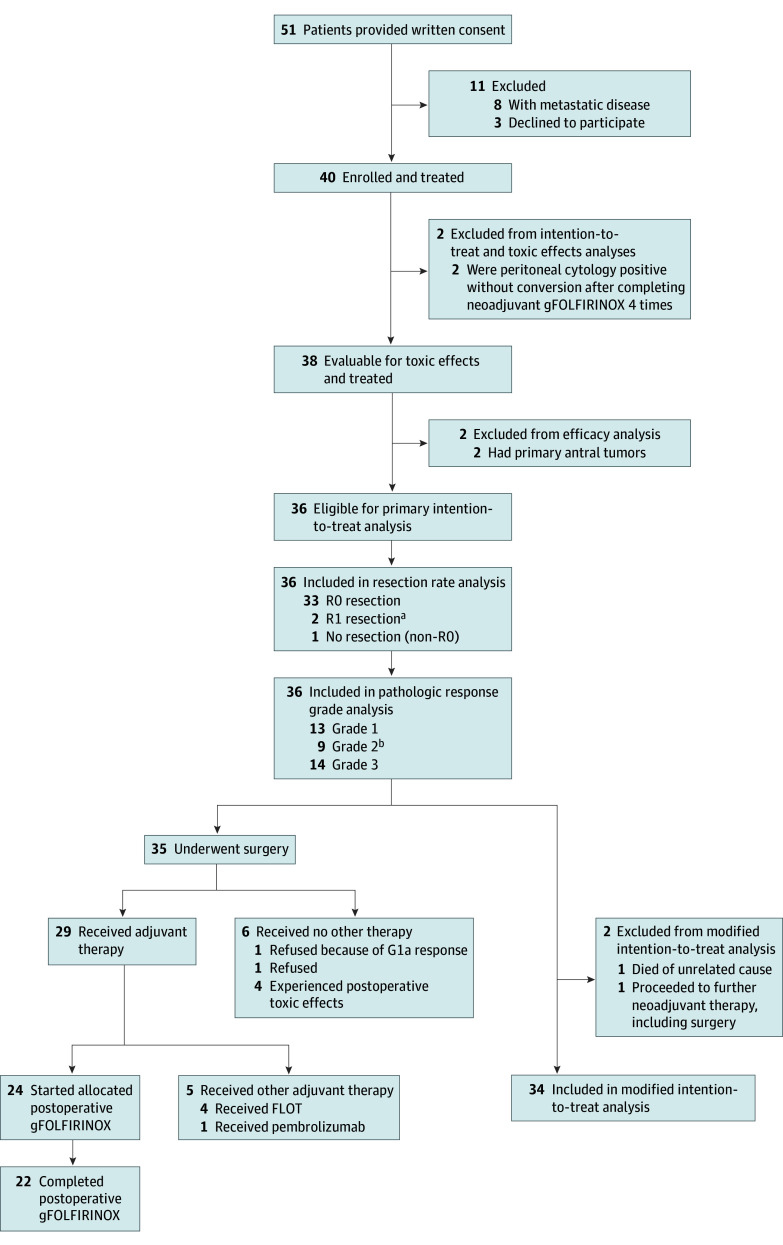
Study Flow Diagram FLOT indicates fluorouracil, leucovorin, oxaliplatin, and docetaxel; G1a, grade 1a, complete response; gFOLFIRINOX, fluorouracil, leucovorin, oxaliplatin, and *UGT1A1* genotype–directed irinotecan; R0, margin-negative. ^a^Both patients with R1 resection had gastric linitis plastica. ^b^A patient with pathologic response grade 2 died before surgery but was evaluable by autopsy.

Of the 40 patients enrolled in the study from February 2014 to March 2019 (Figure 1), 2 patients (5%) were not eligible for efficacy or toxic effect analyses because of positive cytology washings at diagnosis. Neither patient converted to negative cytology after completing neoadjuvant therapy and, thus, were treated with palliative intent thereafter. The remaining 38 patients were evaluable for safety; however, per protocol, 2 (5%) were excluded from primary efficacy analyses given that they had primary antral tumors (1 [50%] mixed-type and 1 [50%] diffuse-type; both 7/7 genotype), and both achieved R0 resection and demonstrated PRG 3. Baseline patient clinicopathologic and genotyping characteristics for 36 evaluable patients (27 [78%] men; median [range] age, 66 [27-85] years; 10 [28%] with gastric body cancer; 24 [67%] with intestinal-type tumors; 6 [17%] with *ERBB2*-positive tumors; 19 [53%] with *UGT1A1 *genotype 6/6; 16 [44%] with genotype 6/7; and 1 [3%] with genotype 7/7) were comparable to the recent FLOT4 study^9^ (Table 1; eTable 1 and eTable 2 in Supplement 2).

**Table 1.** Baseline Clinicopathologic Characteristics of 36 Patients Evaluable for Primary Efficacy End Points

Characteristic	No. (%)
Age, median (range), y	66 (27-85)
Sex	
Men	27 (75)
Women	9 (25)
Primary tumor location	
EGJ Siewert type 1	6 (17)
EGJ Siewert type 2 or 3	20 (56)
Stomach	10 (28)^a^
Signet ring cells	
Present	10 (28)
Absent	26 (72)
Tumor differentiation	
G1, well differentiated	1 (3)
G2, moderately differentiated	9 (25)
G3, poorly differentiated	26 (72)
Clinical T stage	
1	0
2	5 (14)
3	31 (86)
4	0
Clinical N stage	
N negative	9 (25)
N positive	27 (75)
*ERBB2* status	
Positive	6 (17)
Negative	30 (83)
*UGT1A1* genotype	
6/6	19 (53)
6/7	16 (44)
7/7	1 (3)

Abbreviations: EGJ, esophagogastric junction; N stage, nodal stage; T stage, tumor stage.

^a^ Only gastric body because more distal tumors in the antrum or pylorus were not eligible for efficacy analysis.

### Treatment Completion Rates and Safety

All 38 patients evaluable for safety (Figure 1) completed all 4 cycles of neoadjuvant therapy. The 2 patients (5%) with antral tumors, both with 7/7 genotypes, received all 4 cycles of adjuvant therapy (1 patient [50%] received only fluorouracil for 4 cycles because of preoperative toxic effects). Of 36 patients evaluable for efficacy, 1 (3%) died 4 weeks after completion of neoadjuvant therapy while awaiting surgery; their death was deemed unrelated to chemotherapy or cancer. Another patient (3%) without clinical improvement or PET response proceeded with further neoadjuvant carboplatin and paclitaxel plus radiotherapy, followed by surgery and no further adjuvant therapy. These 2 patients (6%) were included in ITT efficacy analyses. Of the remaining 34 patients, 29 (85%) received adjuvant therapy of any kind. Postoperatively, 24 of 36 patients (67%) in the evaluable efficacy cohort initiated adjuvant gFOLFIRINOX therapy, of whom 22 (91.7%) completed all 4 cycles. A total of 5 patients (14%) received other adjuvant therapies, including FLOT (4 [11%]), which was allowed per protocol if PRG 3 was observed and per physician discretion, and pembrolizumab (1 [3%]). During the 8 perioperative cycles, the percentage of planned therapy administered among the 38 patients was as follows: fluorouracil, 641 700 mg/m^2^ of expected 729 600 mg/m^2^ (88.0%); leucovorin, 103 360 mg/m^2^ of expected 121 600 mg/m^2^ (85.0%); irinotecan, 42 675 mg/m^2^ of expected 46 800 mg/m^2^ (82.3%); and oxaliplatin, 21 740 mg/m^2^ of expected 25840 mg/m^2^ (84.1%). These percentages include fluorouracil and oxaliplatin among the 4 patients receiving adjuvant FLOT. Percentages of planned therapy delivered preoperatively vs postoperatively and by *UGT1A1* genotype are indicated in eTable 3 in Supplement 2; most dose reductions and toxic effects occurred postoperatively. Of 6 patients (17%) who did not receive adjuvant therapy, 4 (67%) were considered ineligible because of postoperative complications precluding therapy, and 2 (33%) refused adjuvant therapy, 1 (17%) because of PRG 1a response to neoadjuvant therapy.

Common Terminology Criteria for Adverse Events toxic effects criteria, version 4.0, were used for grading. Grade 3 or higher adverse events occurring in 5% or more of patients during neoadjuvant cycles included diarrhea (7 patients [18%]; 3 of 19 [16%] with genotype 6/6, 2 of 16 [13%] with genotype 6/7, and 2 of 3 [67%] with genotype 7/7), anemia (2 patients [5%]), vomiting (2 patients [5%]), and nausea (2 patients [5%]) (Table 2). Toxic effects were comparable to those reported in the FLOT4 study,^9^ with more diarrhea and less neuropathy, alopecia, and neutropenia (eTable 4 in Supplement 2).

**Table 2.** Grade 3 or Higher Toxic Effects Associated With Perioperative gFOLFIRINOX, by *UGT1A1* Genotype

CTCAE Term	Patients, No. (%)			
	Total (N = 38)	*UGT1A1* 6/6 (n = 19)	*UGT1A1* 6/7 (n = 16)	*UGT1A1* 7/7 (n = 3)
Diarrhea	7 (18)	3 (16)	2 (13)	2 (67)
Vomiting	2 (5)	1 (5)	0	1 (33)
Nausea	2 (5)	2 (11)	0	0
Dehydration	3 (8)	2 (11)	1 (6)	0
Anemia	2 (5)	0	1 (6)	1 (33)
Neuropathy	0	0	0	0
Alopecia, grade 2	0	0	0	0
Thrombocytopenia	0	0	0	0
Neutropenia	0	0	0	0
Febrile neutropenia	0	0	0	0

Abbreviations: CTCAE, Common Terminology Criteria for Adverse Events; gFOLFIRINOX, fluorouracil, leucovorin, oxaliplatin.

### PET Response Evaluation

Radiographic PET response was measured as percentage change in SUVmax from baseline to after neoadjuvant therapy. Of 27 evaluable patients (ie, those with both baseline and follow-up scans who met the SUVmax baseline criteria of eligibility), 24 (89%) achieved a response of 35% or higher. Of these, 10 (42%) had complete responses (Table 3; eTable 5 and eFigure 1 in Supplement 2). The PET response rate did not differ by *UG1A1* genotype group.

**Table 3.** Primary Efficacy Analysis of R0 Resection and Pathologic Response Grade in the ITT Analysis and Preplanned Secondary Subgroups

Group	Incidence, No. (%)	Patients, No./Total No. (%)						
		R0 Resection Rate	Pathologic Response Grade					PET Response ≥35% SUVmax^a^
			1a and 1b	1a	1b	2	3	
ITT population	36 (100)	33/36 (92)	13/36 (36)	3/36 (8)	10/36 (28)	9/36 (25)	14/36 (39)	24/27 (89)
Tumor location								
EGJ	26 (72)	25/26 (96)	10/26 (38)	2/26 (8)	8/26 (31)	8/26 (31)	8/26 (31)	21/23 (91)
GB	10 (28)	8/10 (80)	3/10 (30)	1/10 (10)	2/10 (20)	1/10 (10)	6/10 (60)	3/4 (75)
Histology								
Intestinal	24 (67)	22/24 (96)	11/24 (46)	3/24 (13)	8/24 (33)	5/24 (21)	8/24 (33)	18/20 (90)
Mixed-type or diffuse-type	12 (33)	10/12 (83)	2/12 (17)	0	2/12 (17)	4/12 (33)	6/12 (50)	6/7 (86)
*ERBB2* status								
Positive	6 (17)	6/6 (100)	3/6 (50)	1/6 (17)	2/6 (33)	2/6 (33)	1/6 (17)	6/6 (100)
Negative	30 (83)	27/30 (90)	10/30 (33)	2/30 (7)	8/30 (27)	7/30 (23)	13/30 (43)	18/21 (86)
*UGT1A1* genotype								
6/6	19 (53)	17/19 (89)	6/19 (32)	3/19 (16)	3/19 (16)	6/19 (32)	7/19 (37)	14/15 (93)
6/7	16 (44)	15/16 (94)	7/16 (44)	0/16 (0)	7/16 (44)	3/16 (19)	6/16 (38)	10/12 (83)
7/7	1 (3)	1/1 (100)	0	0	0	0	1/1 (100)	NA^b^
PET responders	24/27 (89)	23/25 (92)	12/12 (100)	3/3 (100)	9/9 (100)	7/7 (100)	5/8 (63)	NA

Abbreviations: EGJ, esophagogastric junction; GB, gastric body; ITT, intention to treat; PET, positron emission tomography; NA, not applicable; R0, margin negative; SUVmax, maximum standard uptake value.

^a^ Patients without baseline SUVmax greater than or equal to 5 were not evaluable; patients without both a pretherapy and posttherapy PET scan were not evaluable.

^b^ Not evaluable given that this patient’s tumor was diffuse-type and did not have baseline PET activity.

### Surgery Details

Of the 36 patients evaluable for efficacy, 35 (97%) underwent curative-intent surgery successfully (Figure 1). Details regarding the surgical procedures performed on all 37 patients evaluable for safety are in eTable 6 in Supplement 2. The median (range) number of lymph nodes removed was 24 (19-28). Within 30 days after surgery, 8 of 37 patients (22%) were either readmitted to the hospital with complications and/or had additional procedures. Anastomotic leak occurred in 3 of 23 proximal tumors (13%); 2 patients (5%) had endoscopy with dilation of the anastomosis. There was no 30-day postoperative mortality.

### Pathologic R0 Resection Rates

Among 36 evaluable patients, 35 (97%) underwent resection. One patient with EGJ (3%) died before surgery and was considered non-R0 resection. Another patient with EGJ (3%) received chemoradiotherapy after completing gFOLFIRINOX and achieved R0 resection (included in ITT analysis). Two of the remaining 34 patients (6%), both with linitis plastica, had R1 resections. Therefore, 33 of the 36 patients (92%) in the ITT population achieved R0 resection, including all Simon-stage I patients, meeting the study design criteria for a positive result (Table 3). Of 26 proximal EGJ tumors, 25 (96%) achieved R0 resection, all except the patient who died before surgery. A preplanned modified ITT analysis resulted in R0 resection in 32 of 34 patients (94%) (Figure 1). Results compared favorably with the results from the FLOT4^9^ and CROSS^8^ studies (eTable 7 in Supplement 2), and the R0 resection rate did not differ by *UG1A1* genotype group.

### PRG Rates

Among 36 evaluable patients, 13 (36%) achieved a PRG 1 by ITT, of whom 3 (23%) were PRG 1a and 10 (77%) were PRG 1b (Table 3). Therefore, the coprimary end point of grade 1a response in 4 or more patients was not achieved. The patient who died while awaiting surgery demonstrated PRG 2 on autopsy, and the patient receiving chemoradiotherapy also had PRG 2; both were included in the ITT analysis. Pathologic response grade 1 was observed in 11 of 24 intestinal-type tumors (46%). All PRG 1a responses were observed in intestinal-type tumors (3 of 24 [12%]) and in PET responders with at least a 75% decrease in SUVmax (eTable 5 in Supplement 2). Overall, PRGs were favorable compared with those in the FLOT4 study^9^ (eTable 8 in Supplement 2), and PRGs did not differ by *UG1A1* genotype group.

### Survival and Patterns of Relapse

**Figure 2.**
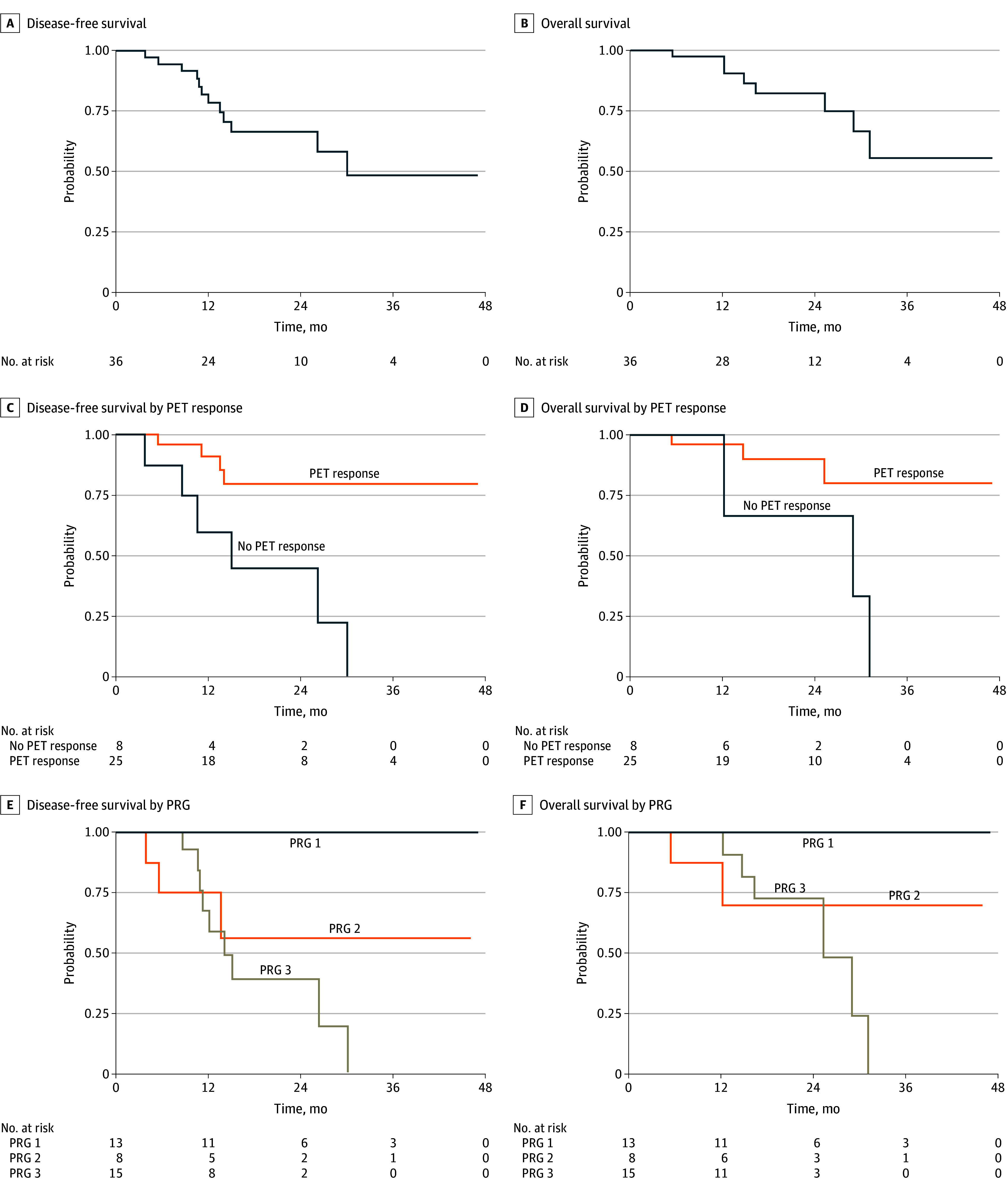
Disease-Free Survival and Overall Survival A, Median disease-free survival, 30.1 months (95% CI, 15.0 months to not reached). B, Median overall survival not reached (95% CI, 8.3 months to not reached). C, Patients achieving positron emission tomography (PET) maximum standard uptake value response (ie, ≥35% decrease) after neoadjuvant chemotherapy demonstrated improved outcomes compared with those without maximum standard uptake value response (*P* = .003). D, Patients achieving PET maximum standard uptake value response (ie, ≥35% decrease) after neoadjuvant chemotherapy demonstrated improved outcomes compared with those without maximum standard uptake value response (*P* = .02). E, Patients with pathological response grade (PRG) 1 vs PRG 2 vs PRG 3 were significantly different (*P* = .003). F, Patients with PRG 1 vs PRG 2 vs PRG 3 were significantly different (*P* = .02).

Median (range) follow up was 19.7 (8.3-47.1) months and 21.7 (8.3-47.1) months among patients still disease free and alive, respectively. Of 36 evaluable patients, 11 (31%) experienced disease recurrence, all with at least 1 distant metastatic site (eTable 9 in the Supplement 2), and 7 of these patients (64%) plus 1 other patient died (ie, 8 of 36 [22%]). Both patients with R1 resections died within 1 year after surgery. The median DFS was 30.1 months (95% CI, 15.0 months to not reached) (Figure 2A), and median OS was not reached (95% CI, 8.3 months to not reached) (Figure 2B). Preplanned subgroups showed significant differences in DFS and OS by PET response (Figure 2C and Figure 2D) and PRG (Figure 2E and Figure 2F); histology, anatomical site, pathologic lymph node status, *ERBB2* status, and *UGT1A1* genotype subgroup did not demonstrate significant differences (eFigure 2 and eTable 10 in Supplement 2).

## Discussion

When designing this trial, the perioperative MAGIC regimen was standard of care for GEA,^7^ and neoadjuvant chemoradiotherapy with the CROSS regimen was an alternative for EGJ tumors.^8^ A number of phase 2 studies had demonstrated efficacy with fluoropyrimidine, platinum, and taxane regimens compared with historical outcomes with MAGIC.^36,37,38,39^ However, given the toxic effects of both MAGIC and taxane-based triplet regimens, particularly neuropathy and alopecia, we investigated whether gFOLFIRINOX was a more tolerable option, with nonoverlapping toxic effects and with better or similar outcomes compared with MAGIC and taxane-based regimens, respectively. At the time, FOLFIRINOX had demonstrated efficacy for metastatic pancreatic cancer.^25^ Approximately two-thirds of the way into the accrual period of this study, the FLOT4 study reported improved outcomes with FLOT compared with MAGIC.^9,23^ Despite this, we continued accrual to completion with the rationale described earlier. Also in the interim, modified FOLFIRINOX improved survival in the adjuvant setting of pancreatic cancer, with necessary but indiscriminate dose-reduction of irinotecan to 150 mg/m^2^ for all patients as well as no fluorouracil bolus.^42^ This and other variations of the parent FOLFIRINOX regimen, including 25% dose reduction of the fluoropyrimidine bolus and irinotecan^43^ or dropping the fluoropyrimidine bolus altogether while adding prophylactic peg-filgrastim leukocyte growth factor,^44,45^ have improved tolerability without demonstrable loss of efficacy in various settings. To our knowledge, this was the first study to assess FOLFIRINOX perioperatively for GEA and the first to study pharmacogenomic gFOLFIRINOX in the perioperative scenario for any cancer. Our results demonstrated that preemptive irinotecan dose reduction in individuals with high risk, as determined by *UGT1A1* genotype, improved overall tolerability and cumulative dosing compared with prior perioperative GEA studies, given that participants in this study experienced higher treatment completion rates and less neurotoxicity and alopecia^7,9,16,35,46^ without any appreciable compromised efficacy. This regimen could be of particular importance for patients with baseline neuropathy or high risk of developing neuropathy, such as those with long-standing diabetes. Moreover, individuals with low risk (ie, with genotype 6/6) tolerated the standard dosing well, as previously reported.^32,47^ In fact, despite lower irinotecan doses, patients with genotypes 6/7 and 7/7 experienced similar or higher rates of diarrhea and further dose modifications compared with standard dosing in patients with the genotype 6/6, supporting genotype-directed dosing for these patient groups.

We assessed the association of perioperative gFOLFIRINOX therapy with R0 rates and PRG in patients with locally advanced tumors; both are accepted surrogate end points for DFS and OS.^11,12,13,14,16,23^ We did not include distal gastric antral or pylorus primary tumors, for which R1 resection is generally uncommon. Rather, we focused on proximal tumors, for which there is concern for obtaining clear resection margins particularly in the absence of neoadjuvant radiotherapy.^6,17,18^ In the present study, the R0 resection rate was 92% among 36 evaluable patients and 96% among patients with proximal EGJ tumors. Even when considering the number of patients who received chemoradiotherapy before surgery as non-R0 (ie, 32 of 36 [89%]), these results are comparable with, if not better than, standard therapy with the FLOT^9^ or CROSS^8^ regimens. The only patients with an actual R1 resection had primary gastric tumors with linitis plastica. Although the PRG 1a rate was lower in this study than in the FLOT study,^9^ the observed PRG 1a and PRG 1b responses of 36% of patients were better than MAGIC^7^ (ie, 20% of patients) and slightly better than FLOT^9^ (ie, 32% of patients). We observed that both PRG 1a and PRG 1b resulted in excellent long-term outcomes with no recurrences to date or appreciable differences between them, consistent with other reports.^16^ Not all PRGs were reported from the CROSS study,^8^ and therefore, full comparisons cannot be made. Instead, only PRG 1a response was reported among 21% to 23% of the adenocarcinoma subgroup. Importantly, given that it is well known that more than 90% of patients who ultimately have recurrence will do so with distant metastatic disease with or without radiotherapy, a PRG 1 is likely more meaningful if achieved with potent triplet systemic therapy alone (which affects microscopic systemic disease sites simultaneously) compared with doublet chemotherapy plus radiotherapy, which confers less potent systemic control. Notably, in our study without radiotherapy, all patients experiencing recurrence developed distant metastatic disease, further supporting the notion that local recurrence is not the primary driver of poor outcomes. Furthermore, PET response was associated with better prognosis, and the PET response rate was 89% in our study, and it was 91% in EGJ tumors; to our knowledge, this is the highest PET response reported to date.^19,20,21^ With the few PET nonresponders having high risk for distant recurrence and poor prognosis, novel options are needed. Trastuzumab added to gFOLFIRINOX for *ERBB2*-positive tumors demonstrated higher PRG 1 compared with *ERBB2*-negative tumors (50% vs 33%) (Table 3), similar to a previous report^24^; however, this did not translate into a survival advantage, likely because of small numbers.

### Limitations

This study has limitations. Lack of randomization in a single-group phase 2 study is a recognized limitation. However, the baseline clinicopathologic characteristics are comparable with the recent FLOT4 randomized clinical trial.^9^ Higher rates of poor prognostic variables were present compared with the CROSS study,^8^ including higher clinically staged node-positive disease and more diffuse-type and mixed-type tumors. Another possible limitation is the heterogeneity of treatment dosing among 3 *UGT1A1* genotype groups and the *ERBB2*-positive patients treated with trastuzumab, making each subgroup individually difficult to study. However, the *ERBB2*-positive subgroup accounted for only 17% of patients, with outcomes only slightly better than the *ERBB2*-negative group. Regardless, the focus of this study was the treatment strategy,^48,49^ and the overall ITT R0 end point, a composite of the subsets, was met.

## Conclusions

In this prospective evaluation of gFOLFIRINOX, the regimen was tolerable and showed a high rate of R0 resection, PRG, and PET response, each associated with prolonged survival rates. These results support further investigation of gFOLFIRINOX perioperatively for locally advanced GEA, a valuable therapeutic option for patients.^50^

## Notes

Supplement 1.
Trial Protocol

Supplement 2. **eTable 1. **Patient and Tumor Characteristics at Baseline in 36 Evaluable Patients Compared With the FLOT4 Study Arms **eTable 2. ***UGT1A1* Genotype Distribution by Ethnicity in 38 Evaluable Patients **eTable 3. **Percentage of Total Planned Doses of Chemotherapeutic Drugs Neoadjuvantly vs Adjuvantly by *UGT1A1* Genotype in 38 Evaluable Patients **eTable 4. **Perioperative Toxic Effects of Grade 3 or Higher Associated With gFOLFIRINOX in 38 Evaluable Patients Compared With the FLOT4 Study Arms **eTable 5. **Absolute Changes in SUVmax by PET in 27 Evaluable Patients and Associations With Other Clinical and Pathologic Characteristics **eTable 6. **Details Regarding 37 Patients Who Underwent Curative-Intent Surgery **eTable 7. **Surgical and Pathological Results in 36 Evaluable Patients Compared With the FLOT4 and CROSS Study Arms **eTable 8. **Pathological Response Grade Analysis in 36 Evaluable Patients Compared With the FLOT4 Study Arms **eTable 9. **Patterns of Recurrence in 11 Patients Demonstrating Metastatic Recurrence in All Patients **eTable 10. **Disease-Free and Overall Survival Analyses by Subgroup **eFigure 1. **Waterfall Plot of Percentage Change in SUVmax from Neoadjuvant gFOLFIRINOX Therapy **eFigure 2. **Disease-Free and Overall Survival of 36 Evaluable Patients by Histology, Primary Tumor Anatomical Site, Lymph Node Involvement at Surgery, *ERBB2* Status, and *UGT1A1* Subgroup
eReferences.

Supplement 3.
Data Sharing Statement
