# Supplementary material for: Evaluation of the Association of Perioperative UGT1A1 Genotype–Dosed gFOLFIRINOX With Margin-Negative Resection Rates and Pathologic Response Grades Among Patients With Locally Advanced Gastroesophageal Adenocarcinoma: A Phase 2 Clinical Trial
Source: JAMA Netw Open. 2020 Feb 14;3(2):e1921290. doi: 10.1001/jamanetworkopen.2019.21290 (PMC12549139; doi:10.1001/jamanetworkopen.2019.21290)
Supplement: Supplement 1. — Trial Protocol [file jamanetwopen-e1921290-s001.pdf]

TITLE: IRB 14-0594 **PERIOP-FOLFIRINOX: A pilot trial of perioperative genotype-guided irinotecan dosing of gFOLFIRINOX for locally advanced gastroesophageal adenocarcinoma**

Version 7-3-19

Principal Investigator: Daniel Catenacci, MD
5841 S. Maryland Avenue
MC 2115
Chicago, IL 60637-1470
P: 773-702-7596
F: 773-702-3163
dcatenac@medicine.bsd.uchicago.edu

Summary of Amendment: Expansion Cohort 2

1. **Given the results of the original study 'cohort 1' of 36 patients demonstrating excellent R0 resection rates better than current standard R0 rates, and also given the high pathologic response grades, particularly in the intestinal type tumors (24 of the 36 patients enrolled), as well as promising disease free and overall survival, we will amend the protocol to allow for more accrual specifically in the intestinal histology (having both intesintal and diffuse type components). This gFOLFIRINOX regimen proved to be safe and tolerable, and also had outcomes comparable to standard options of FLOT and chemoradiotherapy with carboplatin/paclitaxel (Catenacci et al. ASCO 2019), and is therefore our preferred regimen given better tolerability.**

2. **Expansion cohort 2 sample size calculation:**
   We will enroll 29 new patients with intestinal type histology to reject the null hypotesis that the pathologic response (grade 1) is 20% as in historical controls, and accept the alternative hypothesis of 45% pathologic response grade 1. For this expansion cohort 2, ≥10 of 29 would independently confirm propectively our previous result of 11/24 (45.8%) from the original cohort 1. At completion of the expansion cohort 2, this would leave us with 53 total patients with intestinal type, with 29 of them in the prospective expansion cohort 2 and 24 in the original cohort 1. With these 53 patients pooled from cohort 1 and cohort2, we will also pool them to help to provide a more precise estimate of Grade 1 pathologic response in intestinal type via the 95% CI with the larger total number.

3. **Eligibility will remain the same as in the previous protocol. A checklist (Appendix E) has been added to the protocol to be used by all enrolling sites to document eligibility and sent to Dr. Catenacci to be signed off prior to enrollment. As we will have our primary stastitical endpoint for the intestinal type gastric body/esophagogastric cancers only (n=29) we will still allow mixed or pure diffuse histology as well as antral cancers as in cohort 1 to accrue for exploratory purposes, and we estimate that this will be no more than 15 patients, so the total cohort 2 will include up to 44 patients (29 intention to treat + 15 mixed/antral tumors).**

4. **Given the findings from the cohort 1 original report, where no patient had progression from baseline to pre-surgery, we will not mandate post-treatment diagnostic laparoscopy prior to surgery nor endoscopic ultrasound, but either can be performed if clinically indicated.**

We will add aquisition of blood for research purposes to be obtained in 2 Streck tubes at 4-5 time points (pre-neoadjuvant therapy, post-neoadjuvant therapy, post-surgery, and post-adjuvant therapy, and if cancer recurs). These will be used in the future for exploratory analyses regarding circulating tumor DNA (ctDNA) and associations with prognosis. Section 6.4 Tubes will be sent to HTRC and processed centrally, along with Appendix F Blood Banking Form.

Patient eligibility:

1. Histologically confirmed locally advanced gastric (primary endpoint includes proximal and mid-body stomach) or esophagogastric adenocarcinoma (intestinal type). Distal gastric (antral) adenocarcinomas, and mixed or diffuse type histology are each eligible for enrolment but will not be included in the primary analysis.
2. Locally advanced disease as determined by EUS stage $\geq$T3 and/or any T, N+ disease without metastatic disease (Mx).
3. All patients must have diagnostic laparoscopy with diagnostic washings for cytology. Both Cytology positive for maligancy and negative patients are eligible for enrolment, but only cytology negative patients will be included in the primary analyses. Gross peritoneal disease is not eligible.
4. HER2 positive and negative patients are eligible.
5. Cardiac Ejection Fraction $\geq$50% (for HER2+ patients) as assessed by echocardiogram, MUGA scan or cardiac MRI
6. Age ≥18 years.
7. Eastern Cooperative Oncology Group (ECOG) performance status ≤ 1 (see Appendix A).
8. Eligible for surgery with curative intent.
9. Adequate organ function, as defined by each of the following:
   Absolute neutrophil count (ANC) ≥ l250/μl
   Hemoglobin ≥ 9g/dL
   Platelets ≥ 100,000/ μl)
   Total bilirubin < 1.5 x upper limit of normal
   SGOT and SGPT < 2.5 x upper limit of normal
   Creatinine ≤ 1.5 x upper limit of normal
10. Measurable or non-measurable disease by RECIST 1.1 will be allowed.
11. Women of child-bearing potential and men must agree to use adequate contraception (hormonal or barrier method of birth control; abstinence) prior to study entry and for the duration of study participation, up until 30 days after final study treatment. Should a woman become pregnant or suspect that she is pregnant while participating in this study, she should inform her treating physician immediately.
12. Patients taking substrates, inhibitors, or inducers (see Appendix C) of CYP3A4 should be encouraged to switch to alternative drugs whenever possible, given the potential for drug-drug interactions with irinotecan.
13. Signed informed consent.

Schema:

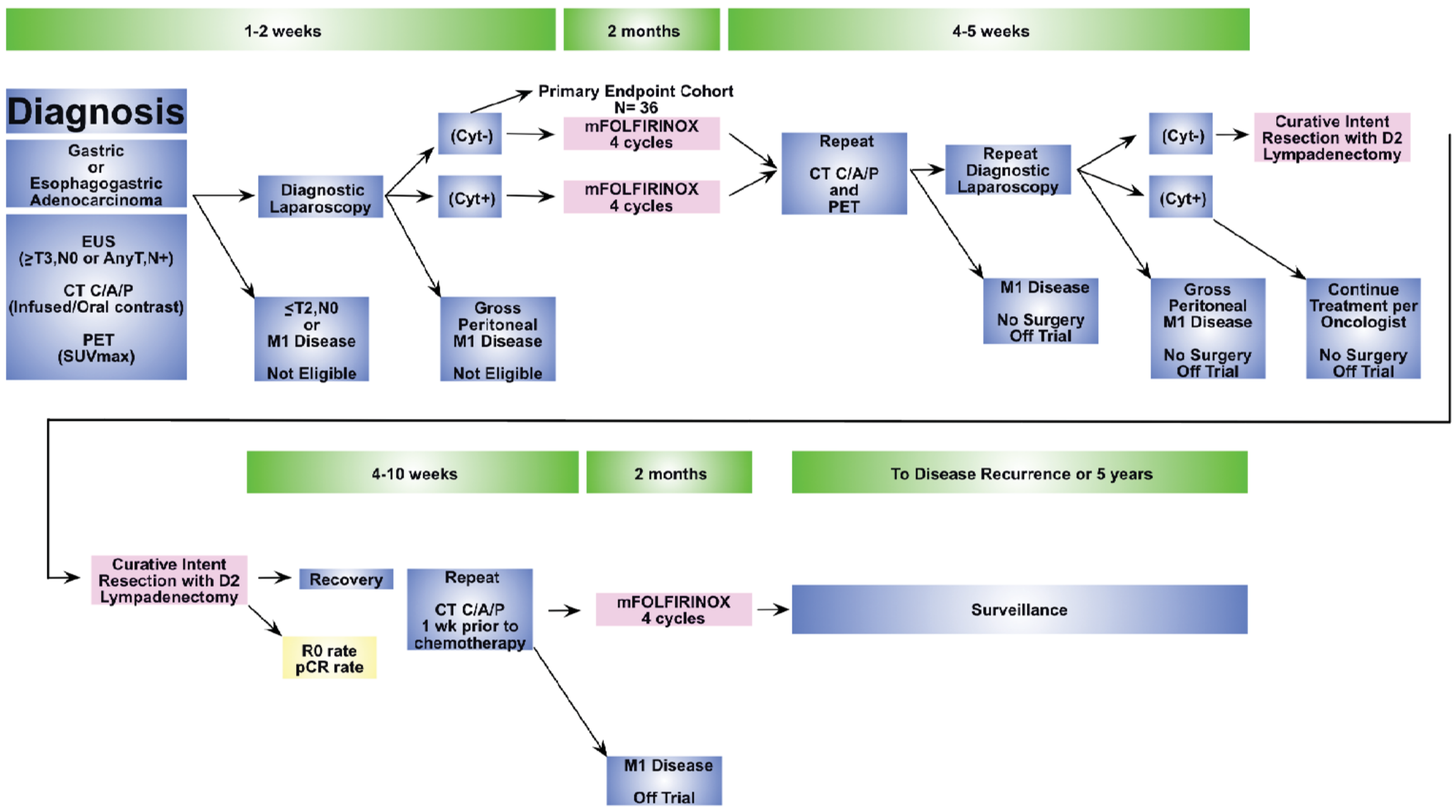

1-2 weeks
2 months
4-5 weeks
Diagnosis
Gastric or Esophagogastric Adenocarcinoma
EUS (≥T3,N0 or AnyT,N+)
CT C/A/P (Infused/Oral contrast)
PET (SUVmax)
Diagnostic Laparoscopy
≤T2,N0 or M1 Disease
Not Eligible
(Cyt-)
(Cyt+)
Gross Peritoneal M1 Disease
Not Eligible
Primary Endpoint Cohort N= 36
mFOLFIRINOX 4 cycles
mFOLFIRINOX 4 cycles
Repeat CT C/A/P and PET
Repeat Diagnostic Laparoscopy
M1 Disease
No Surgery Off Trial
(Cyt-)
(Cyt+)
Curative Intent Resection with D2 Lympadenectomy
Gross Peritoneal M1 Disease
No Surgery Off Trial
Continue Treatment per Oncologist
No Surgery Off Trial
4-10 weeks
2 months
To Disease Recurrence or 5 years
Curative Intent Resection with D2 Lympadenectomy
Recovery
R0 rate pCR rate
Repeat CT C/A/P 1 wk prior to chemotherapy
mFOLFIRINOX 4 cycles
Surveillance
M1 Disease
Off Trial

Schema:

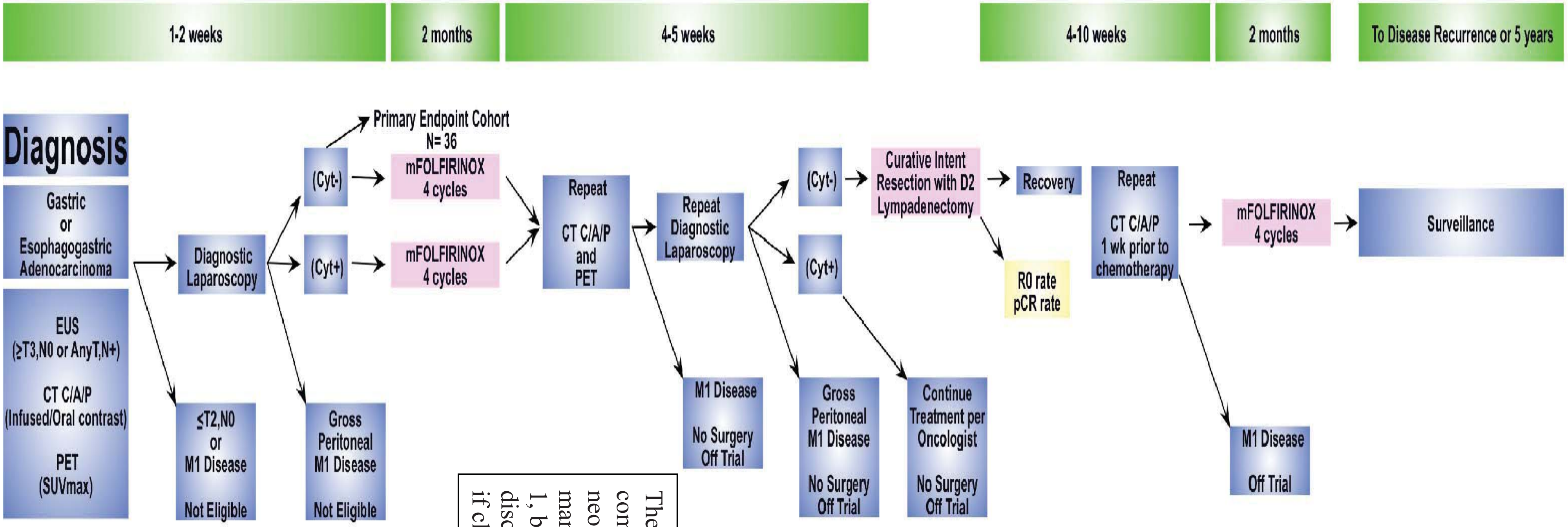

The diagnostic laparoscopy after completion of the 4 cycles of neoadjuvant chemotherapy is not mandated for cohort 2 as in cohort 1, but should be performed at the discretion of the treating physicians if clinically indicated.

TABLE OF CONTENTS

Page

Investigator Listing i
Expansion Cohort 2 Amendment Summary iii
Patient Eligibility iv
Schema (enlargened) v
Schema (full)............ vi

1. INTRODUCTION....................1
1.1 **Locally Advanced Gastroesophageal Adenocarcinoma (GEC)**....................1
1.2 **FOLFIRINOX for metastatic pancreatic cancer**....................5
1.3 **The optimal dose of irinotecan in FOLFIRINOX is unknown**....................5
1.4 **FOLFIRINOX in other gastrointestinal malignancies**....................6
1.5 **Phase I genotyping mFOLFIRINOX amendment**....................7
1.6 **FOLFIRINOX with trastuzumab**....................7
1.7 **FOLFIRINOX Cohort 1 results and rationale for Cohort 2**....................7

2. OBJECTIVES 7
2.1 **Primary objective**....................7
2.2 **Secondary objectives**....................7
2.3 **Exploratory Laboratory Correlatives.**....................8

3. STUDY DESIGN....................8
3.1 **Inclusion criteria** 8
3.2 **Exclusion criteria** 9
3.3 **Drug administration and follow-up**....................10
Trastuzumab (Herceptin) – for HER2+ Patients....................10
3.4 **Dose levels for dose adjustments**....................12
3.5 **Guidelines for treatment delay and dose reductions**…………..…………………12
3.6 **Agent formulation and procurement**....................14

4. MEASUREMENT OF EFFECT 16
4.1 **Measurable disease** 17
4.2 **Non-measurable disease** 17
4.3 **Target lesions**....................17
4.4 **Non-target lesions**....................17
4.5 **Response criteria** 17
4.6 **FDG-PET/CT Imaging Procedures, Interpretation, Analysis**…..………………19
4.7 **Duration of Therapy and Followup**…..……………………………………….22
4.8 **Progression-free survival and Overall Survival**……………………………..…23
4.9 **Criteria for Removal from Study** ……………………….………………..…23

5. MEASUREMENT OF EFFECT (Pathological) 23
5.1 **Complete R0 resection Cohort 1**....................23

5.2 **Pathologic Complete Response (pCR)Cohort 1** 23
5.3 **Pathologic Response Grade 1 Intestinal Histology Cohort 2**................................23

6. GENOTYPING ASSAY 24
6.4 Blood banking...24

7. STATISTICAL CONSIDERATIONS 25
8. STUDY CALENDAR 27

REFERENCES..29

APPENDICES

APPENDIX A: Performance status criteria..……………………….32
APPENDIX B: Record of bowel movements…..…………………………………………..…33
APPENDIX C: CYP3A4 substrates, inhibitors and inducers……………………………...34
APPENDIX D: Genotyping blood sample collection form…………………………………35

1.0 Introduction

1.1 Locally Advanced Gastroesophageal Adenocarcinoma (GEC)

At present, surgery is the sole curative option for operable GEC. For locally advanced ($\geq$T3 and/or N+) non-metastatic disease, the type of surgical approach and extent of surgery depends upon the anatomical location, extent, and TNM stage of the tumor[1, 2]. For the most part, there is consensus regarding surgical approach for Siewert type I (considered esophageal cancers), treated by either an en bloc transthoracic or transhiatal esophagectomy with two-field lymphadenectomy. For type III EGJ tumors, a total gastrectomy via laparotomy and D2 lymphadenectomy without routine distal splenopancreatectomy is recommended. For Siewert type II EGJ tumors, either of these procedures are accepted approaches [3, 4]. Although there is some controversy, proximal stomach tumors (including gastric cardia) require either a total gastrectomy or proximal gastrectomy with resection of 5 to 10 cm of esophagus. Tumors of the middle third/fundus of the stomach usually require a total gastrectomy, however tumors of the distal third of the stomach can undergo radical subtotal (75-85%) gastrectomy [5]. **Most trials have demonstrated that achieving a R0 resection is critical** and is prognostic of improved 5-year survival for both EGJ and GC, in contrast to R1/R2 resection (microscopic/macroscopic tumor) [6]. **Additionally, pathologic complete response (pCR)** after neoadjuvant chemotherapy tends to predict better overall prognosis.[7 8]

With respect to D1 versus D2 lymph node dissection, a recent metaanalysis evaluating 5 RCTs, involving 1642 patients with GC enrolled from 1982 to 2005, revealed a higher operative mortality associated with D2 dissections in the earlier trials, while recent trials have similar rates of mortality between D2 and D1 lymphadenectomy [9]. A trend of improved survival existed among D2 patients who did not undergo resection of the spleen or pancreas, as well as for patients with T3/T4 cancers. Given that D2 resections may improve the accuracy of locoregional staging and may improve survival when avoiding routine distal pancreatectomy and splenectomy, most agree that this modified D2 approach is appropriate and has been accepted as standard of care in the recent NCCN guidelines [10]. The D1 versus D2 variable plays a significant role when comparing results across various trials (reported and ongoing) for locally advanced GEC (**Figure 1**).

Disease recurrence after curative GEC resection alone is unacceptably high, with dismal five year survival rates of less than 40% for all comers undergoing resection, and less than 75% even for early Stage Ib patients [11]. High recurrence rates, coupled with generally late detection due to vague symptoms at earlier stages, indicate that we are most often palliating recurrent and/or metastatic disease.

Histologic classification identifies two major subtypes within GEC - namely, diffuse versus intestinal morphology, with potential prognostic implication [12]. The complexity and heterogeneity of GEC, in terms of patient ethnicity [13], as well as anatomical, histological, and molecular subsets, has resulted in a number of categorizations, perioperative treatment strategies, and surgical approaches [2, 14]. Specifically, the anatomical distinction of 'proximal' (EGJ) versus 'distal' (GC) cancer has led to diverse inclusion/exclusion criteria for clinical trial enrollment, embodying various combinations of chemotherapy and radiation before and/or after surgery (**Figure 1**). Supporting evidence of each of these approaches consequently has led to a number of different practices by geographical region and Institution/Physician, based on varying experience and preference, and discussed in further detail below. It is well known that surgery is the required modality of curative intent treatment of locally advanced GEC. A number of clinical trials have established various perioperative treatment options that further improve mOS compared to surgery alone, including i) neoadjuvant chemoradiation (CRT→**<u>S</u>**), ii) adjuvant CRT (**<u>S</u>**→CRT), iii) neoadjuvant chemotherapy (C→**<u>S</u>**), iv) adjuvant chemotherapy (**<u>S</u>**→C), v) perioperative chemotherapy (sandwich approach) (C→**<u>S</u>**→C), and vi) induction chemotherapy followed by neoadjuvant CRT (C→CRT→**<u>S</u>**).[2]

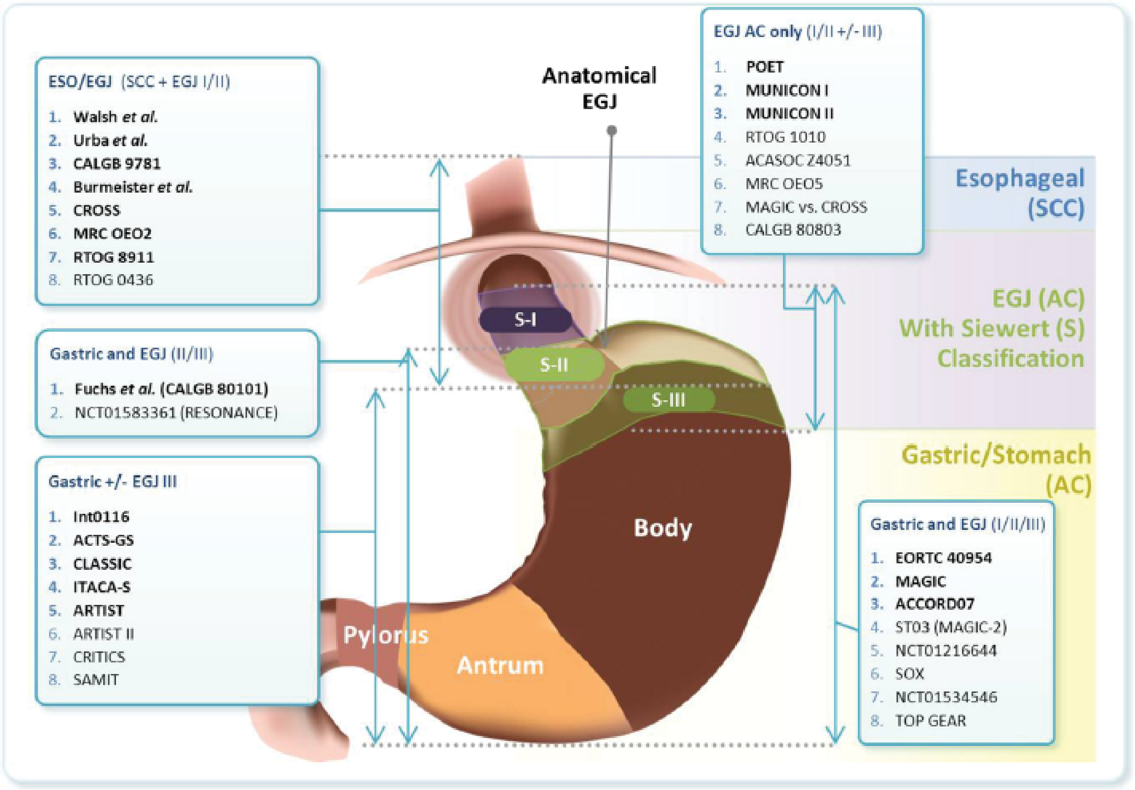

Figure 1 Completed and ongoing perioperative gastroesophageal adenocarcinoma clinical trials, demonstrating enrollment criteria by tumor location. See text and tables for trial references. Abbreviations: SCC, squamous cell carcinoma; EGJ, esophagogastric junction; AC, adenocarcinoma. Bolded: clinical trials with results reported; Not Bolded: clinical trials that are ongoing.

Perioperative Chemotherapy (Sandwich chemotherapy)

With respect to perioperative chemotherapy (C→S→C), large clinical trials (**Figure 1**) have included both gastric adenocarcinoma and/or esophagogastric adenocarcinoma as inclusion criteria, including the MAGIC (ECF x 3 before and after surgery)[15] and ACCORD07 (CF x 3 before and after surgery)[16] trials. These trials demonstrated benefit over surgery alone with HR 0.75 and 0.69, respectively. The survival advantage was similar to that observed with neoadjuvant chemoradiotherapy approaches (CRT→S) for esophagogatric adenocarcinoma with HR 0.74 (CROSS, Carbo/taxol/RT x 5 week then surgery)[17], and similar outcomes to adjuvant chemoradiotherapy (S→CRT) for distal gastric cancer (Int-0116) with HR 0.76. Therefore, any of these strategies are considered options for locally advanced gastric and esophagogastric adenocarcinomas based on physician preference and clinical scenario.[2]

A number of phase II trials have also been reported, and trials are also ongoing that are evaluating other chemotherapy regimens for perioperative chemotherapy C→S→C. Table 1 shows pathologic outcomes of various neoadjuvant chemotherapy regimens with respect to R0 resection rate and pathologic complete response (pCR) rate. Triple drug regimens which include docetaxel/platinum/5FU have increased R0 and pCR rates substantially,[18, 19] compared to platinum/5FU+/-epirubicin regimens seen in MAGIC and ACCORD07. These are now being tested prospectively in larger phase III trials. Regression of the primary tumor is typically documented by the amount of viable tumor versus the amount of fibrosis, ranging from no

evidence of any treatment effect to a complete response with no viable tumor identified, as previously described (ref) [Grade 1a: complete remission (pCR), no residual tumor/tumor bed; Grade 1b: subtotal remission, <10% residual tumor/tumor bed; Grade 2: partial remission, 10-50% residual tumor/tumor bed; Grade 3: minor/no remission, >50% residual tumor/tumor bed].[18] Proximal, distal, and circumferential are assessed to determine the completeness of resection. The absence of tumor cells at the proximal and distal margins are required for an R0 resection.

Table 1: Pathologic outcomes of various neoadjuvant chemotherapy regimens with respect to R0 resection rate and pathologic complete response (pCR) rate.

Chemotherapy Regimen	N	R0 (%)	pCR (%)	pCR 95% CI
No chemo → surgery alone[15, 16]	-	Range 69-74%	-	-
MAGIC [15]<br>**ECF** (epirubicin/cisplatin/5FU)	250	79	4	1.9 – 7.2
ACCORD07[16]<br>**CF** (Cisplatin/5FU)	109	84	3	0.06 - 8.1
**ECX** [20] (epirubicin/cisplatin/capecitabine)	34	81	5.9	0 - 14
**DCF** [21]<br>(docetaxel/cisplatin/5FU)	43	95	9.3	3.3 - 23.1
**DCF**[22]	34	85	11.7	4.1 – 27.2
**DCX**[19]	51	90.2	13.7	6.5 - 26
**FLOT** [18](docetaxel/oxaliplatin/5FU)	46	NR	17.4	6.6 – 34.7
**FLOT-trastuzumab**[46] **(for HER2+)**	45	93.3	22.2	-

Despite the promising increase in pCR and R0 rates seen with docetaxel added to either cisplatin/5FU or oxaliplatin/5FU, there is an increase in neuropathy which is often permanent – all grade neurosensory toxicity up to 40% and grade 3/4 up to 10%.[23, 24] However, we have enrolled several patients with GEC on our phase I genotyping mFOLFIRINOX trial (NCT01643499) for advanced GI malignancies with extraordinary response rates (75%). An example of a case is demonstrated in **Figure 2**; this patient was initiated at 135mg/m2 due to UGT1A1 genotype (*1/*28). Despite this dose reduction, the patient had a dramatic response both clinically and radiolocally. Therefore, the rationale of evaluating mFOLFIRINOX in the perioperative setting of

this disease is to attempt to achieve similar R0 and pCR rates as seen with triple drug

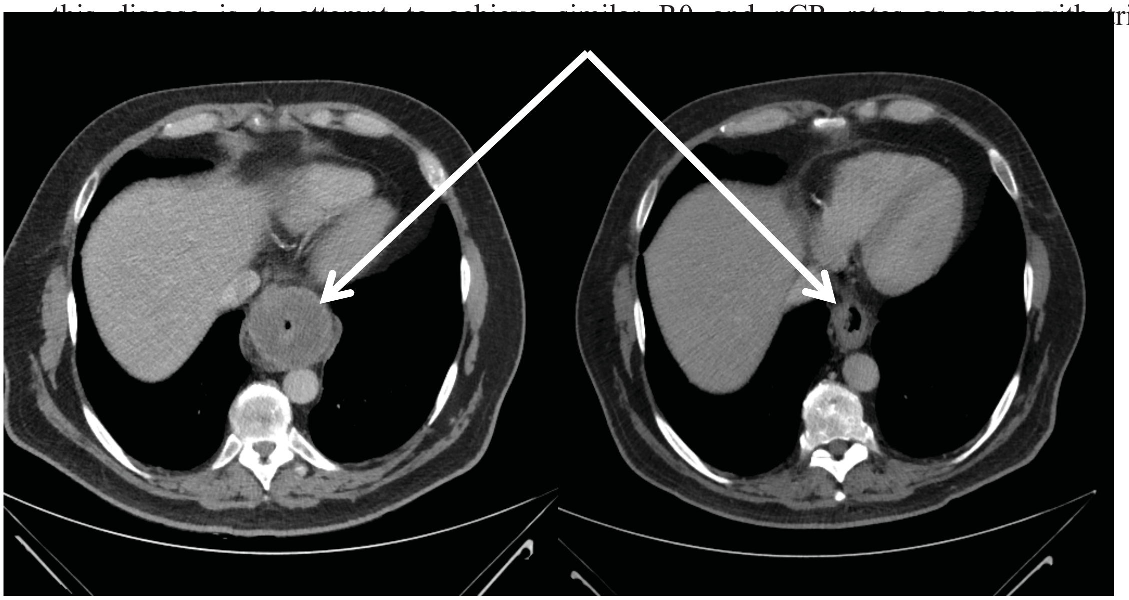

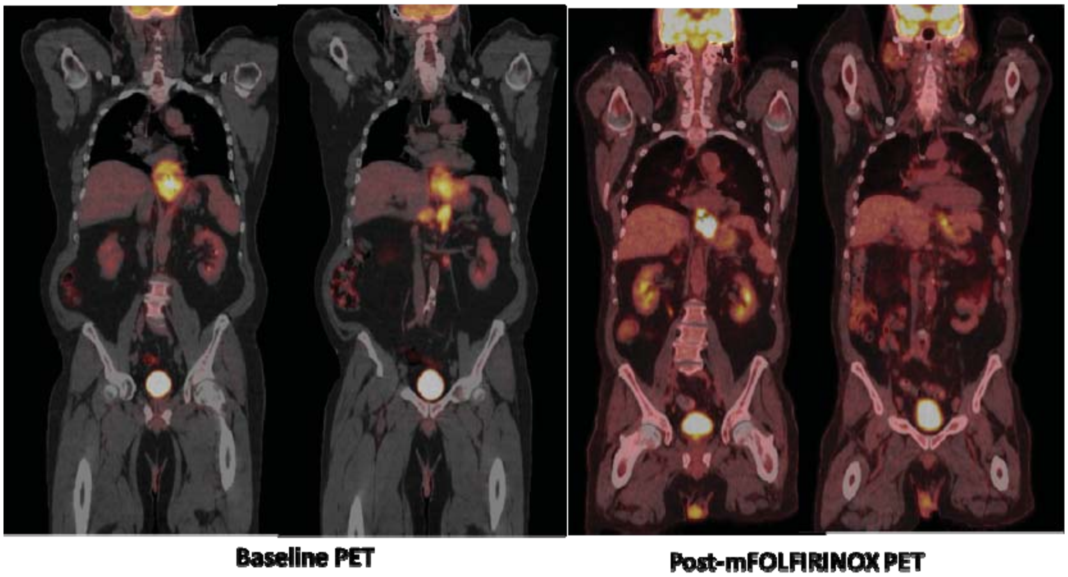

Figure 2: Above: CT scan and Below: PET scan; before and after mFOLFIRINOX therapy (dose reduced to 135mg/m2 based on UG1A1 genotype at baseline on phase I pilot trial) in a gastroesophageal junction patient with retroperitoneal disease.

Evaluation of Treatment Response Using Non-invasive Metabolic Imaging[25-29]
A recent advance in evaluating tumor response is the use of metabolic imaging. The most common approach is to detect tumor cell glycolytic activity by observing uptake of a radiolabeled metabolite, FDG, which can be detected by PET imaging. Several studies have demonstrated the ability of FDG-PET to assess response to preoperative chemotherapy with 5-FU and cisplatin, or chemoradiotherapy in patients with esophageal cancer. Sensitivity to detect response was between 78% and 100% and corresponding specificity was 55% to 95%. In two single-institution studies, metabolic non-responders, defined as having less than a 35% decrease in SUVmax from baseline, who were identified 14 days after initiation of therapy had reduced overall survival rates. This finding was validated in a larger, multicenter Phase II trial that demonstrated that early metabolic

responders to 5-FU and cisplatin-based chemotherapy had significantly better event-free survival compared with metabolic non-responders (29.7 months vs 14.1 months). This study also suggests a benefit of early identification of metabolic non-responders, as these patients were treated with immediate surgical resection. Compared to patients from a prior study, where metabolic non-responders continued with chemotherapy, nonresponders who went directly to surgery had improved recurrence-free periods and median survival times compared to the same group of patients who continued a total of 3 months of likely ineffective preoperative chemotherapy (2-year overall survival, 37% vs 26%). Thus, PET-directed treatment may help to tailor multimodality therapy based on early identification of patients who might benefit from either earlier referral for surgery, or potentially changing the treatment regimen in the absence of response. Change in PET SUV will be assessed pre/post four cycles of neoadjuvant mFOLFIRINOX to describe the rate of PET response compared to these historical controls in an exploratory correlative study.

1.2 FOLFIRINOX for metastatic pancreatic cancer

Background of the regimen FOLFIRINOX: In an attempt to improve on outcomes with gemcitabine, Conroy and colleagues conducted a randomized phase III trial of FOLFIRINOX (bolus 5-FU at 400 mg/m^2, infusional 5-FU at 2,400 mg/m^2 over 46 hours, leucovorin at 400 mg/m^2, irinotecan at 180 mg/m^2, and oxaliplatin at 85 mg/m^2 given once every 14 days) vs. gemcitabine as first-line treatment of metastatic pancreatic cancer.[30] Notable inclusion criteria were an ECOG performance status of 0 or 1 and a total bilirubin < 1.5 times the upper limit of normal. Median overall survival was 11.1 months in the FOLFIRINOX arm, compared to 6.8 months in the gemcitabine arm (p<0.0001 by the log-rank test). This remarkable improvement in overall survival came at the cost of increased toxicity, most notably Grade 3/4 neutropenia in 45.7% of patients (compared to 18.7% with gemcitabine) and febrile neutropenia in 5.4% of patients (compared to 0.6% with gemcitabine). This difference was observed despite the fact that 45.2% of patients in the FOLFIRINOX were treated with pegfilgrastim (Neulasta), compared to 5.3% in the gemcitabine arm. FOLFIRINOX also led to more Grade 3/4 non-hematologic toxicities, including neuropathy (9% vs. 0%), vomiting (14.5% vs. 4.7%), fatigue (23.2% vs. 14.2%) and diarrhea (12.7% vs. 1.2%) but overall quality of life was improved.[31]

Additional trials are underway with modified FOLFIRINOX (mFOLFIRINOX), in which the bolus 5-FU has been eliminated to reduce. Conroy and colleagues are conducting a randomized phase III trial that is comparing mFOLFIRINOX to gemcitabine as adjuvant therapy for patients with resected pancreatic cancer.

1.3 The optimal dose of irinotecan in FOLFIRINOX is unknown

Irinotecan {7-ethyl-10-[4-(1-piperidino)-1-piperidino]carbonyloxycamptothecin}[24] is a topoisomerase I inhibitor that has activity in a variety of gastrointestinal malignancies, and is approved by the U.S. Food and Drug Administration for use in metastatic colorectal cancer. The active metabolite of irinotecan, SN-38, is glucuronidated by the enzyme UDP-glucuronosyltransferase 1 family polypeptide A1, which is encoded by the UGT1A1 gene. The UGT1A1 gene has germline polymorphisms related to the number of TA repeats in the promoter region. The wild-type allele (UGT1A1*1) has six TA repeats, while the most common variant allele (UGT1A1*28) has seven TA repeats. Patients with the UGT1A1*28 polymorphism have decreased enzyme activity, resulting in decreased clearance of SN-38. A number of pharmacogenetic studies have demonstrated that patients with the UGT1A1*28 polymorphism are at higher risk for severe neutropenia related to irinotecan,[32-35] and the product label in the United

States was revised in 2005 to include this information and recommend that patients with the UGT1A1*28/*28 genotype receive a lower starting dose of irinotecan. A systematic review concluded that these patients have a dose-dependent increased risk of hematologic toxicity,[36] while another concluded that these patients have a dose-dependent increased risk of severe diarrhea.[37]

Since the FOLFIRINOX regimen in the randomized phase III trial of FOLFIRINOX vs. gemcitabine utilized an irinotecan dose of 180 mg/m^2 (the standard dose in FOLFIRI) for all patients, the increased toxicity (in particular, Grade 3/4 neutropenia and Grade 3/4 diarrhea) may have been a result of this dose being too high for patients with the UGT1A1*1/*28 and UGT1A1*28/*28 genotypes. FOLFIRINOX without 5FU bolus and with UGT1A1 genotype-directed dosing of irinotecan is referred to as gFOLFIRINOX.

1.4 FOLFIRINOX in other gastrointestinal malignancies

In addition to metastatic pancreatic cancer, FOLFIRINOX has been demonstrated to have synergistic activity, and is a reasonable treatment option for some patients with other gastrointestinal malignancies. FOLFOXIRI, which involves a higher dose of infusional 5-FU (3,200 mg/m^2 over 48 hours) and a lower dose of irinotecan (165 mg/m^2) compared to mFOLFIRINOX, has been studied extensively in metastatic colorectal cancer. In a randomized phase III trial comparing FOLFOXIRI and FOLFIRI as first-line therapy for metastatic colorectal cancer, FOLFOXIRI significantly improved response rate, progression-free survival and overall survival, albeit with increased toxicity.[38] Although FOLFOXIRI has not become a widely accepted standard of care in the United States due to concerns about toxicity, mFOLFIRINOX would be a reasonable consideration in certain circumstances, such as when rapid tumor shrinkage is desired prior to attempted surgical resection with curative intent. FOLFOXIRI has also been studied in a single arm phase II trial for patients with metastatic gastric cancer, with promising response rates but increased toxicity compared with historical controls.[39] Single agent irinotecan and the combination of fluoropyrimidines and oxaliplatin have anti-tumor activity in advanced biliary cancers (gall bladder cancer, cholangiocarcinoma, ampullary cancer). In a phase I trial of FOLFIRINOX in advanced solid tumors, two out of three patients with cholangiocarcinoma had an objective response to therapy.[40] While the combination of gemcitabine and cisplatin is standard of care for first-line therapy of advanced biliary cancers, mFOLFIRINOX would be a reasonable alternative for patients with renal insufficiency. Additionally, mFOLFIRINOX would be a reasonable treatment option for patients with adenocarcinoma of unclear primary in whom a gastrointestinal primary is most likely.

Most importantly, given the high response rates and clinical benefit observed in GEC patients treated at our center (see section 1.5), mFOLFIRINOX is a reasonable treatment options for these patients in the metastatic setting, but also in the perioperative setting to improve R0 and pCR rates and potentially inhibit micrometastatic disease, with the intention of decreasing the risk of disease recurrence, both locally and distantly, in order to improve overall survival. The benefit of only one neurotoxic chemotherapy (oxaliplatin) without taxane may limit this debilitating toxicity observed frequently in taxane/platinum/5FU regimens.

1.5 Phase I gFOLFIRINOX amendment for advanced metastatic GI malignancies

As of January 1, 2014, we have enrolled a total of 41 subjects on this study (NCT01643499): 15 in the *1/*1 cohort; 16 in the *1/*28 cohort; and 10 in the *28/*28 cohort. We have observed 2 DLTs in the *1/*1 cohort (both neutropenic fevers); 2 DLTs in the *1/*28 cohort (one with Grade

3 fatigue and diarrhea; one with Grade 3 fatigue); and 3 DLTs in the *28/*28 cohort (two neutropenic fevers; one Grade 3 abdominal pain). Tumor types have included the following: 20 pancreatic, 11 biliary tract, 7 GEC, and 3 adenocarcinoma of unclear GI primary. Data regarding clinical outcomes are not yet mature, but partial responses to therapy by RECIST (version 1.1)[11] have been observed in all tumor types (75% of GEC cases). We have concluded that the *1/*28 dose is tolerable, while the *28/*28 dose is not. We hypothesize that routine use of prophylactic pegfilgrastim will likely make the *1/*1 and *28/*28 doses even more tolerable (since neutropenic fever was the most common DLT in these cohorts).

In order to gather additional information about the efficacy of genotype-guided dosing of gFOLFIRINOX in specific tumor types (a secondary objective of the study), we amended the phase I study to enroll additional subjects with pancreatic and biliary tract cancers for the metastatatic setting (but not metastatic GEC due to competing studies). However, herein, this separate protocol of perioperative gFOLFIRINOX is specific to GEC that is locally advanced as determined by EUS (stage ≥T3 and/or any N+ disease) without metastatic disease and eligible for curative intent resection.

1.6 FOLFIRINOX with trastuzumab

HER2+ gastric and esophageal adenocarcinoma occurs in approximately 10-15% of patients. In the metastatic setting, the addition of trastuzumab (Herceptin) to chemotherapy improved response rate, mPFS, and mOS. HER2+ breast cancer derives survival benefit from addition of anti-HER2 agents in the curative intent setting (neoadjuvant and/or adjuvant treatment). Phase II trials have included trastuzumab therapy in the curative intent setting with chemoradiotherapy in an ogoing RTOG 1010 trial (NCT01196390) and also trastuzumab has been safely added to to perioperative chemotherapy (FLOT – 5FU, leucovorin, oxaliplatin, and docetaxel – or 'HER-FLOT')[46] with very promising results. Finally, we reported a phase I pilot study of gFOLFIRINOX with trastuzumab for the subset of patients with HER2+ cancer. The gFOLFIRINOX-T was safe and response rates were 75% with 25% of patients obtaining a CR.[47] We therefore believe there is rationale to test gFOLFIRINOX-T in the perioperative setting in this trial for those patients deemed HER2+ by routine companion diagnostic tests.

1.7 g**FOLFIRINOX Cohort 1 results and rationale for Cohort 2**

In cohort 1, 36 patients were enrolled towards the intention to treat (ITT) population (gastric body/esophagogastric cancer) between 2/2014-8/2018; 75% of patients were male, with median age 66 (range 27-85). All patients completed all 4 cycles of neoadjuvant genotype directed FOLFIRINOX (gFOLFIRINOX) therapy: 10% had any dose reduction of irinotecan (16%/0%/25% by genotype 6/6, 6/7, 7/7); any Grade 3/4 toxicity occurred in 35% of pts (32% 6/6, 29% 6/7, 75% 7/7). Grade 3/4 toxicity in >5% of patients were as follows: diarrhea (17.5%; 6/6 21%, 6/7 11%, 7/7 25%), anemia (5%), vomiting (5%). The primary efficacy endpoint of R0 resection was met. Of paients going to surgery, both R1 resections were Gastric Body linitus plastica diffuse type patients. Pathologic response grade (PRG) of 1(a complete + b minimal residual <10%) was achieved in 36% of ITT patients, and a remarkable 46% of intestinal type histology. Given these findings, we will further study in a prospective manner the Grade 1 PRG rate in an expansion cohort 2, specifically in the intestinal subgroup. The Grade 1 response was low in diffuse type cancers and we will assess other treatments for this subgroup of patients. Given that some patients with mixed type tumors experience grade 1 response, we will allow these patients to enroll for explorative endpoints but not towards the ITT analysis for cohort 2 (similar to antral/pylorus and cytology positive tumors as in cohort, which will continue for cohort 2).

2.0 Objectives

The rationale of gFOLFIRINOX (and trastuzumab for HER2+) for perioperative GEC is to attain higher pCR and R0 resection rates than observed in the previous clinical trials including MAGIC (ECF) and ACCORD07 (CF) while limiting the toxicity, particularly neurotoxicity, observed in recent reports with various docetaxel/platinum/5FU combinations.[23]

2.1 Primary objectives

Co-primary endpoints are to determine the i) **R0 resection rate** and ii) **pCR rate** of up to 36 patients treated with 4 cycles of neoadjuvant gFOLFIRINOX (and trastuzumab for HER2+ GEC) (UGTA1A1 genotype-dosed irinotecan) regimen.

Regression of the primary tumor will be documented by the amount of viable tumor versus the amount of fibrosis, ranging from no evidence of any treatment effect to a complete response with no viable tumor identified, as previously described (ref) [Grade 1a: complete remission (pCR), no residual tumor/tumor bed; Grade 1b: subtotal remission, <10% residual tumor/tumor bed; Grade 2: partial remission, 10-50% residual tumor/tumor bed; Grade 3: minor/no remission, >50% residual tumor/tumor bed]. Proximal, distal, and circumferential margins will be assessed to determine the completeness of resection. The absence of tumor cells at the proximal and distal margins will be required to be classified as an R0 resection.

2.2 Secondary objectives

i) **Response Rate** (radiographic (CT), and metabolic (PET SUVmax)) to chemotherapy.
ii) **Chemotherapy-related toxicity**.
iii) **Surgical morbidity**. Postoperative morbidity will be classified according to standard scales.
iv) **Overall survival** (OS) measured from the time of histologic diagnosis.
v) **Disease-free survival** measured from the time of histologic diagnosis.
vi) **Pattern of recurrence** (distant, locoregional, both).
vii) **HER2+ vs HER2- difference in clinical outcomes**

2.3

3.0 Study design

3.1 Inclusion criteria

1. Histologically confirmed locally advanced gastric (primary endpoint includes proximal and mid-body stomach) or esophagogastric adenocarcinoma. Distal gastric (antral) adenocarcinomas are eligible for enrolment but will not be included in the primary analysis.

2. Locally advanced disease as determined by EUS stage ≥T3 and/or any T,N+ disease without metastatic disease (Mx).

3. HER2+ and HER2- patients are eligible

4. Cardiac Ejection Fraction ≥ 50% (for HER2+ patients) as assessed by echocardiogram, MUGA scan, or cardiac MRI

5. All patients must have diagnostic laparoscopy with diagnostic washings for cytology. Both Cytology positive and negative patients are eligible for enrolment, but only cytology negative patients will be included in the primary analyses. Gross peritoneal disease is not eligible.

6. Age ≥18 years.

7. Eastern Cooperative Oncology Group (ECOG) performance status ≤ 1 (see Appendix A).

8. Eligible for surgery with curative intent.

9. Adequate organ function, as defined by each of the following:
   Absolute neutrophil count (ANC) ≥ l250/µl
   Hemoglobin ≥ 9g/dL
   Platelets ≥ 100,000/ µl)
   Total bilirubin < 1.5 x upper limit of normal
   SGOT and SGPT < 2.5 x upper limit of normal
   Creatinine ≤ 1.5 x upper limit of normal

10. Measurable or non-measurable disease by RECIST 1.1 will be allowed.

11. Women of child-bearing potential and men must agree to use adequate contraception (hormonal or barrier method of birth control; abstinence) prior to study entry and for the duration of study participation, up until 30 days after final study treatment. Should a woman become pregnant or suspect that she is pregnant while participating in this study, she should inform her treating physician immediately.

12. Patients taking substrates, inhibitors, or inducers (see Appendix C) of CYP3A4 should be encouraged to switch to alternative drugs whenever possible, given the potential for drug-drug interactions with irinotecan.

13. Signed informed consent.

3.2 Exclusion criteria

1. Previous or concurrent malignancy, except for adequately treated basal cell or squamous cell skin cancer, in situ cervical cancer, or any other cancer for which the patient has been previously treated and the lifetime recurrence risk is less than 30%.

2. Inflammatory bowel disease that is uncontrolled or on active treatment (Crohn's disease, ulcerative colitis).

3. Diarrhea, grade 1 or greater by the National Cancer Institute Common Terminology Criteria for Adverse Events (NCI-CTCAE, v. 4.0).

4. Neuropathy, grade 2 or greater by NCI-CTCAE, v. 4.0.

5. Serious underlying medical or psychiatric illnesses that would, in the opinion of the treating physician, substantially increase the risk for complications related to treatment.

6. Active uncontrolled bleeding.
7. Pregnancy or breastfeeding.
8. Major surgery within 4 weeks.

3.3 Drug administration

gFOLFIRINOX (and addition of trastuzumab for HER2+ GEC: mFOLFIRINOX-T) will be given once every 14 days (2 weeks) which equals 1 cycle.

Preoperative Therapy:
After completion of staging studies (CT, PET, EUS, Laparoscopy), pre-operative (neoadjuvant) gFOLFIRINOX (gFOLFIRINOX-T for HER2+) treatment will be given for 4 cycles (2 months) as tolerated. Patients will then have repeat CT and PET restaging upon completion of the neoadjuvant therapy phase.

Surgery:
If CT and PET continue to demonstrate no evidence of metastatic disease, patients will then return to have a repeat EUS (optional – if clinically indicated) for restaging (patients will be encouraged to participate in the separate protocol under IRB 16294A for CTC/ctDNA analysis within portal vein blood obtained via EUS) and repeat diagnostic laparoscopy (OPTIONAL for COHORT 2, as clinically indicated but not mandated) to confirm no peritoneal dissemination (previously cytology positive patients should have repeat laparoscopy and if they convert to cytology negative, they may be considered for surgical resection). Eligible patients will then go on to surgery per routine clinical care.

Post-operative Therapy:
Post-operative (adjuvant) gFOLFIRINOX (gFOLFIRINOX-T for HER2+) may resume between 5-10 weeks post surgery, as tolerated. A new baseline CT will be obtained within 2 weeks of resuming therapy to confirm no evidence of recurrent metastatic disease developed since CT prior to surgery. Therapy will consist of 4 more cycles (2 months) of therapy as tolerated.

**If there is disease progression or there is definitively lack of response to therapy with advanced ypTN pathology, and the utility of continued gFOLFIRINOX in the adjuvant setting after surgery is questionable, per treating physician discretion, the patient may be treated with standard FLOT therapy for 4 cycles off-protocol (standard perioperative therapy per ASCO 2017 annual meeting presentation of the phase III study with 5FU, leucovorin, oxaliplatin, docetaxel, Al Batran et al. J Clin Oncol 35, 2017 (suppl; abstr 4004)).[48] This decision should be discussed with the principle investigator Dr. Catenacci prior to proceding. Patients will complete 4 cycles as tolerated, then surveillance will continue per protocol and patients will still be followed for secondary endpoints DFS and OS. It should be noted that this change in adjuvant therapy does not effect the co-primary endpoints of pathologic CR and R0 complete resection, which have already been obtained prior to this decision point.

Trastuzumab (Herceptin) – for HER2+ Patients

- Trastuzumab will be administered intravenously on Day 1 of each treatment cycle, using an initial dose of 6 mg/kg for Cycle 1, followed by doses of 4 mg/kg Q2W for subsequent treatment cycles.

- Trastuzumab will be given along with gFOLFIRINOX (gFOLFIRINOX-T) for the same planned dosing (4 cycles before and 4 cycles after surgery), as tolerated. disease progression in the first line (PD1) (mFOLFOX6+trastuzumab) or the development of unacceptable toxicity attributed to trastuzumab, or the patient is withdrawn from study treatment for another reason.

- The initial dose of Trastuzumab (Cycle 1, Day 1) will be administered, per standard care, over 90 (± 10) minutes, after the patient will be observed for infusion associated reactions (IARs) such as fever, chills, headache, pruritus, nausea or vomiting, changes in vital signs, etc. If such symptoms occur, slowing or interruption of the infusion may be helpful, and the infusion can be resumed when symptoms abate. If the initial infusion is well tolerated, subsequent infusions may be administered over 30 (± 10) minutes, followed by an observation period of 30 minutes.

- Baseline Cardiac imaging (MUGA or 2-D ECHO) must be performed before and after the neoadjuvant phase (cycles gFOLFIRINOX-T, and before and after the adjuvant phase (4 cycles of gFOLFIRINOX-T.

- Patients who experience infusion-associated symptoms may be premedicated for subsequent infusions using acetaminophen/paracetamol and antihistamines. Dose reduction of Trastuzumab for toxicity is not permitted. Dose delays are permitted for toxicity, including cardiotoxicity documented by a symptomatic or an asymptomatic decrease in LVEF.

- If the patient misses a dose of Trastuzumab for any cycle (i.e., the two sequential administration times are 4 weeks or more apart), a reloading dose of 6 mg/kg of Trastuzumab should be given. If reloading is required for a given cycle, the study therapies should be given at the same schedule. Subsequent maintenance Trastuzumab doses of 4 mg/kg will then be given Q2W, starting 2 weeks later.

<u>Follow up:</u>
Upon completion of adjuvant chemotherapy patients will be followed every 3 months with **CEA and Ca 19-9 serum makers and with clinical exam** for the first 3 years after completion of adjuvant chemotherapy. Follow-up with tumor markers will then be every 6 months in the fourth and fifth years after completion of adjuvant chemotherapy. After five years, yearly MD visits and tumor markers will be performed. Restaging with a **CT chest/abdomen/pelvis infused** will be performed every 6 months or earlier as clinically indicated, after completion of adjuvant chemotherapy, for the first 3 years. Imaging will then be annually for the fourth and fifth year after completion of adjuvant chemotherapy. No routine imaging will be done after five years from completing adjuvant chemotherapy (performed only if clinically indicated). **Upper endoscopy** will be performed at 6-12 months post-surgery and then yearly thereafter to evaluate for local recurrence up to 5 years post-surgery.

Drugs should be administered according to the following dose and schedule.

Table 2. Dose and schedule of gFOLFIRINOX administration

Order of Administration	Drug	Dose (mg/m^2)	Infusion Time (hours)	Infusion Method
1st	Oxaliplatin	85	2	Bolus
2nd	Irinotecan	see Table 3	1.5	Bolus
3rd	5-FU	2400	46	Continuous infusion
3rd	Leucovorin	400	46	Continuous infusion

Table 3. Starting dose of irinotecan by genotype group

UGT1A1 genotype group	Dose of irinotecan
*1/*1 Low risk	180 mg/m^2
*1/*28 Intermediate risk	135 mg/m^2
*28/*28 High Risk	90 mg/m^2

All patients will receive prophylactic treatment with pegfilgrastim (Neulasta) at a dose of 6 mg SQ on day 3 of each 2-week cycle, approximately 12-24 hours after 5-FU pump disconnect. The treating physician may elect to omit pegfilgrastim after the first cycle if it is felt to be unnecessary.

Patients will be premedicated with dexamethasone by mouth or intravenously, ondansetron by mouth or intravenously, and aprepitant 125 mg by mouth. Atropine 0.5 mg subcutaneously will be given as needed for crampy abdominal pain and/or diarrhea immediately after chemotherapy. Patients will be given prescriptions for ondansetron (Zofran; or an alternative 5HT3 receptor antagonist), dexamethasone (Decadron), and aprepitant (Emend) to take on a scheduled basis for nausea and vomiting. Additional as needed medications for nausea and vomiting can be given at the discretion of the treating physician. Diarrhea will be promptly treated with loperamide 4 mg, followed by another 2-4 mg every 3 hours until the patient has no further diarrhea for at least 12 hours. Appendix B should be used by the patient to record bowel movements accurately.

Dose Delays or Discontinuations due to Cardiac Events for HER2+ patients on gFOLFIRNOX-T (gFOLFINOX with trastuzumab)
In this study, all **HER2+** patients must have a baseline LVEF value ≥ 50%, and LVEF is to be monitored as above in section 3.3 during trastuzumab antibody treatment. To ensure patient safety, if an investigator assesses that an AE may be related to cardiac dysfunction, an additional LVEF measurement should be performed, as well as other appropriate procedures such as chest X-ray, and the scheduled cardiac toxicity assessments will continue unchanged.

If **symptomatic** LVSD (CHF) is confirmed by a cardiologist's evaluation in any patient, Trastuzumab should be permanently discontinued, and the patient will continue cytotoxic chemotherapy (and other biologic therapy as appropriate) as tolerated, and followed per protocol. Symptomatic LVSD should be treated and monitored according to standard medical practice.

At the present time, there are inadequate data available to assess the prognostic significance of clinically **asymptomatic** decreases in LVEF values. However, **if a patient's LVEF value decreases to < 50% and with an LVEF decrease of ≥ 10 points from baseline in the absence of symptoms, treatment with Trastuzumab should be withheld temporarily and the LVEF measurement repeated in 4 weeks.** If decreased LVEF is confirmed, Trastuzumab should be permanently discontinued and the patient may be treated with cytotoxic therapy as tolerated, per protocol (**see figure below**).

LVEF, the algorithm shown in the Figure below should be followed to assess the LVEF decrease and determine whether to stop or continue study treatment (with both antibodies and chemotherapy, if the patient is still receiving chemotherapy).

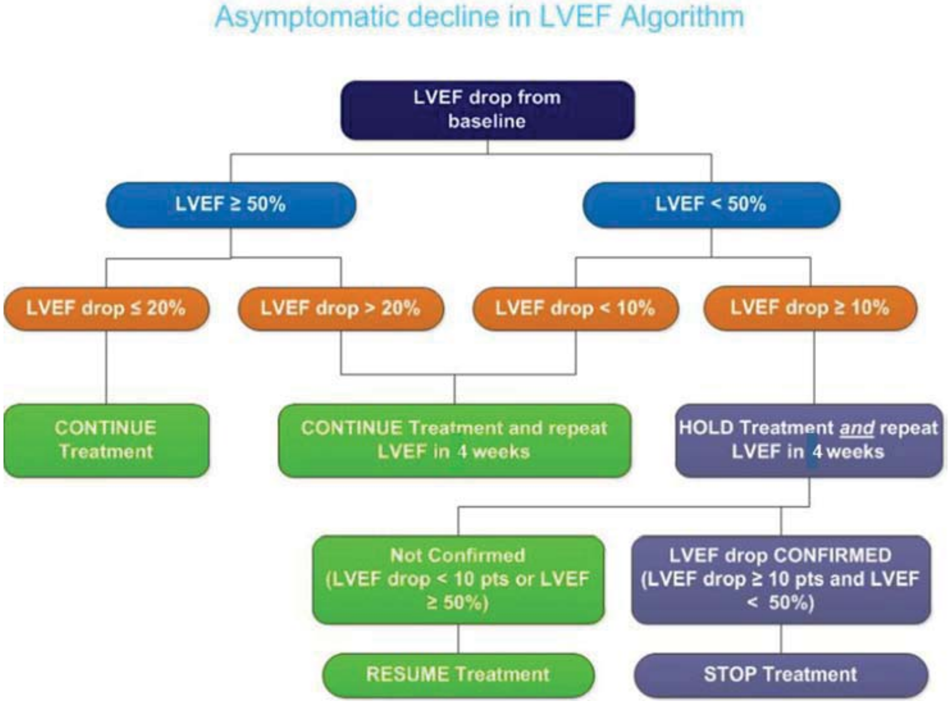

3.4 Dose levels for dose adjustments

Table 4. Dose levels for irinotecan by UGT1A1 genotype group

A. UGT1A1 *1/*1 genotype group

Dose level	Irinotecan dose
0	180 mg/ m^2
-1	135 mg/m^2
-2	90 mg/m^2
-3	45 mg/m2

B. UGT1A1 *1/*28 genotype group

Dose level	Irinotecan dose
0	135 mg/m^2
-1	90 mg/m^2
-2	45 mg/m^2

C. UGT1A1 *28/*28 genotype group

Dose level	Irinotecan dose
0	90 mg/m^2
-1	45 mg/m^2
-2	30 mg/m^2

Table 5. Dose levels for drugs other than irinotecan

Dose level	5-FU infusion (mg/m^2 x 46 hours)	Leucovorin (mg/m^2)	Oxaliplatin (mg/m^2)
0	2400	400	85
-1	1920	400	65
-2	1600	400	50

3.5 Guidelines for treatment delay and dose reductions

Each dose of gFOLFIRINOX will not begin until ANC>1,250/µL, platelets > 100,000/µL, and any treatment-related toxicity is resolved to ≤ grade 1 or baseline. Failure to recover despite a 14 day (two week) delay in therapy will result in dropping irinotecan completely and continuing with FOLFOX therapy alone as tolerated. Once patients have been dose reduced, they cannot be dose re-escalated. Patients whose doses have been reduced to the lowest level of any drug and require further dose reduction will continue with the remaining drugs per protocol.

Table 6. Dose reductions for toxicity at any time during the previous cycle

**Toxicity**	**Dose adjustment of 5-FU, oxaliplatin, irinotecan**
**NEUTROPENIA**	
Grade 2<br>(ANC < 1,500/µL)	With first episode: if resolves to ≤ grade 1 within 7 days, continue all drugs at same dose. If resolution to ≤ grade 1 takes longer than 7 days, reduce all drugs by one dose level.<br><br>With second (and subsequent) episodes: reduce irinotecan by one dose level.
Grade 3/4<br>(ANC < 1,000/µL)	Reduce irinotecan by one dose level.
Febrile neutropenia	Reduce irinotecan by one dose level.
**THROMBOCYTOPENIA**	
Grade 2<br>(platelets < 75,000/µL)	With first episode: if resolves to ≤ grade 1 within 7 days, continue all drugs at same dose. If resolution to ≤ grade 1 takes longer than 7 days, reduce all drugs by one dose level.<br><br>With second (and subsequent) episodes: reduce irinotecan by one dose level.
Grade 3/4<br>(platelets < 50,000/µL)	Reduce irinotecan by one dose level.
**DIARRHEA (despite optimal medical management)**	
Grade 2	First occurrence: continue all drugs at same dose.<br>Second occurrence: reduce irinotecan by one dose level.<br>Third occurrence: reduce irinotecan by one dose level.<br>Subsequent occurrences: reduce irinotecan by one dose level, then per physician discretion with FOLFOX.

Grade 3	First occurrence: reduce irinotecan by one dose level.<br>Second occurrence: reduce irinotecan by one dose level.<br>Subsequent occurrences: reduce irinotecan by one dose level, then per physician discretion with FOLFOX.
Grade 4	Remove patient from protocol therapy.
**NAUSEA AND VOMITING (despite optimal medical management)**	
Grade 3	Reduce oxaliplatin by one dose level.
Grade 4	Remove patient from protocol therapy.
**MUCOSITIS (despite optimal medical management)**	
Grade 3	Reduce 5-FU by one dose level.
Grade 4	Remove patient from protocol therapy.
**NEUROTOXICITY**	
Grade 2 neurotoxicity, persisting between treatments	Reduce oxaliplatin by one dose level.
Grade 3, resolving to ≤ grade 2 between treatments	Reduce oxaliplatin by one dose level.
Grade 3 persisting between treatments, or grade 4	Discontinue oxaliplatin; patient may remain on protocol therapy with other treatment components.
**HEPATIC TOXICITY (first evaluate for biliary obstruction)**	
Grade 3 AST or ALT<br>OR<br>Grade 2/3 total bilirubin	First occurrence: continue all drugs at same dose.<br>Second and subsequent occurrences: reduce all drugs by one dose level.
Grade 4 (AST, ALT, or total bilirubin)	Remove patient from protocol therapy.
**OTHER DRUG-RELATED TOXICITIES**	
Grade 3	Reduce all drugs by one dose level.
Grade 4	Remove patient from protocol therapy.

3.6 Agent formulation and procurement

3.7.1 Irinotecan (NSC# 616348)

Chemical name: (4S)-4,11-diethyl-4-hydroxy-9-[(4-piperidino-piperidino)carbonyloxy]-1H-pyrano[3',4':6,7]indolizino[1,2-b]quinoline-3,14(4H,12H)dione hydrochloride trihydrate

Other names: Camptosar, CPT-11

Molecular formula: $C_{33}H_{38}N_4O_6$•HCl•$3H_2O$

Molecular weight: 677.19 grams/mole

Mode of action: Topoisomerase I inhibitor

Description: Camptothecin derivative

Interactions

Inhibitors of the cytochrome P450 isoenzyme CYP3A4, such as ketoconazole and diltiazem, may increase exposure to irinotecan and its active metabolite SN-38. Conversely, inducers of this isoenzyme may reduce exposure to irinotecan and SN-38. Known inducers of CYP3A4 include phenytoin, phenobarbital, carbamazepine, rifampin, rifabutin, and St. John's wort. Please see Appendix C for a list of CYP3A4 inducers and inhibitors.

Pharmacokinetics

After intravenous infusion of irinotecan in humans, irinotecan plasma concentrations decline in a multiexponential manner, with a mean terminal elimination half-life of about 6 to 12 hours. The mean terminal elimination half-life of the active metabolite SN-38 is about 10 to 20 hours. The half-lives of the lactone (active) forms of irinotecan and SN-38 are similar to those of total irinotecan and SN-38, as the lactone and hydroxy acid forms are in equilibrium. Over the recommended dose range of 50 to 350 mg/m^2, the AUC of irinotecan increases linearly with dose. Maximum concentrations of the active metabolite SN-38 are generally seen within 1 hour following the end of a 90-minute infusion of irinotecan. Irinotecan exhibits moderate plasma protein binding (30% to 68% bound). SN-38 is highly bound to human plasma proteins (approximately 95% bound). The plasma protein to which irinotecan and SN-38 predominantly bind is albumin.

How supplied

Irinotecan is supplied as a sterile, pale yellow, clear, aqueous solution. It is available in two single-dose sizes: 2 ml-fill vials contain 40 mg irinotecan hydrochloride and 5 ml-fill vials contain 100 mg irinotecan hydrochloride. Each milliliter of solution contains 20 mg of irinotecan hydrochloride (on the basis of the trihydrate salt), 45 mg of sorbitol NF powder, and 0.9 mg of lactic acid, USP. Irinotecan is intended for dilution with 5% Dextrose Injection, USP (D5W), or 0.9% Sodium Chloride Injection, USP, prior to intravenous infusion.

Storage

The intact vials would be stored at room temperature 15° to 30°C (59° to 86°F) and protected from light.

Route of administration

Irinotecan infusion must be administered intravenously by a 90 minute constant infusion.

Preparation and stability

For irinotecan doses less than 1400 mg, the amount of irinotecan to be administered will be removed aseptically from the appropriate vials and added to 500 ml of 5% Dextrose Injection, USP or 0.9% Sodium Chloride Injection, USP. For doses greater than or equal to 1400 mg, the appropriate amount of drug will be added to 750 ml of 5% Dextrose Injection, USP or 0.9%

Sodium Chloride Injection, USP. The solution is physically and chemically stable for up to 24 hours at room temperature (approximately 2° to 8°C) and in ambient fluorescent lighting.

Reported adverse events and potential risks

The following are reported toxicities for irinotecan:

Blood/Bone Marrow	Leukopenia, neutropenia, granulocytopenia, thrombocytopenia
Cardiovascular	Vasodilation, edema, circulatory failure due to diarrhea
Constitutional	Fatigue (lethargy, malaise, asthenia), fever, chills, headache, weight loss
Dermatology/Skin	Dry skin, rash/desquamation, pruritis, skin pigmentation/discoloration, pruritus, alopecia
Gastrointestinal	Constipation, nausea, dyspepsia/heartburn, anorexia, diarrhea, flatulence, stomatitis, dyspepsia, abdominal cramping, paralytic ileus, colitis, small bowel ulceration, toxic megacolon
Hepatic	Increased SGOT (AST), increased SGPT (ALT), alkaline phosphatase, bilirubin, LDH
Metabolic	Electrolyte abnormalities
Musculoskeletal	Myalgia, muscle weakness
Neurology	Confusion, dizziness, insomnia, numbness/tingling, peripheral neuropathy
Ocular	Dry eye, blurred vision
Pulmonary	Infection/pneumonia
Renal	Increased uric acid, BUN, creatnine, hematuria, glucosuria

Risks will be minimized by administration of concomitant supportive care drugs and by close monitoring of patients. Potential benefits include successful anti-cancer treatment.

Availability

Irinotecan is commercially available.

Agent ordering

Irinotecan will be ordered and dispensed as routinely done through the pharmacy.

Agent accountability

The Principal Investigator (PI), or a responsible party designated by the PI, will maintain a careful record of the inventory and disposition of all agents received**.**

3.6.2 Other agents

5-fluorouracil, leucovorin, and oxaliplatin (and trastuzumab for HER2+ patients) are commercially available, and details regarding these drugs and their administration can be found in package inserts that are accessible online. They will be obtained and administered in the same way that they are given to off-protocol patients.

4.0 Measurement of effect (Imaging)

Blood counts will be measured at baseline, and within 48 hours of each chemotherapy dose during subsequent cycles. History and physical exam, hepatic and renal function tests will be performed at baseline and within 48 hours of each chemotherapy dose, during which patients will be questioned about nausea and vomiting, mucositis, diarrhea, malaise, and appetite. To increase the accuracy of the reporting of diarrhea, patients will be given a diary to report the number of daily bowel movements (see Appendix B).

Imaging:

Patients with measurable disease (primary tumor, lymph nodes) will be assessed for objective tumor response by RECIST criteria.[41] For the purposes of this study, patients will be evaluated after completion of neoadjuvant therapy (after four cycles = 8 weeks), by CT scan of the chest/abdomen/pelvis (the best option for most patients) or MRI (e.g., for those with a severe allergy to iodinated contrast).

Response and progression will be assessed in this study by evaluation at GI tumor board, with the presence of a minimum of one gastroesophageal surgeon, one medical oncologist and one radiologist. Should this not be possible, then an ad hoc meeting of these individuals should be convened forthwith for the purposes of making this determination.
If patients are not deemed resectable after the initial phase of chemotherapy, then the patient is off study and therapy will proceed at the discretion of the treating physicians.

PET-CT PET-CT will be performed as an adjunct to CT infused staging pre/post neoadjuvant chemotherapy. At present, the low dose or attenuation correction CT portion of a combined PET-CT is not always of optimal diagnostic CT quality for use with RECIST measurements. However, if the site can document that the CT performed as part of a PET-CT is of identical diagnostic quality to a diagnostic CT (with IV and oral contrast), then the CT portion of the PET-CT can be used for RECIST measurements and can be used interchangeably with conventional CT in accurately measuring cancer lesions over time.

4.1 Measurable disease

Measurable lesions are defined as those that can be accurately measured in at least one dimension (longest diameter to be recorded) as ≥10 mm. Acceptable imaging techniques include CT scan with slice thickness ≤ 5 mm or MRI. All tumor measurements must be recorded in millimeters (or decimal fractions of centimeters).

4.2 Non-measurable disease

All other lesions (or sites of disease), including small lesions (<10 mm), are considered non-measurable disease. Bone lesions, skin lesions, leptomeningeal disease, ascites, pleural/pericardial effusions, and cystic lesions are all non-measurable.

4.3 Target lesions

All measurable lesions, up to a maximum of two lesions per organ and five lesions in total, representative of all involved organs, should be identified as **target lesions** and measured at baseline. Target lesions should be selected on the basis of their size (lesions with the longest diameter) and their suitability for accurate repeated measurements by CT or MRI. A sum of the diameters (long axis for non-nodal lesions, short axis for nodal lesions) of all target lesions will be calculated and reported as the baseline tumor size. The baseline tumor size will be used as a reference to characterize the objective tumor response and to calculate the log ratio.

4.4 Non-target lesions

All other lesions (or sites of disease) should be identified as **non-target lesions** and should also be recorded at baseline. Non-target lesions include measurable lesions that exceed the maximum numbers per organ or total of all involved organs, as well as non-measurable lesions. Measurements of these lesions are not required but the presence or absence of each should be noted throughout follow-up.

4.5 Response criteria

4.5.1 Evaluation of target lesions

Complete Response (CR): Disappearance of all target lesions. Any pathological lymph nodes (whether target or non-target) must have reduction in short axis to < 10 mm**.**

Partial Response (PR): At least a 30% decrease in the tumor size, taking as reference the baseline tumor size.

Progressive Disease (PD): At least a 20% increase in the baseline tumor size, taking as reference the smallest tumor size recorded since the treatment started, or the appearance of one or more new lesions. In addition to the relative increase of 20%, the tumor size must also demonstrate an absolute increase of at least 5 mm.

Stable Disease (SD): Neither sufficient shrinkage to qualify for PR nor sufficient increase to qualify for PD, taking as reference the smallest tumor size since the treatment started.

4.5.2 Evaluation of non-target lesions

Complete Response (CR): Disappearance of all non-target lesions and normalization of tumor marker level (CA 19-9).

Stable Disease (SD): Persistence of one or more non-target lesion(s) and/or maintenance of tumor marker level above normal.

Progressive Disease (PD): Appearance of one or more new lesions and/or unequivocal progression of existing non-target lesions.

Although a clear progression of "non-target" lesions only is exceptional, the opinion of the treating physician should prevail and the progression status should be confirmed at a later time by the principal investigator.

4.5.3 Evaluation of best overall response

The best overall response is the best response recorded from the start of the treatment until disease progression/recurrence (taking as reference for progressive disease the smallest tumor size recorded since the treatment started). The patient's best response assignment will depend on the achievement of both measurement and confirmation criteria,

Target Lesions	Non-target Lesions	New Lesions	Overall Response
CR	CR	No	CR
CR	SD	No	PR
PR	Non-PD	No	PR
SD	Non-PD	No	SD
PD	Any	Yes or No	PD
Any	PD	Yes or No	PD
Any	Any	Yes	PD

Notes:

Patients with a global deterioration of health status requiring discontinuation of treatment without objective evidence of disease progression at that time should be classified as having "symptomatic deterioration." Every effort should be made to document the objective progression, even after discontinuation of treatment.

In some circumstances, it may be difficult to distinguish residual disease from normal tissue. When the evaluation of CR depends on this determination, it is recommended that the residual lesion be investigated (fine needle aspirate/biopsy) before confirming the CR.

4.6 FDG-PET/CT Imaging Procedures, Interpretation, Analysis, and Quality Assurance

Semiquantitative analysis will be used to determine the change in FDG uptake using the SUVmax approach for PET/CT scans obtained at baseline and after completion of neoadjuvant gFOLFIRINOX. SUV analysis will be performed using the maximum voxel SUV (SUVmax) of the entire lesion to assess response to therapy. A 35% or greater decrease in SUV will constitute response to therapy. Regions of interest (ROIs) will be manually placed around the entire extent of any abnormality to include and around the most intense portion of this abnormality to determine

the average and maximum tissue activity within the ROI, respectively (SUVmax), and decay corrected to time of injection.

4.6.1 **PET/CT Imaging Procedure**
PET/CT scans will be acquired pre-therapy (within 28 days of cycle 1) and after induction chemotherapy (after C4). Patients will be injected with 7-20 mCi of 18FDG (no less than 7mCi and no greater than 20 mCi). Prior to injection, all patients will have fasted for at least 4 hours prior to injection of 18FDG to diminish physiologic glucose uptake and to reduce serum insulin levels to near basal level, thereby diminishing 18FDG uptake by organs such as the heart. Blood sugar (measured by glucometer) cannot exceed 200 mg/dL at the time of FDG-PET/CT study. If blood sugars exceed 150 mg/dL, a note should be made of that on the remarks addenda. An attempt should be initially made to control the blood glucose level by encouraging the patient to drink water and remeasuring after a short period. If the blood glucose cannot reach reasonable control on the day of the PET/CT scan, it will require the rescheduling of the PET/CT study. If the glucose level cannot be controlled (i.e., the blood glucose still exceeds 200 mg/dL), the patient will not be included in the PET portion of the study. Patients are to be kept well hydrated and IV furosemide (10 mg) may be administered to increase urinary elimination of the tracer, and minimize image artifacts caused by urinary stasis, potentially confounding the interpretation of local 18FDG uptake in the pelvis.

Whole body emission acquisition of both the pretherapy PET/CT and the Posttherapy PET/CT MUST start no less than 50 minutes and no more than 70 minutes post FDG injection with a target of 60 minutes. The exact same period of uptake time must be used for the post-induction chemotherapy PET/CT emission acquisition (no more than a +/- 10 minute difference from the basline). It is critical that post-therapy emission scans be performed in an identical way to the baseline scan with the same scanner, same scan direction (skull to thighs or thighs to skull) and same arm positioning (arms up or arms down). The field of view is to minimally encompass the region between the base of the skull and the mid-thighs. For the emission scanning, the acquisition should be performed on a PET or PET/CT system in 2D or 3D (consistent between baseline and post-induction chemotherapy scans). PET/CT may be performed with or without oral or IV contrast but must be consistent between baseline and post-induction chemotherapy scans. The PET projection data are corrected for random coincidences, scatter and attenuation in accordance with manufacturer's recommendations. Transaxial images will be reconstructed into at least 128 x 128 pixel images with a pixel size according to manufacturer's recommendations, preferably of 5 mm or less. The reconstructed PET/CT images will be displayed on a computer workstation so that transaxial, sagittal and coronal images can be simultaneously viewed.

4.6.2 Interpretation of Baseline and Post-Neoadjuvant Chemotherapy PET Scans

<u>Baseline PET/CT Scan Interpretation</u>
The baseline PET/CT scans will be interpreted by an experienced nuclear medicine physician at each participating site who will be responsible for image interpretation and clinical reporting for the local site. The images will be interpreted together with pertinent clinical findings and findings of other imaging modalities such as barium esophagogram, EUS, endoscopy and standard (i.e., contrast-enhanced) CT or MRI.

Interpreting the PET/CT scans in this fashion mimics the usual clinical situation, in which this information is incorporated into the interpretation, especially in the case of an equivocal scan finding that may be easily explained by the CT/MRI scan result (e.g., anatomic variation of the bowel or bladder) or clinical information (e.g., increased uptake at a site of recent surgery or biopsy).

Visual assessment will be used to interpret the PET/CT findings as positive or negative at baseline. Abnormal (positive) 18FDG tumor uptake using visual assessment will be defined as "any focal or diffuse FDG uptake above background that is incompatible with normal anatomy." Visual interpretation will be the primary criterion used for positivity/negativity of baseline findings, since this is currently the only validated approach for esophageal cancer. However, to objectively assess the degree of 18FDG uptake, a semi-quantitative approach using the SUV (standardized uptake value) will also be employed.

The primary reasons for determining the SUV's on the baseline PET/CT studies are:

1) Provide a baseline parameter for calculating the change in maximum SUV (SUVmax) of the primary esophageal tumor and any established/confirmed tumor metastases following induction chemotherapy.
2) Determine whether the patient will be eligible to undergo subsequent PET/CT scans for monitoring of treatment response under this protocol by establishing that at least the primary esophageal tumor is 18FDG-avid by visual interpretation. Visually the esophageal mass must have 18FDG uptake significantly above baseline (qualitative). A semiquantitative measure to assist in this assessment is that the ratio of the SUVmax of the mass to the average SUV of a region of interest respresentative of mediastinal background (to exclude hypermetabolic foci) should be greater than 1.5

The change in SUVmax between the baseline and post-therapy PET/CT studies will be calculated for all established/confirmed lesions seen by baseline PET/CT. The change in SUV of these lesions following therapy will be expressed as % decrease (or increase) in SUV from baseline to the post-neoadjuvant chemotherapy PET/CT scans.

<u>Interpretation of Post Induction Chemotherapy PET/CT Scans:</u>

The post induction chemotherapy PET or PET/CT scans will be interpreted preferably by the same PET/CT reader at each participating site who will be responsible for image interpretation. Here again, the images will be interpreted together with pertinent clinical findings and any available findings from other imaging modalities such as standard CT or MRI, as well as other imaging modalities performed at the discretion of the treating physician. Both lesions present at baseline as well as the appearance of new lesions on follow-up PET/CT will be incorporated in the interpretation. Both visual assessment and semiquantitative assessment using the SUVmax approach will be used to interpret the follow-up PET/CT findings. Semiquantitative analysis will also be used to determine the change in FDG uptake using the SUVmax approach (i.e., change in SUVmax, see below) following induction chemotherapy for correlation with clinical and histopathological response to therapy

as well as disease-free and overall survival. For assessment of new lesions that may appear following induction chemotherapy, visual interpretation, as described above, will be used to determine if the new lesion is positive or negative for tumor in conjunction with available clinical and radiological findings. A new site seen by PET/CT will only be considered disease progression if corroborated by biopsy or other established imaging methods. Patients with disease progression documented by PET/CT imaging and confirmed by biopsy or imaging should removed from protocol therapy. An increase in the SUVmax (see below) of > 20% at any time post induction chemotherapy will only be considered as possible "indication" of disease progression, unless correlated by disease progression documented by biopsy or other imaging modalities such as barium esophagram or CT, in which case disease progression will be established.

4.6.3 Semiquantitative PET/CT Analysis Using the SUV Approach

Semiquantitative analysis using the SUVmax approach will be used to assess the change in 18FDG uptake of established/confirmed baseline lesions between baseline and the two subsequent timepoints. SUV is the ratio of activity in a tissue (in μCi/ml) divided by the decay-corrected activity injected into the patient (in μCi/g). The resultant number is almost unitless (actually g/ml) and is a crude measure of degree of uptake of 18FDG into any tissue. The decision to utilize the SUVmax method is based on the fact that it is the most commonly used quantitative method in clinical FDG imaging, because of its practicality and its ease of calculation. The required information is the weight of the patient, the administered dose of 18FDG, the elapsed time from injection to midpoint of 18FDG image, and the activity in a tissue of interest determined from the PET/CT image. Importantly, this method does not require blood sampling. This parameter has also been found to be highly reproducible (mean difference between two measurements performed within a 1-week interval was about 10%), further supporting its use in serial semiquantitative analysis in PET/CT (16). SUVmax will be determined from the PET/CT images. It must be ensured that the images at all sites are reconstructed according to the algorithms described below, and with the same spatial resolutions. Regions of interest (ROIs) will be manually placed around the entire extent of any abnormality to include the most intense portion of this abnormality to determine the maximum tissue activity within the ROI (SUVmax) and decay corrected to time of injection. The tomographs will be calibrated at least monthly by imaging known activity that is also measured in a dose calibrator. Injected activity will be measured in a dose calibrator, corrected for residual activity in the syringe after injection, and decay corrected to time of injection. SUVmax will be calculated as ROI max μCi/ml divided by injected activity μCi/g body weight. The change in SUVmax between the baseline and post-therapy PET/CT studies will be calculated for all established/confirmed lesions seen by baseline PET/CT with a tumor-to-background SUV of > 1.5. The change in SUVmax of these lesions following therapy will be expressed as % decrease (or increase) in SUVmax from baseline to the post induction chemotherapy PET/CT scan. The change in SUVmax for the primary esophageal tumor identified at baseline will be calculated as well as the mean change in SUVmax for all established/confirmed lesions. These changes will be correlated with clinical and histopathological response to therapy as well as disease-free and overall survival. For lesions that have completely

resolved by PET/CT (i.e., uptake in the area of previous disease is indistinguishable from background uptake), the SUV of the lesion will be considered as 0 (no tumor, uptake is not higher than background) and % decrease from baseline calculated as 100%.

4.6.4 Quality Assurance of PET/CT data

Every effort will be made to ensure that the collected PET/CT studies performed at the various sites are acquired and processed according to the protocol guidelines.

4.7 Duration of Therapy and Followup

4.7.1 Including treatment delays due to adverse event(s), as specified in section 3.6, initial neoadjuvant preoperative chemotherapy treatment with gFOLFIRINOX will continue for 4 cycles or until one of the following criteria applies:

- Disease progression,
- Intercurrent illness that prevents further administration of treatment,
- Unacceptable adverse event(s),
- Patient decides to withdraw from the study, or
- General or specific changes in the patient's condition render the patient unacceptable for further treatment in the judgment of the investigator.

4.7.2 Following successful surgery, subsequent gFOLFIRINOX Within 5-10 weeks of surgery) will be given for 4 cycles or until one of the aforementioned criteria for stopping applies.

4.7.3 Duration of Follow Up

Patients will be followed every 3 months for 2 years and every 6 months for years 3-5 after completion or removal from study or until death, whichever occurs first. Patients removed from study for unacceptable adverse event(s) will be followed until resolution or stabilization of the adverse event.

Stable disease is measured from the start of treatment until the criteria for progression are met, taking as reference the smallest tumor size recorded since the treatment started.

4.8 Progression Free-Survival and Overall Survival

Progression free survival (PFS) is defined as the duration of time from enrollment/registration to time of progression or death from any cause.

Overall survival is defined as the duration of time from enrollment/registration to the time of death, of any cause.

4.9 Criteria for Removal from Study

Patients will be removed from study when any of the criteria listed in Section 5.4 applies. The reason for study removal and the date the patient was removed must be documented in the Case Report Form.

5.0 MEASUREMENT OF EFFECT (Pathological)

5.1 Complete R0 Resection (Co-Primary Endpoint) Cohort 1

The absence of tumor cells at the proximal and distal margins will be required to be classified as an R0 resection. T4 lesions (and therefore 'positive' circumferential margins) will be included as R0 resection if both proximal and distal margins are negative (tumor $\geq$1mm from nearest margin).

5.2 Pathologic Complete Response (pCR) (Co-Primary Endpoint) Cohort 1

Regression of the primary tumor will be documented by the amount of viable tumor versus the amount of fibrosis, ranging from no evidence of any treatment effect to a complete response with no viable tumor identified, as previously described (ref) [Grade 1a: complete remission (pCR), no residual tumor/tumor bed; Grade 1b: subtotal remission, <10% residual tumor/tumor bed; Grade 2: partial remission, 10-50% residual tumor/tumor bed; Grade 3: minor/no remission, >50% residual tumor/tumor bed]. Proximal, distal, and circumferential margins will be assessed to determine the completeness of resection.

Patients enrolled and undergoing surgery at non-Hyde Park participating sites will be asked to provide the clinical pathological slides of the resection for central review and determination/confirmation of pathologic response and resection margins. This includes sites at Silver Cross and Northshore. Patients consenting to study are agreeing to have this performed. Patients participating in the study prior to the amendment for central pathological review will have the opportunity to sign the amended consent to allow for this to occur. If patients have deceased prior to this consent opportunity, then written consent will be waived.

5.3 Pathologic Response Grade 1 (1a + 1b) Cohort 2 in intestinal type gastric body/esophagogastric cancers.

In recent analyses, outcomes of Grade 1a (complete) and Grade 1b pathologic response were similar, and therefore assessment of Grade 1 is what seems to be important with superior outcomes, as compared to Grade 2 (intermediate outcome), and Grade 3 (poor outcome), we will plan a second cohort to evaluate prospectively the Grade 1 response rate. Given that most Grade 1 responses occured in intestinal type histology, we plan to evaluate these patients in the ITT population, but will allow all patients to enroll for exploratory purposes.

6.0 Genotyping assay

A pre-registration plan will be adopted to allow UGT1A1 genotyping. Patients will be assigned to the UGT1A1Low Risk group, UGT1A1Intermediate Risk group, or UGT1A1High Risk group according to their genotype, to the completion of recruitment, and will includethe two most common groups and genotypes in North America (Low Risk *1*1 and Intermediate Risk *1*28). UGT1A1 polymorphism

analysis will be performed in a laboratory at the University of Chicago. The turnaround time for genotyping assay results is approximately 48-72 business hours.

DNA isolation will be performed from 3 ml of EDTA-blood sample from each patient using the AutoGenFlex STAR automated DNA isolation instrument (Autogen Inc.).

**Promoter Variants at Position -53 (TA)**	**Variant Haplotype**
Genotype 5 (5 TA repeats)	*36
Genotype 6 (6 TA repeats)	*1
Genotype 7 (7 TA repeats)	*28
Genotype 8 (8 TA repeats)	*37 variant
**Exon 1 SNP**	**Variant haplotype**
211 G>A	*6
364 C>T	*80

UGT1A1 phenotype groups as determined by diplotype

Phenotype	Diplotypes
**Group 1 – Low risk**<br>Extensive metabolizer	Homozygous *1, *36 or *1/ *36, or non- *80
**Group 2 – Intermediate risk**<br>Intermediate metabolizer	Heterozygous *28, *37, *6 or *80 with any of the above variants
**Group 3 – High risk**<br>Poor metabolizer	Homozygous *28, *37, *6, *80 or heterozygous *28/ *37<br>or heterozygous combinations of any of these high risk variants

The *1 and *28 alleles are the most common, and the homozygous *1/*1 diplotype and *1/*28 diplotype are the most common, and were the names of the groups previously. However, if we do encounter patients with the more rare alleles, we will be inclusive as in the above table for treatment assignment.

6.1 Genotyping of UGT1A1 polymorphisms

*UGT1A1*28*

In order to genotype the UGT1A1*28 polymorphism, approximately 40 ng of DNA will be subject to amplification by polymerase chain reaction (PCR). The amplification primers used have been previously described[42], where the sequence of the forward primer is 5'-GTCACGTGACACAGTCAAAC-3' and that of the reverse primer is 5'-TTTGCTCCTGCCAGAGGTT-3'. These primers flank the polymorphic TA locus in the promoter region of the UGT1A1 gene and amplify a 98 bp fragment when a $(TA)_6$ allele is present and a 100 bp fragment when a $(TA)_7$ allele is present. In the presence of $(TA)_5$ and $(TA)_8$ alleles, 96 bp and 102 bp alleles are amplified. The reverse primer is labeled with a fluorescent dye at its 5'-end to permit visualization of the amplification product. The amplification reactions will be performed in a 10 μl volume consisting of 1.5 mM $MgCl_2$, 250 mM dNTPs, 0.8 mM of each primer and 0.5 unit of Taq polymerase (Amplitaq Gold from Applied Biosystems, Foster City, CA). The polymerase will be activated at 95°C for 10 min and DNA amplified for 35 cycles at 95°C for 30 s, 55°C for 30 s, and 72°C for 30 s, followed by a final extension at 72°C for 10 min. Control DNAs from individuals known to have a 6/6, 6/7 and 7/7 genotype will be included in the PCR analysis. PCR amplified products will be diluted one to five in water and one microliter further diluted in 10 ul of formamide plus 1 ul of size standard (GS-500 from Applied Biosystems). Products will be run on

an ABI 3100 genetic analyzer (Applied Biosystems) and sizing performed using the Genemapper software (Applied Biosystems). UGT1A1*28 polymorphism genotyping is routinely performed in the University of Chicago Genetic Services Laboratory for clinical testing purposes.

*UGT1A1*6*
The method that has been previously published will be adopted for *6 genotyping.[43]

6.2 Sample shipment information

5 ml of patient blood sample should be drawn in a purple top EDTA tube. Samples should NOT be spun down. All samples will be appropriately labeled with patient name/ID and protocol number. In addition, all samples should be accompanied by paperwork that specifies the sample ID, protocol number, treating physician name and contact information (address, phone number and fax number) to whom the result needs to be reported to. A sample requisition form (see Appendix D) should be completed and submitted along with the blood sample for earch patient. The sample should be transported and/or shipped to the address in Appendix D on the same day whenever possible. **If the sample cannot be transported or shipped on the same day to the laboratory, it should be refrigerated at 4 °C prior to transport or shipment.**

6.3 Protection of confidentiality

Patient samples will be processed and genotyped at the University of Chicago Genetic Services Laboratory which is a CAP and CLIA certified laboratory that routinely performs genetic testing of multiple disorders on a clinical basis. The laboratory therefore has all necessary procedures related to protecting of patient confidentiality. All samples will be processed by trained technologists and results reported only to the patient's physician or other appropriate health care person. All patient samples will have a protected identification code that will be used. The primary physician will be notified of genotyping results by email and fax.

6.4 Blood banking at specified time points

Samples will be collected at four time points for all patients enrolled, including i) pre-neoadjuvant chemotherapy, ii) pre-surgery, iii) post-surgery, and iv) post-adjuvant therapy; also at a fifth point if there is disease recurrence, another banking procedure will be performed. Blood will be send to HTRC with Appendix F filled in, and banked as follows:

Hyde Park:
All subjects participating in this study will be asked to provide two vials of blood for banking future research purposes asat each time point listed above, and divided into the following for future purposes (two 10mL Streck tubes). Samples will be collected in the Duchossis Center for Advanced Medicine and Mitchell Hospital during a routine blood draw, when/if possible, or during a separate blood draw. Each sample will be accompanied by a requisition noting the IRB protocol number, patient’s name, medical record number, physician’s name, and location to send the samples to be processed immediately in the Biospecimen Bank. All samples will be stored in -80°C freezers. Each sample will be labeled with a unique suject ID of this study.

Other Participating Sites (Orland Park and Silver Cross, and Northshore)**:**
Samples obtained and processed at satellite sites above, will be sent to HTRC (address below). Please ship the samples to he University of Chicago HTRC address in Appendix F. Samples should be shipped at room temperature within 3 days from acquisition (not on Fridays) to be processed at Hyde Park HTRC.

Samples are to be labelled and should include the IRB# 14-0594, Subject study ID#, and blood timepoint (as in Appendix F) and send with Appendix F printed and filled in. Sites are to notify both Kelly Moore (kmoore8@medicine.bsd.uchicago.edu) as well as the HTRC email in Appendix F that the samples are being shipped and the tracking number recorded.

7.0 Statistical considerations

Cohort 1

The study is designed to detect a 20% improvement in complete resection rate (R0) from 70% to 90% with perioperative chemotherapy (see section 5.1), based on published surgical (range 69-74% surgery alone) and perioperative chemotherapy (range 79%-100% with neoadjuvant chemotherapy) experiences with gastric and GEJ tumors (refs).

Patient enrollment will follow an optimal two-stage design (Simon, 1989) with an alpha level of 0.05 and power of 0.90. Accrual will be halted if 11 or fewer of the initial 15 assessable patients have complete R0 resections ($\leq$73%). In the second stage, 21 additional patients will be enrolled for **a total target patient population of 36 patients**. The treatment will be considered active and worthy of additional investigation in this patient population at the end of the study if a complete resection is achieved in at least 30 of the total 36 assessable patients ($\geq$83.3%). Intention-to-treat analysis will be performed, and patients with tumor progression during/after neoadjuvant chemotherapy that precludes surgery will be included as non-R0 resection. A subset analysis will be performed evaluating the R0 rate for those patients actually undergoing surgery.

A co-primary endpoint is pathologic complete response rate (pCR) (see section 5.2). A sample size of 36 patients achieves 85% power at significance level 0.05 to detect an absolute 13% improvement using a one-sided binomial test. These results assume that the population proportion under the null hypothesis (H_0) P0= 0.03 (this rate is consistent with described rates for ECF or cisplatin/5FU chemotherapy pCR 3-4%). We will reject the null hypothesis, and accept the alternate hypothesis HA (P1 = 0.16) if there is an observed pCR in $\geq$ 4 of 36 patients ($\geq$11.1%) (this rate is consistent with pCR ranging from 10-17.4% with preoperative triple drug regimens DCF/DCX/DOF, see Table 1).

Cohort 2

Pathologic Response Grade 1 (1a + 1b) Cohort 2 in intestinal type gastric body/esophagogastric cancers.

Expansion cohort 2 sample size calculation:

We will enroll 29 new patients with intestinal type histology to reject the null hypothesis that the pathologic response (grade 1) is 20% as in historical controls, and accept the alternative hypothesis of 45% pathologic response grade 1. For this expansion cohort 2, $\geq$10 of 29 would independently confirm propectively our previous result of 11/24 (45.8%) from the original cohort 1. At completion of the expansion cohort 2, this would leave us with 53 total patients with intestinal type, with 29 of them in the prospective expansion cohort 2 and 24 in the original cohort 1. With these 53 patients pooled from cohort 1 and cohort2, this will help to provide a more precise estimate of Grade 1 pathologic response in intestinal type GEC via the 95% CI with the larger total number. We will also have more patients to more precisely estimate the R0 resection rate in this patients as evaluated in the the primary endpoint of Cohort 1.

> **Eligibility will remain the same as in the previous protocol. However, as we will have our primary statistical endpoint for the intestinal type gastric body/esophagogastric cancers only (n=29) we will still allow mixed or pure diffuse histology (as well as antral cancers as in cohort 1) to accrue for exploratory purposes, and we estimate that these non-ITT patients**

will be no more than 15 patients, so the total cohort 2 will include up to 44 patients (29 intention to treat + 15 mixed/antral tumors).

Secondary and Correlative Endpoints for both cohorts:

The secondary endpoints of response rate, surgical morbidity, and pattern of recurrence will be evaluated by reporting event rates along with exact (binomial distribution-based) 95% confidence intervals. Toxicities will be summarized by type, grade, and attribution. The secondary endpoints of OS and PFS will be estimated using the Kaplan-Meier[44]procedure and compared in the subgroups of patients with and without pCR (grade 1a) using the log-rank test.

For correlative data we will calculate Pearson or Spearman rank correlation coefficients between circulating tumor DNA derived from peripheral blood samples. The change in SUVmax between the baseline and post-therapy PET/CT studies will be analyzed lesion-by-lesion using paired t-tests or Wilcoxon, signed rank tests. Finally, the change in SUVmax for the primary esophageal tumor will be correlated with clinical and histopathological response rates by logistic regression, and with progression-free and overall survival by Cox[45] regression analysis.

8.0 Study calendar

	Pre-Study	Cycle 1	Cycle 2	Cycle 3	Cycle 4	Week +4 weeks after cycle 4 completion	Week +4-6 After cycle 4 completion	Cycle 5[e]	Cycle 6	Cycle 7	Cycle 8	Week +4 after completion of cycle 8	Off Study[d]
**Informed consent**	X												
**Demographics**	X												
**Medical history**	X		X	X	X	X		X	X	X	X	X	X
**Toxicity Assessment**			X	X	X	X			X	X	X	X	
**Physical exam**	X		X	X	X	X		X	X	X	X	X	X
**Vital signs**	X	X	X	X	X	X		X	X	X	X	X	X
**Height**	X												
**Weight**	X	X	X	X	X	X		X	X	X	X	X	X
**Performance status**	X	X	X	X	X	X		X	X	X	X	X	X
**CBC w/diff, plts**	X		X	X	X	X		X	X	X	X	X	X
**Serum chemistry[a]**	X		X	X	X	X		X	X	X	X	X	X
**Pregnancy test[b]**	X							X					
**CEA and CA 19-9**	X			X		X		X		X		X	X
**Blood Banking[i]**		X				X		X				X	X
**Radiologic evaluation CT[c]**	X					X		X				X	
**Radiologic evaluation CT/PET**	X					X							
Chemotherapy		X	X	X	X			X	X	X	X		
Surgery							Exact timing per surgeon						
For HER2+ patients: MUGA or 2D ECHO	X					X		X				X	
**Diagnostic Laparoscopy with washings[h]**	X					X[h]							
**Upper Endoscopy[f]**	X											X	
**Endoscopic Ultrasound (EUS)[g]**	X					X[g]							

a. Specific minimal lab tests required – Na, K, Mg, Phosphorus, Glucose, BUN,CO2, Chloride Creatinine, Calcium, Total Protein, Albumin, Total Bilirubin, Alkaline Phosphotase, SGOT, SGPT.
b. May be serum or urine.
c. CT scan of chest/abdomen/pelvis w/wo **or** MRI of abdomen/pelvis plus CXR if CT scan cannot be done. See follow up imaging during surveillance including minimum every 6 months for the first

three years after completing adjuvant chemotherapy and then annually during year 4 and 5 after completing adjuvant chemotherapy, then as clinically indicated after year 5.

d. Follow up visits and studies per usual standard of care but not less than every 3 months for years 1-2 and every 6 months for years 3-5.

e. Cycle 5 starts 4-10 weeks after surgery depending on patient recovery

f. Surveillance upper endoscopy after completion of adjuvant chemotherapy annually for five years

g. EUS after completion of neoadjuvant therapy performed only if clinically indicated.

h. Diagnostic laparoscopy after completion of neoadjuvant therapy performed only if clinically indicated

i. Blood banking obtained pre-neoadjuvant chemotherapy C1D1, pre-surgery around time of restaging imgaing, post-surgery around time of restaging imaging, post-adjuvant therapy around time of restaging imaging, and also at recurrence if there is recurrence (4 or 5 blood draws) to be banked per section 6.4.

Baseline serologic evaluations, exam, and assessment of performance status are to be conducted within 2 weeks prior to start of protocol therapy. UGT1A1 genotyping may occur anytime prior to registration. Baseline imaging studies must be done within 4 weeks prior to the start of therapy. One cycle consists of 14 days (2 weeks).

Participating Centers

- Northshore University Medical Center, Chicago, IL
- University of Chicago, Chicago, IL
- Silver Cross Hospital, New Lennox, IL

No data will be shared with participating sites regarding University of Chicago patients. For collaborating centers' patients, samples for genotyping and data regarding outcomes will be shared with University of Chicago investigators in a de-identified manner. Every other week conference meetings will be held for data safety and monitoring and patient accrual and treatment updates.

Reporting Requirements

ELIGIBILITY

All participating sites must submit an eligibility checklist/enrollment form (E) to the trial coordinator/data manager at the University of Chicago (Kelly Moore (kmoore8@medicine.bsd.uchicago.edu)). Kelly or other coordinator will confirm eligibility withthe University of Chicago PI (Catenacci) and communicate eligibility and results of the genotyping assay back to the participating site.

SAE REPORTING

All participating sites must report SAEs directly to their Local IRB, and to the data manager at the University of Chicago (Kelly Moore). The University of Chicago PI or desginee will submit any non-U of C SAEs to the University of Chicago Institutional Review Board if they meet the U of C IRB's SAE reporting requirements. SAEs from affiliate sites will be reported to the U of C IRB per the U of C IRBs guidelines.

Reporting Unexpected Problems involving research (violations/deviations/exceptions)

All participating sites must report violations/deviations/exceptions directly to their local IRB and to the University of Chicago PI. The University of Chicago PI or designee will submit any affiliate site's violations/deviations/exceptions to the University of Chicago IRB per the Univeristy of Chicago IRB reporting requirements.

References

1. Sehdev A, Catenacci DV. Gastroesophageal cancer: focus on epidemiology, classification, and staging. Discov Med 2013;16:103-11.
2. Sehdev A, Catenacci DVT. Perioperative therapy for locally advanced gastroesophageal cancer: current controversies and consensus of care. Journal of hematology & oncology 2013;6:66.
3. Mariette C, Piessen G, Briez N, Gronnier C, Triboulet JP. Oesophagogastric junction adenocarcinoma: which therapeutic approach? Lancet Oncol 2011;12:296-305.
4. Barreto JC, Posner MC. Transhiatal versus transthoracic esophagectomy for esophageal cancer. World J Gastroenterol 2010;16:3804-10.
5. Posner MC, Vokes, E. E., Weichselbaum, R. R. Cancer of the Upper Gastrointestinal Tract: B.C. Decker, Inc.; 1 edition (Feb 14 2002); 2002.
6. Feith M, Stein HJ, Siewert JR. Adenocarcinoma of the esophagogastric junction: surgical therapy based on 1602 consecutive resected patients. Surg Oncol Clin N Am 2006;15:751-64.
7. Kelsen DP, Winter KA, Gunderson LL, et al. Long-term results of RTOG trial 8911 (USA Intergroup 113): a random assignment trial comparison of chemotherapy followed by surgery compared with surgery alone for esophageal cancer. J Clin Oncol 2007;25:3719-25.
8. Alcindor T, Ferri LE, Marcus V, et al. Perioperative DCF chemotherapy protocol for patients with gastroesophageal adenocarcinoma: correlation between response to treatment and outcome. Med Oncol 2013;30:377.
9. Seevaratnam R, Bocicariu A, Cardoso R, et al. A meta-analysis of D1 versus D2 lymph node dissection. Gastric Cancer 2012;15 Suppl 1:S60-9.
10. Roggin KK, Hemmerich J, Posner MC. Extended follow-up after extended lymphadenectomy for gastric cancer: was it worth the wait? Lancet Oncol 2010;11:404-5.
11. Talsma K, van Hagen P, Grotenhuis BA, et al. Comparison of the 6th and 7th Editions of the UICC-AJCC TNM Classification for Esophageal Cancer. Ann Surg Oncol 2012;19:2142-8.
12. Taghavi S, Jayarajan SN, Davey A, Willis AI. Prognostic significance of signet ring gastric cancer. J Clin Oncol 2012;30:3493-8.
13. Macdonald JS. Gastric cancer: Nagoya is not New York. J Clin Oncol 2011;29:4348-50.
14. Shah MA, Ajani JA. Gastric cancer--an enigmatic and heterogeneous disease. JAMA 2010;303:1753-4.
15. Cunningham D, Allum WH, Stenning SP, et al. Perioperative chemotherapy versus surgery alone for resectable gastroesophageal cancer. N Engl J Med 2006;355:11-20.
16. Ychou M, Boige V, Pignon JP, et al. Perioperative chemotherapy compared with surgery alone for resectable gastroesophageal adenocarcinoma: an FNCLCC and FFCD multicenter phase III trial. J Clin Oncol 2011;29:1715-21.
17. van Hagen P, Hulshof MC, van Lanschot JJ, et al. Preoperative chemoradiotherapy for esophageal or junctional cancer. N Engl J Med 2012;366:2074-84.
18. Homann N, Pauligk C, Luley K, et al. Pathological complete remission in patients with oesophagogastric cancer receiving preoperative 5-fluorouracil, oxaliplatin and docetaxel. International journal of cancer Journal international du cancer 2012;130:1706-13.
19. Thuss-Patience PC, Hofheinz RD, Arnold D, et al. Perioperative chemotherapy with docetaxel, cisplatin and capecitabine (DCX) in gastro-oesophageal adenocarcinoma: a phase II study of the Arbeitsgemeinschaft Internistische Onkologie (AIO){dagger}. Annals of oncology : official journal of the European Society for Medical Oncology / ESMO 2012;23:2827-34.
20. Starling N, Okines A, Cunningham D, et al. A phase II trial of preoperative chemotherapy with epirubicin, cisplatin and capecitabine for patients with localised gastro-oesophageal junctional adenocarcinoma. British journal of cancer 2009;100:1725-30.

21. Ferri LE, Ades S, Alcindor T, et al. Perioperative docetaxel, cisplatin, and 5-fluorouracil (DCF) for locally advanced esophageal and gastric adenocarcinoma: a multicenter phase II trial. Ann Oncol 2012;23:1512-7.
22. Biffi R, Fazio N, Luca F, et al. Surgical outcome after docetaxel-based neoadjuvant chemotherapy in locally-advanced gastric cancer. World journal of gastroenterology : WJG 2010;16:868-74.
23. Chen X-L, Chen X-Z, Yang C, et al. Docetaxel, cisplatin and fluorouracil (DCF) regimen compared with non-taxane-containing palliative chemotherapy for gastric carcinoma: a systematic review and meta-analysis. PLoS One 2013;8:e60320.
24. Van Cutsem E, Moiseyenko VM, Tjulandin S, et al. Phase III study of docetaxel and cisplatin plus fluorouracil compared with cisplatin and fluorouracil as first-line therapy for advanced gastric cancer: a report of the V325 Study Group. Journal of clinical oncology : official journal of the American Society of Clinical Oncology 2006;24:4991-7.
25. Ott K, Herrmann K, Krause BJ, Lordick F. The Value of PET Imaging in Patients with Localized Gastroesophageal Cancer. Gastrointest Cancer Res 2008;2:287-94.
26. Ott K, Weber WA, Lordick F, et al. Metabolic imaging predicts response, survival, and recurrence in adenocarcinomas of the esophagogastric junction. J Clin Oncol 2006;24:4692-8.
27. Weber WA, Ott K, Becker K, et al. Prediction of response to preoperative chemotherapy in adenocarcinomas of the esophagogastric junction by metabolic imaging. J Clin Oncol 2001;19:3058-65.
28. Lordick F, Ott K, Krause BJ, et al. PET to assess early metabolic response and to guide treatment of adenocarcinoma of the oesophagogastric junction: the MUNICON phase II trial. Lancet Oncol 2007;8:797-805.
29. zum Buschenfelde CM, Herrmann K, Schuster T, et al. (18)F-FDG PET-guided salvage neoadjuvant radiochemotherapy of adenocarcinoma of the esophagogastric junction: the MUNICON II trial. Journal of nuclear medicine : official publication, Society of Nuclear Medicine 2011;52:1189-96.
30. Conroy T, Desseigne F, Ychou M, et al. FOLFIRINOX versus gemcitabine for metastatic pancreatic cancer. The New England journal of medicine 2011;364:1817-25.
31. Gourgou-Bourgade S, Bascoul-Mollevi C, Desseigne F, et al. Impact of FOLFIRINOX compared with gemcitabine on quality of life in patients with metastatic pancreatic cancer: results from the PRODIGE 4/ACCORD 11 randomized trial. Journal of clinical oncology : official journal of the American Society of Clinical Oncology 2013;31:23-9.
32. Innocenti F, Undevia SD, Iyer L, et al. Genetic variants in the UDP-glucuronosyltransferase 1A1 gene predict the risk of severe neutropenia of irinotecan. Journal of clinical oncology : official journal of the American Society of Clinical Oncology 2004;22:1382-8.
33. Marcuello E, Altes A, Menoyo A, Del Rio E, Gomez-Pardo M, Baiget M. UGT1A1 gene variations and irinotecan treatment in patients with metastatic colorectal cancer. British journal of cancer 2004;91:678-82.
34. Rouits E, Boisdron-Celle M, Dumont A, Guerin O, Morel A, Gamelin E. Relevance of different UGT1A1 polymorphisms in irinotecan-induced toxicity: a molecular and clinical study of 75 patients. Clinical cancer research : an official journal of the American Association for Cancer Research 2004;10:5151-9.
35. Ando Y, Saka H, Ando M, et al. Polymorphisms of UDP-glucuronosyltransferase gene and irinotecan toxicity: a pharmacogenetic analysis. Cancer research 2000;60:6921-6.
36. Hoskins JM, Goldberg RM, Qu P, Ibrahim JG, McLeod HL. UGT1A1*28 genotype and irinotecan-induced neutropenia: dose matters. Journal of the National Cancer Institute 2007;99:1290-5.
37. Hu Z-Y, Yu Q, Zhao Y-S. Dose-dependent association between UGT1A1*28 polymorphism and irinotecan-induced diarrhoea: a meta-analysis. European journal of cancer (Oxford, England : 1990) 2010;46:1856-65.
38. Falcone A, Ricci S, Brunetti I, et al. Phase III trial of infusional fluorouracil, leucovorin, oxaliplatin, and irinotecan (FOLFOXIRI) compared with infusional fluorouracil, leucovorin, and irinotecan (FOLFIRI) as first-line treatment for metastatic colorectal cancer: the Gruppo Oncologico

Nord Ovest. Journal of clinical oncology : official journal of the American Society of Clinical Oncology 2007;25:1670-6.
39. Cao W, Yang W, Lou G, et al. Phase II trial of infusional fluorouracil, leucovorin, oxaliplatin, and irinotecan (FOLFOXIRI) as first-line treatment for advanced gastric cancer. Anti-cancer drugs 2009;20:287-93.
40. Ychou M, Conroy T, Seitz JF, et al. An open phase I study assessing the feasibility of the triple combination: oxaliplatin plus irinotecan plus leucovorin/ 5-fluorouracil every 2 weeks in patients with advanced solid tumors. Annals of oncology : official journal of the European Society for Medical Oncology / ESMO 2003;14:481-9.
41. Eisenhauer EA, Therasse P, Bogaerts J, et al. New response evaluation criteria in solid tumours: revised RECIST guideline (version 1.1). European journal of cancer (Oxford, England : 1990) 2009;45:228-47.
42. Innocenti F, Grimsley C, Das S, et al. Haplotype structure of the UDP-glucuronosyltransferase 1A1 promoter in different ethnic groups. Pharmacogenetics 2002;12:725-33.
43. Innocenti F, Liu W, Chen P, Desai AA, Das S, Ratain MJ. Haplotypes of variants in the UDP-glucuronosyltransferase1A9 and 1A1 genes. Pharmacogenet Genomics 2005;15:295-301.
44. Kaplan EL, Meier P. Nonparametric estimation from incomplete observations. Journal of Am Stat Assn 53:457-481.
45. Cox DR. Regression models and life tables (with discussion). Journal of Royal Stat Soc B:34-187-220.
46. Hofheniz R, Hegewisch-Becker S, Thuss-Pathience PC, et al. HER-FLOT: Trastuzumab in combination with FLOT as perioperative treatment for patients with HER2-positive locally advanced esophagogastric adenocarcinoma: A phase II trial of the AIO Gastric Cancer Study Group. J Clin Oncol 32:5s, 2014 (suppl; abstr 4073)
47. FOLFIRINOX/T as first-line therapy in patients with metastatic gastroesophageal cancers (GEC). AC Lockhart, K Krajewski, A Wang-Gillam, M Amin, S Sorscher, K Lim, B Tan, J Picus, A Hecky, K Allen, JD Peterson, E O'Day, RdW Marsh, M Kozloff, BN Polite, HL Kindler, MR Sharma, DVT Catenacci. ASCO GI 2015, San Francisco.
48. Albatran et al. Perioperative chemotherapy with docetaxel, oxaliplatin, and fluorouracil/leucovorin (FLOT) versus epirubicin, cisplatin, and fluorouracil or capecitabine (ECF/ECX) for resectable gastric or gastroesophageal junction (GEJ) adenocarcinoma (FLOT4-AIO): A multicenter, randomized phase 3 trial. J Clin Oncol 35, 2017 (suppl; abstr 4004)

APPENDIX A

Performance status criteria

<table>
<tr><th colspan="2">ECOG Performance Status Scale</th><th colspan="2">Karnofsky Performance Scale</th></tr>
<tr><td>Grade</td><td>Descriptions</td><td>Percent</td><td>Description</td></tr>
<tr><td rowspan="2">0</td><td rowspan="2">Normal activity. Fully active, able to carry on all pre-disease performance without restriction.</td><td>100</td><td>Normal, no complaints, no evidence of disease.</td></tr>
<tr><td>90</td><td>Able to carry on normal activity; minor signs or symptoms of disease.</td></tr>
<tr><td rowspan="2">1</td><td rowspan="2">Symptoms, but ambulatory. Restricted in physically strenuous activity, but ambulatory and able to carry out work of a light or sedentary nature (e.g., light housework, office work).</td><td>80</td><td>Normal activity with effort; some signs or symptoms of disease.</td></tr>
<tr><td>70</td><td>Cares for self, unable to carry on normal activity or to do active work.</td></tr>
<tr><td rowspan="2">2</td><td rowspan="2">In bed <50% of the time. Ambulatory and capable of all self-care, but unable to carry out any work activities. Up and about more than 50% of waking hours.</td><td>60</td><td>Requires occasional assistance, but is able to care for most of his/her needs.</td></tr>
<tr><td>50</td><td>Requires considerable assistance and frequent medical care.</td></tr>
<tr><td rowspan="2">3</td><td rowspan="2">In bed >50% of the time. Capable of only limited self-care, confined to bed or chair more than 50% of waking hours.</td><td>40</td><td>Disabled, requires special care and assistance.</td></tr>
<tr><td>30</td><td>Severely disabled, hospitalization indicated. Death not imminent.</td></tr>
<tr><td rowspan="2">4</td><td rowspan="2">100% bedridden. Completely disabled. Cannot carry on any self-care. Totally confined to bed or chair.</td><td>20</td><td>Very sick, hospitalization indicated. Death not imminent.</td></tr>
<tr><td>10</td><td>Moribund, fatal processes progressing rapidly.</td></tr>
<tr><td>5</td><td>Dead.</td><td>0</td><td>Dead.</td></tr>
</table>

APPENDIX B

Record of bowel movements

Please keep a careful record of the number and type of bowel movements that you have while receiving drug therapy as part of the clinical trial.

Cycle #:__________

Average number of bowel movements per day before entering trial:_______

Date	Normal	Loose	Watery	Total Number of Bowel Movements

Instructions for treatment of diarrhea (defined as a watery bowel movement):

Take 4 mg (two tablets) of loperamide (Immodium AD) immediately, followed by another 2-4 mg (one to two tablets) every 3 hours until you have no further diarrhea for at least 12 hours.

APPENDIX C

CYP3A4 substrates, inhibitors, and inducers

Substrates	Documented inhibitors	Documented inducers
Acetaminophen	Bromocriptine	Carbamazepine
Amitriptyline	Cimetidine	Dexamethasone
Alprazolam	Clarithromycin	Phenobarbital
Amiodarone	Cyclosporine	Phenytoin
Astemizole	Danazol	Rifampin
Carbamazepine	Diltiazem	Troglitazone
Cisapride	Erythromycin	
Clomipramine	Ergotamine	
Clozapine	Ethinylestradiol	
Corticosteroids	Fluconazole	
Cyclosporine	Fluoxetine	
Diazepam	Fluvoxamine	
Diltiazem	Gestodene	
Erythromycin	Grapefruit	
Ethinylestradiol	Indanivir	
Felodipine	Itraconazole	
Imipramine	Ketoconazole	
Lidocaine	Miconazole	
Lovastatin	Midazolam	
Midazolam	Nefazodone	
Nefazodone	Nicardipine	
Nifedipine	Nifedipine	
Propafenone	Omeprazole	
Quinidine	Paroxetine(weak)	
Ritonavir	Progesterone	
Sertraline	Propoxyphene	
Simvastatin	Quinidine	
Terfenadine	Ritonavir	
Triazolam	Sertraline(weak)	
Verapamil	Testosterone	
Warfarin	Troleandomycin	
	Verapamil	
	Zafirlukast	
	Zileutin	

APPENDIX D

<u>GENOTYPING BLOOD SAMPLE COLLECTION FORM</u>

A genotype-guided dosing study of FOLFIRINOX in previously untreated patients with metastatic gastroesophageal adenocarcinoma

UNIVERSITY OF CHICAGO PROTOCOL # :

Patient I.D. No.: _____________

Time of sample collection: _____________

Treating physician name ____________________________________

DNA testing: <u>UGT1A1*28 and *6</u>

<u>The results need to be reported to the following people:</u>

Daniel Catenacci, MD
Email: dcatenac@medicine.bsd.uchicago.edu
Fax: 773-702-3163
Phone: 773-702-7596

Ugne Stacie Marcevicius, RN
Email: umarkevicius@medicine.bsd.uchicago.edu
Fax: 773-834-0475
Phone: 773-753-4525

Leah Chase
Clinical Trial Coordinator and Data Manager
Email: lmchase@medicine.bsd.uchicago.edu

For University of Chicago patients, please call 773-702-9358 (contact person: Larry House) for sample pick-up once the sample is collected. The sample will be transported to the University of Chicago Genetic Services Laboratory, room G-701.

For Northshore University patients, samples should be shipped to the following address and samples can be shipped at room temperature:

Soma Das, Ph.D.
University of Chicago Genetic Services Laboratory
Room G-701
5841 S. Maryland Ave
Chicago, IL 60637-1470

<u>If sample cannot be transported or shipped on the same day to the laboratory, it should be refrigerated at 4 °C prior to transport or shipment.</u>

APPENDIX E ELIGIBILITY AND ENROLMENT FORM

PROTOCOL TITLE: PERIOP-FOLFIRINOX: A pilot trial of perioperative genotype-guided irinotecan dosing of gFOLFIRINOX for locally advanced gastroesophageal adenocarcinoma (IRB14-0594)

PRINCIPAL INVESTIGATOR: Daniel Catenacci, MD

Patient Initials: **Patient ID:**

A subject will be eligible for inclusion in this study if they meet all of the following criteria:

**Inclusion Criteria**		**Supporting Documentation**
1. Does the patient have histologically confirmed locally advanced gastric (primary endpoint includes proximal and mid-body stomach) or esophagogastric adenocarcinoma?<br><br>*[Distal gastric (antral) adenocarcinomas are eligible for enrolment but will not be included in the primary analysis.]*	☐ Yes<br>☒ No	
2. Does the patient have locally advanced disease (as determined by EUS) stage >T3 and/or any T, N+ disease without metastatic disease (Mx)?	☐ Yes<br>☐ No	
3. Has the patient undergone diagnostic laparoscopy with diagnostic washings for cytology?<br><br>*[Cytology-positive and cytology-negative patients are eligible for enrolment, but only cytology-negative patients will be included in primary analyses. Gross peritoneal disease is not eligible.]*	☐ Yes<br>☐ No	
4. Has the patient's HER2 status been confirmed? *[HER2-positive and negative patients are eligible.]*	☐ Yes<br>☐ No	
5. Is the patient's cardiac ejection fraction ≥ 50% (if HER2+) as assessed by echocardiogram, MUGA scan or cardiac MRI.	☐ Yes<br>☐ No	
6. Is the patient's age ≥ 18 years?	☐ Yes<br>☐ No	
7. Is the patient's Eastern Cooperative Oncology Group (ECOG) performance status ≤ 1 (see Appendix A).	☐ Yes<br>☐ No	

8. Is the patient eligible for surgery with curative intent?	☐ Yes<br>☐ No	
9. Does the patient have adequate organ function, as defined by each of the following for laboratory testing performed within 2 weeks prior to start of therapy:<br>- Absolute neutrophil count (ANC) ≥ l250/µl<br>- Hemoglobin ≥ 9g/dL<br>- Platelets ≥ 100,000/ µl)<br>- Total bilirubin < 1.5 x upper limit of normal<br>- SGOT and SGPT < 2.5 x upper limit of normal<br>- Creatinine ≤ 1.5 x upper limit of normal.	☐ Yes<br>☐ No	
10. Has baseline CT or MRI of the chest, abdomen, and pelvis been performed within 4 weeks prior to start of therapy?<br><br>*[Measurable or non-measurable disease by RECIST 1.1 will be allowed.]*	☐ Yes<br>☐ No	
11. Has the patient's UGT1A1 genotype been determined? *[Patients with any polymorphism in UGT1A1 other than *1 or *28 (e.g, *6) will be allowed and treated as in the *28/*28 dosing group.]*	☐ Yes<br>☐ No	
12. Women of childbearing potential and men with female partners of childbearing potential must agree to use adequate contraception (hormonal or barrier method of birth control; abstinence) prior to study entry and for the duration of study participation, up until 30 days after final study treatment. Should a woman become pregnant or suspect that she is pregnant while participating in this study, she should inform her treating physician immediately.<br><br>Does the patient agree to these terms, if of reproductive potential?	☐ Yes<br>☐ No	
13. Has the patient signed the current informed consent document?	☐ Yes<br>☐ No	

EXCLUSION CRITERIA: A subject will not be eligible for inclusion in this study if any of the following criteria apply:

Exclusion Criteria		Supporting Documentation
1. Does the patient have a concurrent or previous malignancy (except for adequately treated basal cell or squamous cell skin cancer, in situ cervical cancer, or any other cancer for which the patient has been previously treated and the lifetime recurrence risk is less than 30%)?	□ Yes<br>□ No	
2. Does the patient have inflammatory bowel disease (e.g. Crohn's disease, ulcerative colitis) that is uncontrolled or being actively treated?	☐ Yes<br>☐ No	
3. Does the patient have diarrhea grade 1 or greater by the National Cancer Institute Common Terminology Criteria for Adverse Events (NCI-CTCAE, v. 4.0)?	☐ Yes<br>☐ No	
4. Does the patient have neuropathy grade 2 or greater by NCI-CTCAE, v. 4.0?	☐ Yes<br>☐ No	
5. Does the patient have a serious underlying medical or psychiatric illness that would, in the opinion of the treating physician, substantially increase the risk for complications related to treatment?	☐ Yes<br>☐ No	
6. Does the patient have active uncontrolled bleeding?	☐ Yes<br>☐ No	
7. Is the patient pregnant or breastfeeding?	☐ Yes<br>☐ No	
8. Has the patient undergone major surgery within 4 weeks of the start of treatment?	☐ Yes<br>☐ No	
9. Has the patient's UGT1A1 genotype been determined? *[Patients with any polymorphism in UGT1A1 other than *1 or *28 (e.g, *6) will be allowed and treated as in the *28/*28 dosing group.]*	☐ Yes<br>☐ No	

Genotype Group

***1/*1 (6/6 – 180 mg/m²)**	
***1/*28 (6/7 – 135 mg/m²)**	
***28/*28 (7/7 – 90 mg/m²)**	

Concomitant Medications

Is patient currently prescribed substrates, inhibitors, or inducers of CYP3A4? (see protocol Appendix C)<br><br>***Patients taking substrates, inhibitors, or inducers of CYP3A4 should be encouraged to switch to alternative drugs whenever possible, given the potential for drug-drug interactions with irinotecan.***	☐ Yes<br>☐ No

The patient meets all study eligibility criteria:

☐ **Yes**

☐ **No**

Eligibility Confirmed by:

________________________________ ______________________

Name & Signature **Date**

________________________________ ______________________

PI/ Sub-I Name & Signature **Date**

Protocol
#IRB-14-0594

Appendix F
The University of Chicago
Blood Sample Collection Form

IRB 14-0594 PERIOP-FOLFIRINOX: A pilot trial of perioperative genotype-guided irinotecan dosing of gFOLFIRINOX for locally advanced gastroesophageal adenocarcinoma

Clinician/Research Nurse: Please Fill Out and Mail with the samples at room temperature

Blood Samples

Patient Name: ____________________ **Visit Time Point:**______________________

Patient Protocol ID #:_______________ **Date Blood Obtained:** __________________

Date of Birth: ______________________ **Attending Physician:** __________________

Institution (Satellite Site): ______ ______________________________________

Date consent was signed: ____________ ______________________________

Day started on treatment on clinical protocol:__________________________________

Visit Time Point	Collection Tubes to use (ship at room temperature):	Date Shipped to HTRC Hyde Park
Cycle 1 Day 1	Two Streck Tubes	
Post-Neoadjuvant Therapy	Two Streck Tubes	
Post-Surgery	Two Streck Tubes	
Post-Adjuvant Therapy	Two Streck Tubes	
Progression	Two Streck Tubes	

Please mail this form with the samples to:

The University of Chicago
HTRC attn Hari Thomallari
5841 S. Maryland
Billings Room P-524
Chicago, IL, 60637
hthomallari@bsd.uchicago.edu
htrc.bsd.uchicago.edu
tissuebank@bsd.uchicago.edu
kmoore8@bsd.uchicago.edu
