# Supplementary material for: Evaluation of the Association of Perioperative UGT1A1 Genotype–Dosed gFOLFIRINOX With Margin-Negative Resection Rates and Pathologic Response Grades Among Patients With Locally Advanced Gastroesophageal Adenocarcinoma: A Phase 2 Clinical Trial
Source: JAMA Netw Open. 2020 Feb 14;3(2):e1921290. doi: 10.1001/jamanetworkopen.2019.21290 (PMC12549139; doi:10.1001/jamanetworkopen.2019.21290)
Supplement: Supplement 2. — eTable 1. Patient and Tumor Characteristics at Baseline in 36 Evaluable Patients Compared With the FLOT4 Study Arms eTable 2. UGT1A1 Genotype Distribution by Ethnicity in 38 Evaluable Patients eTable 3. Percentage of Total Planned Doses of Chemotherapeutic Drugs Neoadjuvantly vs Adjuvantly by UGT1A1 Genotype in 38 Evaluable Patients eTable 4. Perioperative Toxic Effects of Grade 3 or Higher Associated With gFOLFIRINOX in 38 Evaluable Patients Compared With the FLOT4 Study Arms eTable 5. Absolute Changes in SUVmax by PET in 27 Evaluable Patients and Associations With Other Clinical and Pathologic Characteristics eTable 6. Details Regarding 37 Patients Who Underwent Curative-Intent Surgery eTable 7. Surgical and Pathological Results in 36 Evaluable Patients Compared With the FLOT4 and CROSS Study Arms eTable 8. Pathological Response Grade Analysis in 36 Evaluable Patients Compared With the FLOT4 Study Arms eTable 9. Patterns of Recurrence in 11 Patients Demonstrating Metastatic Recurrence in All Patients eTable 10. Disease-Free and Overall Survival Analyses by Subgroup eFigure 1. Waterfall Plot of Percentage Change in SUVmax from Neoadjuvant gFOLFIRINOX Therapy eFigure 2. Disease-Free and Overall Survival of 36 Evaluable Patients by Histology, Primary Tumor Anatomical Site, Lymph Node Involvement at Surgery, ERBB2 Status, and UGT1A1 Subgroup eReferences. [file jamanetwopen-e1921290-s002.pdf]

Supplementary Online Content

Catenacci DVT, Chase L, Lomnicki S, et al. Evaluation of the association of perioperative *UGT1A1* genotype–dosed gFOLFIRINOX with margin-negative resection rates and pathologic response grades among patients with locally advanced gastroesophageal adenocarcinoma: a phase 2 clinical trial. *JAMA Netw Open*. 2020;3(2):e1921290. doi:10.1001/jamanetworkopen.2019.21290

eTable 1. Patient and Tumor Characteristics at Baseline in 36 Evaluable Patients Compared With the FLOT4 Study Arms

eTable 2. *UGT1A1* Genotype Distribution by Ethnicity in 38 Evaluable Patients

eTable 3. Percentage of Total Planned Doses of Chemotherapeutic Drugs Neoadjuvantly vs Adjuvantly by *UGT1A1* Genotype in 38 Evaluable Patients

eTable 4. Perioperative Toxic Effects of Grade 3 or Higher Associated With gFOLFIRINOX in 38 Evaluable Patients Compared With the FLOT4 Study Arms

eTable 5. Absolute Changes in SUVmax by PET in 27 Evaluable Patients and Associations With Other Clinical and Pathologic Characteristics

eTable 6. Details Regarding 37 Patients Who Underwent Curative-Intent Surgery

eTable 7. Surgical and Pathological Results in 36 Evaluable Patients Compared With the FLOT4 and CROSS Study Arms

eTable 8. Pathological Response Grade Analysis in 36 Evaluable Patients Compared With the FLOT4 Study Arms

eTable 9. Patterns of Recurrence in 11 Patients Demonstrating Metastatic Recurrence in All Patients

eTable 10. Disease-Free and Overall Survival Analyses by Subgroup

eFigure 1. Waterfall Plot of Percentage Change in SUVmax from Neoadjuvant gFOLFIRINOX Therapy

eFigure 2. Disease-Free and Overall Survival of 36 Evaluable Patients by Histology, Primary Tumor Anatomical Site, Lymph Node Involvement at Surgery, *ERBB2* Status, and *UGT1A1* Subgroup

eReferences.

This supplementary material has been provided by the authors to give readers additional information about their work.

© 2020 Catenacci DVT et al. *JAMA Network Open.*

eTable 1. Patient and Tumor Characteristics at Baseline in 36 Evaluable Patients Compared With the FLOT4 Study Arms

Characteristic	ECF/ECX[1] n=360	FLOT[1] n=356	gFOLFIRINOX[2] (n=36)
Median Age (Range)	62	62	66 (27-85)
Gender			
Male	265 (74%)	268 (75%)	27 (75%)
Female	95 (26%)	88 (25%)	9 (25%)
Primary Tumor Location			
EGJ Siewert type 1	85 (24%)	80 (23%)	6 (17%)
EGJ Siewert type 2 or 3	115 (32%)	118 (33%)	20 (56%)
Stomach	160 (44%)	158 (34%)	10 (28%)*
Signet Ring Cells			
Present	101 (28%)	100 (28%)	10 (28%)
Absent	234 (65%)	245 (69%)	26 (72%)
Tumor Differentiation			
G1 Well Differentiated	21 (6%)	12 (3%)	1 (3%)
G2 Moderately Differentiated	131 (36%)	123 (35%)	9 (25%)
G3 Poorly Differentiated	177 (49%)	177 (50%)	26 (72%)
Clinical T-Stage			
cT1	2 (1%)	3 (1%)	0 (0%)
cT2	59 (16%)	49 (14%)	5 (14%)
cT3	253 (70%)	267 (75%)	31 (86%)
cT4	33 (9%)	28 (8%)	0 (0%)
Clinical N-Stage			
cN- (N0)	70 (19%)	77 (22%)	9 (25%)
cN+ (N1/2/3)	290 (81%)	279 (78%)	27 (75%)
HER2-status			
Positive	NR	NR	6 (17%)
Negative			30 (83%)

*only gastric body (antrum/pylorus not eligible). NR, not reported.

© 2020 Catenacci DVT et al. *JAMA Network Open.*

eTable 2. *UGT1A1* Genotype Distribution by Ethnicity in 38 Evaluable Patients**

**Ethnicity**	**Total N=38 (%)**	**Genotype 6/6 n=19 (50%)**	**Genotype 6/7 n=16 (42%)**	**Genotype 7/7 n=3 (8%)**
**White**	31 (81.6)	17	13	1
**Black**	3 (7.9)	0	2	1
**Hispanic**	2 (5.3)*	1	0	0
**Asian (Indian)**	2 (5.3)	0	1	1

* One Hispanic patient demonstrated 5/6 genotype, the genotype 5 (allele *36) representing 5 TA repeats, considered a low risk patient.

** No patients screened/enrolled had polymorphism in the *6 Exon 1 locus.

© 2020 Catenacci DVT et al. *JAMA Network Open.*

eTable 3. Percentage of Total Planned Doses of Chemotherapeutic Drugs Neoadjuvantly vs Adjuvantly by *UGT1A1* Genotype in 38 Evaluable Patients

**Chemotherapy**	**Total N=38**	**Genotype 6/6 n=19 Irinotecan 180 mg/m2**	**Genotype 6/7 n=16 Irinotecan 135 mg/m2***	**Genotype 7/7 n=3 Irinotecan 90 mg/m2***
**Total 8 cycles**	(%)	(%)	(%)	(%)
Fluorouracil	88.0	85.3	88.9	100
Leucovorin	85.0	81.2	86.7	100
Irinotecan	82.3	74.8	82.3	75.0
Oxaliplatin	84.1	81.9	86.9	83.3
**Neoadjuvant 4 cycles**				
Fluorouracil	100	100	100	100
Leucovorin	100	100	100	100
Irinotecan	97.2	97.0	100	83.3
Oxaliplatin	100	100	100	100
**Adjuvant 4 cycles**				
Fluorouracil	75.9	70.5	77.8	100
Leucovorin	70	62.4	73.4	100
Irinotecan	58.8	52.6	64.6	66.7
Oxaliplatin	68.3	63.8	73.9	66.7
**Adjuvant 4 cycles****	N=26**	n=11	n=12	n=3
Fluorouracil	95.5	100	90.2	100
Leucovorin	96.2	100	91.7	100
Irinotecan	84.0	86.4	86.1	66.7
Oxaliplatin	89.8	95.7	90.2	66.7

*The calculation of the administered irinotecan dose of the total planned dose was weighted by genotype (ie. The data reflect the % dose of the planned dose per genotype. For example, 75% of the planned dose of 90mg/m2 was given over the 8 cycles in the genotype 7/7 group).
**Of those 26 patients who initiated adjuvant gFOLFIRINOX therapy

© 2020 Catenacci DVT et al. *JAMA Network Open.*

eTable 4. Perioperative Toxic Effects of Grade 3 or Higher Associated With gFOLFIRINOX in 38 Evaluable Patients Compared With the FLOT4 Study Arms

CTCAE Term	ECF/ECX[1] (n=354)	FLOT[1] (n=354)	gFOLFIRINOX[2] (n=38)
Diarrhea	13 (4%)	34 (10%)	7 (18.4%)
Vomiting	27 (8%)	7 (2%)	2 (5.3%)
Nausea	55 (16%)	26 (7%)	2 (5.3%)
Neuropathy	7 (2%)	24 (7%)	0 (0%)
Anemia	20 (6%)	9 (3%)	2 (5.3%)
Alopecia (Grade 2)	74 (21%)	98 (28%)	1 (2.6%)
Thrombocytopenia	11 (3%)	7 (2%)	0 (0%)
Neutropenia	139 (39%) Prophylactic G-CSF not given	181 (51%) Prophylactic G-CSF not given	0 (0%) Prophylactic G-CSF given
SAE any		215 (61%)	28 (70%)
SAE related to tx		139 (35%)	6 (15%)

Abbreviations: CTCAE, Common Terminology Criteria for Adverse Events; SAE, serious adverse event; G-CSF, granulocyte-colony stimulating factor.

© 2020 Catenacci DVT et al. *JAMA Network Open.*

eTable 5. Absolute Changes in SUVmax by PET in 27 Evaluable Patients and Associations With Other Clinical and Pathologic Characteristics

Patient Number	% Change in SUVmax	Pathologic Response Grade	UGT1A1 Genotype	HER2 Status
1	44.55%	3	*1/*28	negative
2	24.65%	3	*1/*28	negative
3	-2.44%	3	*1/*1	negative
4	-35.29%	3	*1/*1	**positive**
5	-36.96%	3	*1/*1	negative
6	-42.86%	**1b**	*1/*28	negative
7	-50.00%	**1b**	*1/*28	**positive**
8	-52.46%	**1b**	*1/*1	negative
9	-60.91%	2	*1/*1	**positive**
10	-64.90%	2	*1/*1	negative
11	-67.42%	2	*1/*1	negative
12	-73.96%	**1b**	*1/*1	negative
13	-77.30%	2	*1/*28	negative
14	-78%	**1a**	*1/*1	Negative
15	-78.70%	3	*1/*1	negative
16	-81.45%	**1b**	*1/*28	negative
17	-84.52%	**1b**	*1/*1	**positive**
18	-100%	**1b**	*1/*28	negative
19	-100%	**1a**	*1/*1	negative
20	-100%	**1b**	*1/*28	negative
21	-100%	**1b**	*1/*28	Negative
22	-100%	3	*1/*28	Negative
23	-100%	**1a**	*1/*1	**positive**
24	-100%	2	*1/*28	negative
25	-100%	2	*1/*28	**positive**
26	-100%	3	*1/*1	negative
27	-100%	2	*1/*1	negative

© 2020 Catenacci DVT et al. *JAMA Network Open.*

eTable 6. Details Regarding 37 Patients Who Underwent Curative-Intent Surgery

Surgery Performed	N (%)<br>Toxicity Cohort	N (%)<br>Efficacy Cohort
**Total**	37 (100)*	35 (100)*
Transthoracic Esophagectomy	12 (32.4)	12 (34.3)
Transhiatal Esophagectomy	9 (24.3)	9 (25.7)
Proximal Gastrectomy	2 (5.4)	2 (5.7)
Sub-Total Gastrectomy	6 (16.2)**	4 (11.4)
Total Gastrectomy	8 (21.6)	8 (22.9)
**Technique**		
Open	20 (54.1)**	18 (51.4)
Minimally Invasive Surgery-Laparoscopic	11 (29.7)	11 (31.4)
Minimally Invasive Surgery-Robotic	6 (16.2)	6 (17.1)

*One patient of 38 evaluable for toxicity and efficacy by intention to treat died prior to surgery
**These included 2 patients with antral tumors excluded from primary efficacy analyses.

© 2020 Catenacci DVT et al. *JAMA Network Open.*

eTable 7. Surgical and Pathological Results in 36 Evaluable Patients Compared With the FLOT4 and CROSS Study Arms

**Surgical Results**	**CRT CROSS[3] (n=134 (AC))**	**ECF/ECX[1] (n=360)**	**FLOT[1] (n=356)**	**gFOLFIRINOX[2] (n=36)**
Proceeded to surgery		341 (95%)	345 (97%)	35 (97%)**
Received resectional surgery	122 (91%)*	314 (87%)	336 (94%)	35 (97%)
**Rate of margin-free R0 resection ITT**	**110 (82%)***	**279 (78%)**	**301 (85%)**	**33 (92%)[#]**
Type of surgery<br>esophagogastrectomy<br>gastrectomy (total & partial)	 134 (100%) 	 98 (27%)<br>200 (56%)	 109 (31%)<br>208 (58%)	 23 (66%)<br>12 (34%)
Mean # of LN removed<br>(25%; 75% Quartile)	15	25 (19; 33)	24 (18; 32)	24 (19; 28)
**ypT-stage**<br>**≤T1**<br>T2<br>T3<br>T4<br>Tx	Not reported	 53 (15%)<br>44 (12%)<br>175 (49%)<br>47 (13%)<br>41 (11%)	 88 (25%)<br>44 (12%)<br>165 (46%)<br>37 (10%)<br>22 (6%)	 **12 (33%)**<br>4 (11%)<br>17 (47%)<br>3 (8%)<br>--
**ypN-stage**<br>**N0**<br>N1<br>N2<br>N3<br>Nx	Not reported	 146 (41%)<br>44 (12%)<br>54 (15%)<br>73 (20%)<br>43 (12%)	 174 (49%)<br>55 (16%)<br>47 (13%)<br>57 (16%)<br>23 (7%)	 **19 (53%)**<br>5 (14%)<br>6 (17%)<br>6 (17%)<br>--

Abbreviations: LN, lymph nodes; CRT, chemoradiotherapy; AC, adenocarcinoma subgroup.
* Personal communication with authors

**One patient died approximately 4 weeks after successfully completing neoadjuvant gFOLFIRINOX while awaiting surgery, deemed unrelated to chemotherapy or cancer per treating physician.

[#] One patient with R0 resection having CRT after completing gFOLFIRINOX and prior to surgery included by intention to treat.

© 2020 Catenacci DVT et al. *JAMA Network Open.*

eTable 8. Pathological Response Grade Analysis in 36 Evaluable Patients Compared With the FLOT4 Study Arms

PRG (Becker Criteria[1])	ECF/ECX[1] mITT n=137	ECF/ECX[1] ITT n=152	FLOT[1] mITT n=128	FLOT[1] ITT n=148	gFOLFIRINOX[2] mITT n=34	gFOLFIRINOX[2] ITT n=36
Grade 1a – Complete Response	8 (6%)	8 (5%)	20 (16%)	20 (13.5%)	3 (9%)	3 (8%)
Grade 1b – Subtotal Response	23 (17%)	23 (15%)	27 (21%)	27 (18%)	10 (29%)	10 (28%)
**Grade 1 – Complete or Subtotal**	**31 (23%)**	**31 (20%)**	**47 (37%)**	**47 (32%)^**	**13 (38%)**	**13 (36%)^**
Grade 2 – Partial Response	28 (20%)	28 (18%)	23 (18%)	23 (16%)	7 (21%)	9* (25%)
Grade 3 – Minimal/No Response	52 (38%)	93 (61%)	49 (38%)	78 (52%)	14 (41%)	14 (39%)
No surgery irresectable at surgery	26 (19%)		9 (7%)		--	--

Abbreviations: PRG , Pathologic Response Grade; mITT, modified intension to treat; ITT, intention to treat.

^ Grade 1 PRG for HER2-negative tumors was 33% (see Table 3), comparable with FLOT.

* One patient with Grade 2 response having CRT after completing gFOLFIRINOX and prior to surgery.

eTable 9. Patterns of Recurrence in 11 Patients Demonstrating Metastatic Recurrence in All Patients

Recurrence Location	Number of Recurrences N=11 (%)*
Esophagogastric anastomosis	0 (0)
Gastric anastomosis	2(18.1)**
Regional LNs	2(18.1)
Peritoneum	8 (72.7)
M1 lymph nodes	5 (45)
Bone	2 (18.1)
Adrenal gland	1 (9.1)
Local recurrence only	0 (0)
Local and distant recurrence	3 (27.2)
Distant recurrence only	8 (72.7)

*Percentages do not add up to 100 as patients could have multiple sites of recurrence.

**These occurred in both R1 resections in linitis plastica patients.

© 2020 Catenacci DVT et al. *JAMA Network Open.*

eTable 10. Disease-Free and Overall Survival Analyses by Subgroup

	**Disease Free Survival**	**Overall Survival**
Intention to Treat	30.1 (95% CI 15-NR)	NR (95% CI 8.3-NR)
Histology (Intestinal vs Mixed/Diffuse)	p=0.15	p=0.58
**PET response (Response ≥35% or not)**	**p=0.0027**	**p=0.019**
Primary tumor site (Esophagogastric vs Gastric Body)	p=0.18	p= 0.13
**Pathologic response (Grade 1 vs 2 vs 3)**	**p=0.0026**	**p=0.023**
Lymph Node Involvement at Surgery (Present or not)	p=0.073	p=0.3
UGT1A1 Genotype 6/6 vs 6/7 and 7/7	p=0.91	p=0.6
HER2 positive vs HER2 negative	p=0.51	p=0.3

© 2020 Catenacci DVT et al. *JAMA Network Open.*

eFigure 1. Waterfall Plot of Percentage Change in SUVmax from Neoadjuvant gFOLFIRINOX Therapy

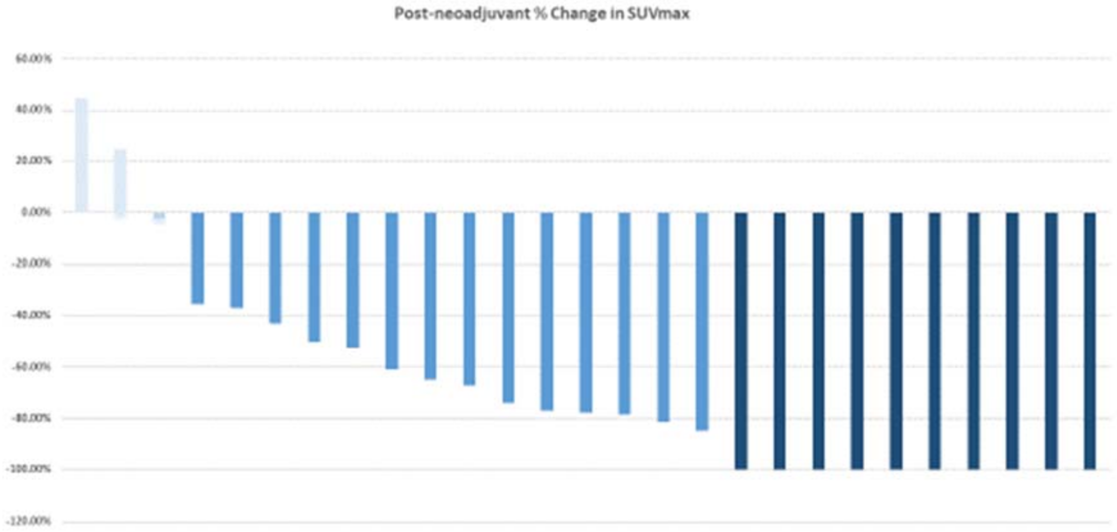

Of the 3 (11%) patients not achieving PET response, one demonstrated stable disease, and the other 2 demonstrated more than 20% increase in PET SUVmax. Of 12 patients with Grade 1 PRG and evaluable by PET, all (100%) had PET response in SUVmax of 35% of higher, and 9 (75%) had greater than 70% response in SUVmax. By histology, 20/24 (83%) patients with intestinal type were PET response evaluable, and of these 18 of 20 (90%) had PET response. All 6 patients with HER2-positive tumors achieved PET response. In contrast, only 7 of 12 (58%) diffuse/mixed histology had baseline uptake by PET and deemed assessable by PET, of which only 2 of 6 (33%) were diffuse type. Of the diffuse/mixed histology PET assessable tumors, 6 of 7 demonstrated PET response in SUVmax of 35% of higher, but only 3 (42%) of these had greater than 70% response in SUVmax, none of which were diffuse type.

© 2020 Catenacci DVT et al. *JAMA Network Open.*

eFigure 2. Disease-Free and Overall Survival of 36 Evaluable Patients by Histology, Primary Tumor Anatomical Site, Lymph Node Involvement at Surgery, *ERBB2* Status, and *UGT1A1* Subgroup

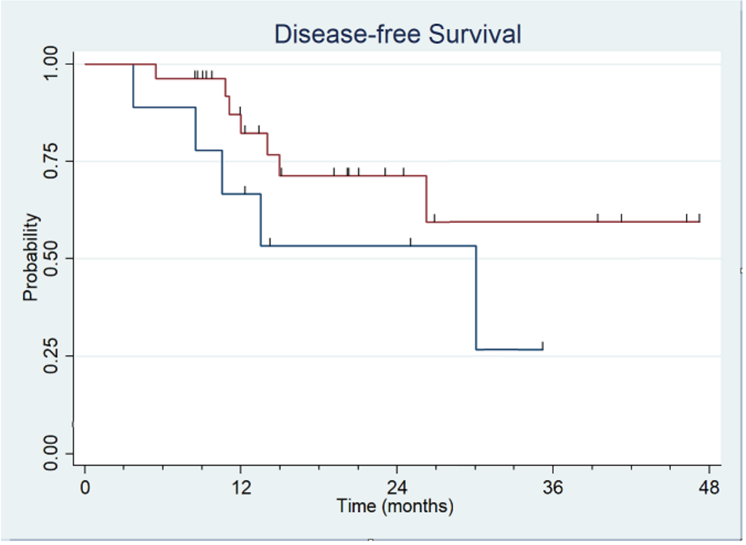

2A. Disease-Free Survival of 36 Evaluable Patients by Histology (p=0.15). Blue: Diffuse/mixed, Red: Intestinal.

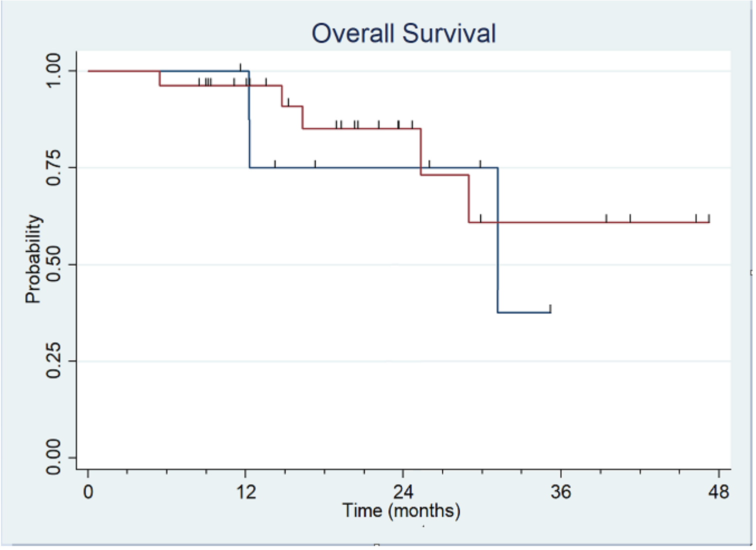

2B. Overall Survival of 36 Evaluable Patients by Histology (p=0.58). Blue: Diffuse/mixed, Red: Intestinal.

© 2020 Catenacci DVT et al. *JAMA Network Open.*

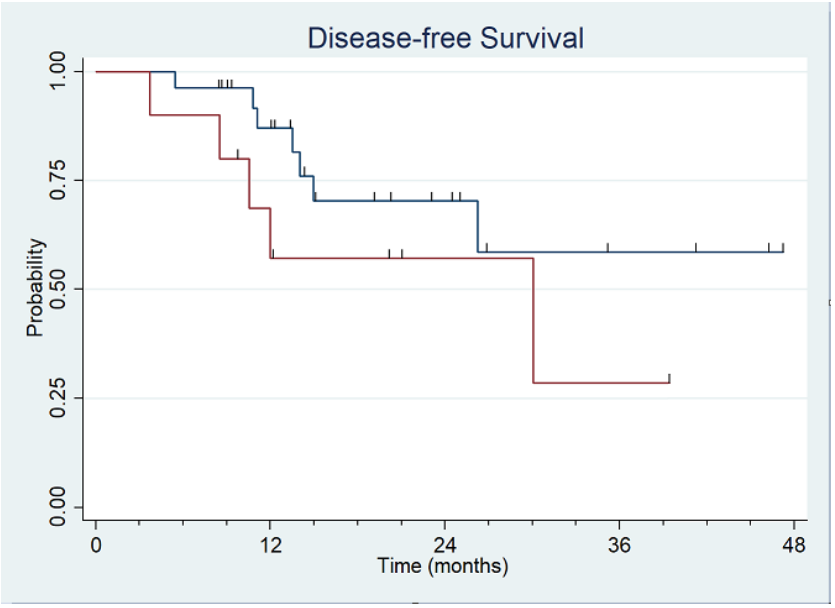

2C. Disease-Free Survival of 36 Evaluable Patients by Primary Tumor Anatomical Site (p=0.18). Blue: Esophagogastric, Red: Gastric.

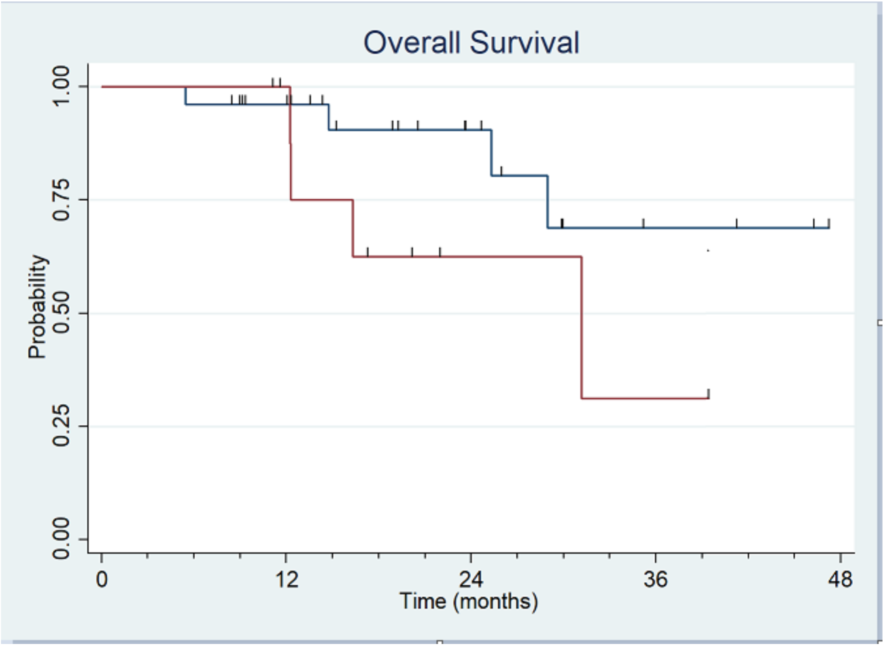

2D. Overall Survival of 36 Evaluable Patients by Primary Tumor Anatomical Site (p= 0.13). Blue: Esophagogastric, Red: Gastric.

© 2020 Catenacci DVT et al. *JAMA Network Open.*

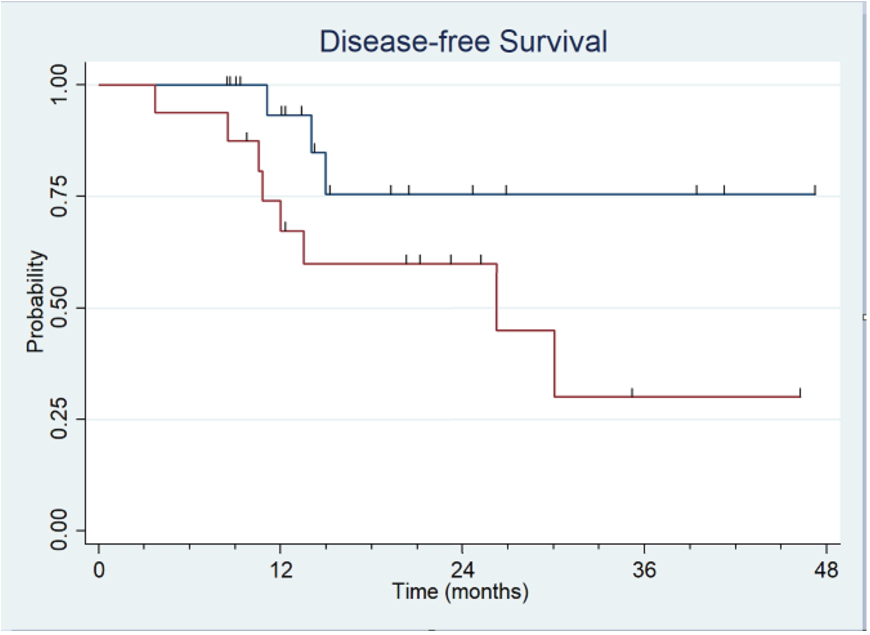

2E. Disease-Free Survival of 36 Evaluable Patients by Lymph Node Involvement at Surgery (p= 0.073). Blue: lymph node negative, Red: lymph node positive.

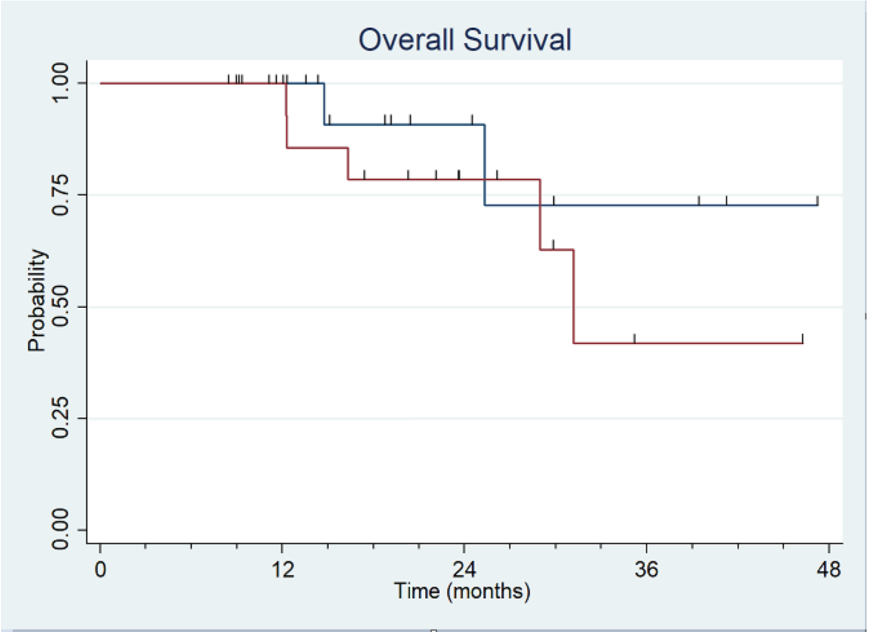

2F. Overall Survival of 36 Evaluable Patients by Lymph Node Involvement at Surgery (p= 0.3). Blue: lymph node negative, Red: lymph node positive.

© 2020 Catenacci DVT et al. *JAMA Network Open.*

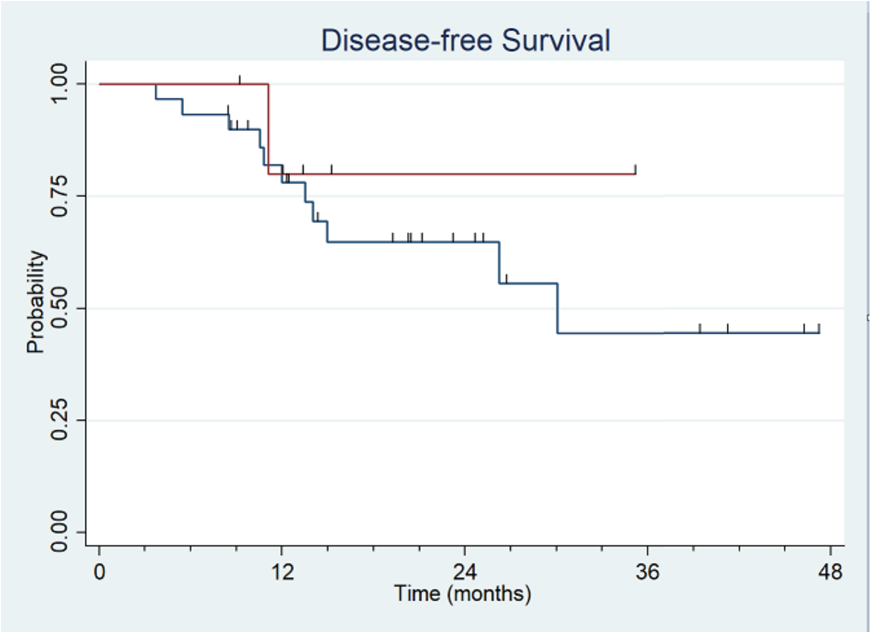

2G. Disease-Free Survival of 36 Evaluable Patients by HER2 status (p= 0.51). Blue: HER2 negative, Red: HER2 positive.

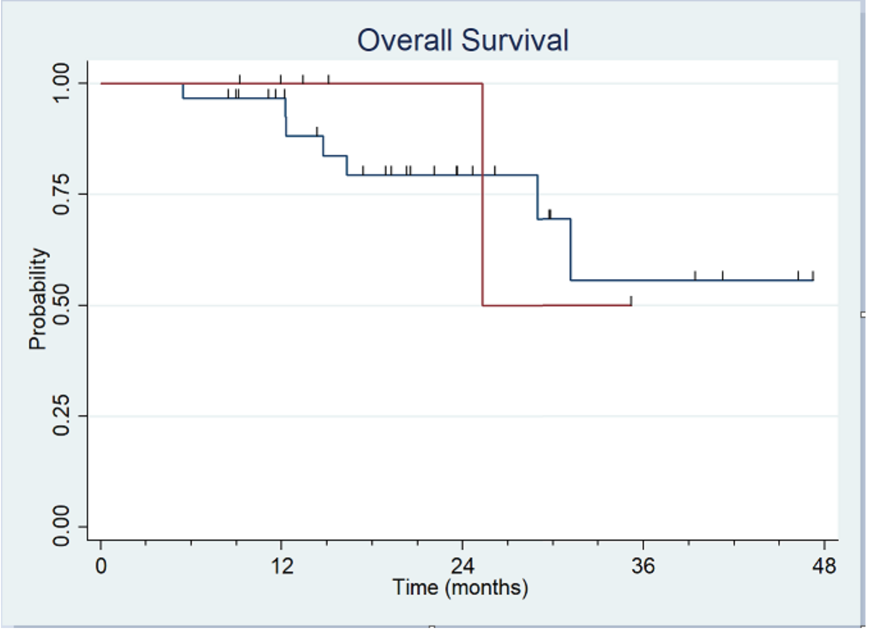

2H. Overall Survival of 36 Evaluable Patients by HER2 status at Surgery (p= 0.3). Blue: HER2 negative, Red: HER2 positive.

© 2020 Catenacci DVT et al. *JAMA Network Open.*

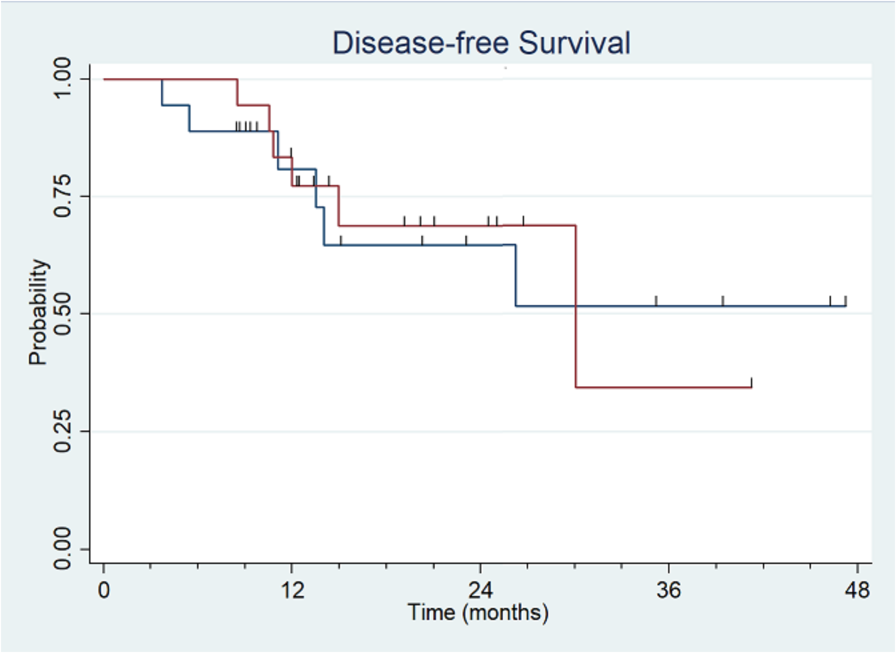

2I. Disease Free Survival of 36 Evaluable patients by UGT1A1 subgroup. Genotype group *1/*1 (6/6) compared to genotypes *1/*28 and *28/*28 (6/7 and 7/7) demonstrated no significant differences (p=0.91). Blue: Genotype *1/*1, Red: Other Genotype.

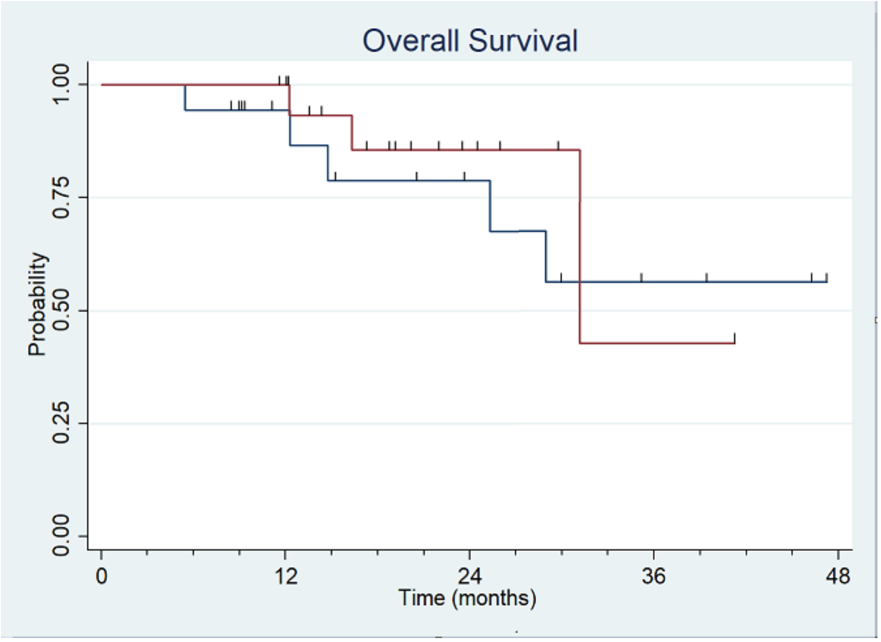

2J. Overall Survival of 36 Evaluable Patients by UGT1A1 subgroup. Genotype group *1/*1 (6/6) compared to genotypes *1/*28 and *28/*28 (6/7 and 7/7) demonstrated no significant differences (p=0.6). Blue: Genotype *1/*1, Red: Other Genotype.

© 2020 Catenacci DVT et al. *JAMA Network Open.*

eReferences.

1. Al-Batran SE, Homann N, Pauligk C, et al. Perioperative chemotherapy with fluorouracil plus leucovorin, oxaliplatin, and docetaxel versus fluorouracil or capecitabine plus cisplatin and epirubicin for locally advanced, resectable gastric or gastro-oesophageal junction adenocarcinoma (FLOT4): a randomised, phase 2/3 trial. *Lancet* 2019; **393**(10184): 1948-57.
2. Catenacci D, Chase L, Lomnicki S. et al. Perioperative (P) UGT1A1 genotype guided irinotecan (iri) dosing 'gFOLFIRINOX' for gastroesophageal adenocarcinoma (GEA). *J Clin Oncol 2014;37(15)* 2019.
3. Shapiro J, van Lanschot JJB, Hulshof M, et al. Neoadjuvant chemoradiotherapy plus surgery versus surgery alone for oesophageal or junctional cancer (CROSS): long-term results of a randomised controlled trial. *Lancet Oncol* 2015; **16**(9): 1090-8.

© 2020 Catenacci DVT et al. *JAMA Network Open.*
