# Supplementary material for: Evaluation of the Association of Perioperative UGT1A1 Genotype–Dosed gFOLFIRINOX With Margin-Negative Resection Rates and Pathologic Response Grades Among Patients With Locally Advanced Gastroesophageal Adenocarcinoma: A Phase 2 Clinical Trial
Source: JAMA Netw Open. 2020 Feb 14;3(2):e1921290. doi: 10.1001/jamanetworkopen.2019.21290 (PMC12549139; doi:10.1001/jamanetworkopen.2019.21290)
Supplement: Supplement 3. — Data Sharing Statement [file jamanetwopen-e1921290-s003.pdf]

Data Sharing Statement

Catenacci. Evaluation of the Association of Perioperative UGT1A1 Genotype-Dosed gFOLFIRINOX With Margin-Negative Resection Rates and Pathologic Response Grades Among Patients With Locally Advanced Gastroesophageal Adenocarcinoma. *JAMA Netw Open*. Published February 14, 2020. 10.1001/jamanetworkopen.2019.21290

Data
Data available: No
